# Genetic architecture of gene regulation in Indonesian populations identifies QTLs associated with global and local ancestries

**DOI:** 10.1016/j.ajhg.2021.11.017

**Published:** 2021-12-16

**Authors:** Heini M. Natri, Georgi Hudjashov, Guy Jacobs, Pradiptajati Kusuma, Lauri Saag, Chelzie Crenna Darusallam, Mait Metspalu, Herawati Sudoyo, Murray P. Cox, Irene Gallego Romero, Nicholas E. Banovich

**Affiliations:** 1 Center for Evolution and Medicine, School of Life Sciences, Arizona State University, Tempe, AZ 85281, USA; 2 The Translational Genomics Research Institute, Phoenix, AZ 85004, USA; 3 Statistics and Bioinformatics Group, School of Fundamental Sciences, Massey University, Palmerston North 4410, New Zealand; 4 Centre for Genomics, Evolution and Medicine, Institute of Genomics, University of Tartu, Tartu 51010, Estonia; 5 Leverhulme Centre for Human Evolutionary Studies, Department of Archaeology, University of Cambridge, Cambridge CB2 1QH, UK; 6 Complexity Institute, Nanyang Technological University, Singapore, 637460; 7 Laboratory of Genome Diversity and Disease, Eijkman Institute for Molecular Biology, Jakarta 10430, Indonesia; 8 Institute of Genomics, University of Tartu, Tartu 51010, Estonia; 9 Melbourne Integrative Genomics, University of Melbourne, Parkville, VIC 3010, Australia; 10 School of BioSciences, University of Melbourne, Parkville, VIC 3010, Australia; 11 Centre for Stem Cell Systems, University of Melbourne, Parkville, VIC 3010, Australia

**Keywords:** eQTL, methylation QTL, DNA methylation, gene expression, colocalization, ancestry, local ancestry, diverse populations, Indonesia, genomics

## Abstract

Lack of diversity in human genomics limits our understanding of the genetic underpinnings of complex traits, hinders precision medicine, and contributes to health disparities. To map genetic effects on gene regulation in the underrepresented Indonesian population, we have integrated genotype, gene expression, and CpG methylation data from 115 participants across three island populations that capture the major sources of genomic diversity in the region. In a comparison with European datasets, we identify eQTLs shared between Indonesia and Europe as well as population-specific eQTLs that exhibit differences in allele frequencies and/or overall expression levels between populations. By combining local ancestry and archaic introgression inference with eQTLs and methylQTLs, we identify regulatory loci driven by modern Papuan ancestry as well as introgressed Denisovan and Neanderthal variation. GWAS colocalization connects QTLs detected here to hematological traits, and further comparison with European datasets reflects the poor overall transferability of GWAS statistics across diverse populations. Our findings illustrate how population-specific genetic architecture, local ancestry, and archaic introgression drive variation in gene regulation across genetically distinct and in admixed populations and highlight the need for performing association studies on non-European populations.

## Graphical abstract

**Figure:**
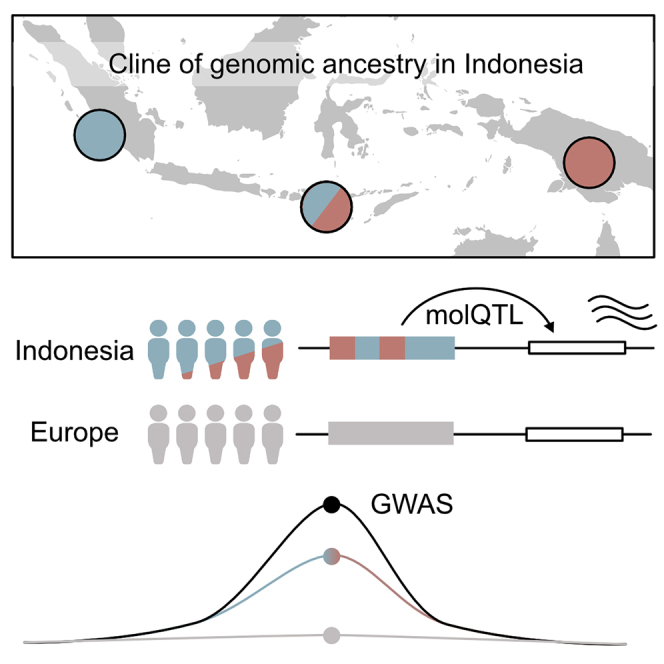

## Introduction

As we move into the age of precision medicine, the systematic undersampling of global genetic diversity limits our ability to broadly apply biomedical research efforts across diverse ethnicities and population backgrounds.^1^^,^^2^ Indeed, the vast majority of human genomics studies to date have been conducted in individuals with European ancestry, who account for a minority of the global population.^3^ To gain a comprehensive understanding of the genetic architecture of complex diseases and deliver on the promise of genomic medicine, it is critical to expand human genomics studies into diverse populations. The collection of multi-modal genomic data from traditionally undersampled populations will allow for the mapping of genetic associations with molecular phenotypes and integration with genome-wide association studies (GWASs) to fully understand the degree to which population differences impact genetic architecture.^4^

The Indonesian archipelago is one such undersampled region, absent from all existing large-scale catalogs of human diversity. Genetically and geographically structured, with a genomic cline of Asian to Papuan ancestry stretching from west to east,^5^^,^^6^ Indonesia is the fourth largest country in the world by population. Its tropical location makes it an epicenter of infectious disease diversity both past and present, making it possible that individuals from the region have adapted to local immune challenges over evolutionary time.^7^ We have previously described differences in gene expression and CpG methylation between Indonesian island populations associated with their genome-wide proportions of Papuan ancestry.^8^ To investigate the effects of modern and archaic local ancestry on gene regulation in Indonesians, here we integrate genome-wide genotype data with gene expression and DNA methylation measurements from 115 Indonesian individuals. Using this rich multi-modal dataset, we construct maps of eQTLs and methylQTLs and identify variants contributing to population differences—both within Indonesia and globally—in regulatory architecture.

## Material and methods

### Ethical approvals and dataset description

All samples were obtained from adult human male subjects. For full information about the new and published samples used in this study, refer to Tables S1 and S2. All samples used in this study were previously collected by H.S., J. Stephen Lansing, and an Indonesian team from the Eijkman Institute for Molecular Biology, Jakarta, Indonesia, with the assistance of Indonesian Public Health clinic staff. Collections followed protocols for the protection of human subjects established by institutional review boards at the Eijkman Institute (EIREC #90 and EIREC #126) and the University of Melbourne (Human Ethics Sub-Committee approval 1851639.1). All individuals gave written informed consent for participation in the study. Permission to conduct research in Indonesia was granted by the Indonesian Institute of Sciences and by the Ministry for Research, Technology, and Higher Education. Whole blood sample collection was carried out as described.^8^ The gene expression and methylation data were previously published.^8^

Here, we report two new genomic datasets: (1) 42 samples genotyped with the Illumina Infinium Omni2.5-8 v1.3 BeadChip array, including five Korowai samples from New Guinea, 18 samples from Mentawai, western Indonesia, and 19 samples from Sumba, eastern Indonesia and (2) complete genomes for 70 samples, including 11 Korowai, 30 Mentawai, and 29 Sumba samples.

### Whole-genome sequencing and data processing

Whole blood DNA from all individuals was extracted with Gentra Puregene for human whole blood kit (QIAGEN) and MagAttract HMW DNA kit (QIAGEN) according to the manufacturer’s instructions. Approximately 1.3 μg of DNA from each of the 73 individuals were sent to Garvan and sequenced with TruSeq Nano v2.5 to an expected mean depth of 30×.

The newly generated genome sequences were processed closely following a previously described protocol^6^ with the resources of the University of Tartu High Performance Computing Center.^9^ Briefly, we first aligned the reads to the “decoy” version of the GRCh37 human reference sequence (hs37d5). After alignment, and keeping only properly paired reads that mapped to the same chromosome, the autosomal sequencing depth across the samples used in downstream analyses was as follows: min = 31.5×, Q1 = 35.3×, median = 36×, Q3 = 36.5×, max = 39.5×. Base-calling was undertaken with GATK best practices.^10^^,^^11^ Following the generation of per-sample gVCF files with GATK4 HaplotypeCaller, single sample gVCFs were combined into multi-sample files with CombineGVCFs, and joint genotyping was performed with GATK4 GenotypeGVCFs, outputting all sites to a multi-sample VCF. To maximize the SNP discovery and phasing power, we used approximately 900 complete genomes in a multi-sample calling pipeline. In addition to the newly generated genomes, these included complete genome sequences from SGDP^12^ and IGDP^6^ projects, Malaspinas et al.,^13^ Vernot et al.,^14^ Lan et al.,^15^ and the HiSeqX Diversity Cohort of Polaris project (web resources) as well as approximately 100 unpublished genome sequences from Estonia and Papua. SNP calling was performed on the combined dataset and published genomes were analyzed from raw reads exactly as they were for the new sequence data. Using bcftools v1.9,^16^ we applied the following filters to each genotype call in multisample VCF files: base depth (DP) ≥ 8× and ≤ 400× and genotype quality (GQ) ≥ 30. Only bi-allelic SNPs and invariable reference sites were kept.

The published data included seven Korowai and ten Mentawai samples, however, two first-degree relatives (MTW-024 and MTW-066) were excluded from further analysis.^6^ Our final whole-genome sequencing (WGS) dataset, therefore, included 84 samples from three target groups: 17 Korowai, 38 Mentawai, and 29 Sumba.

Next, modern human multi-sample VCF files were merged with two archaic individuals: Denisovan^17^ and Neanderthal.^18^ Positions with missing or low-quality calls (marked as “LowQual” in the original archaic VCF files) in one of the archaic samples were excluded during the merging procedure. We kept only sites that had high-quality variant calls in at least 99% of samples in the combined modern/archaic dataset. Applying this 99% call-rate filter yielded a total of 52,443,217 SNPs. However, we removed sites within segmental duplications, repeats, and low complexity regions, thus retaining 49,374,343 SNPs. These masks were downloaded from the UCSC and Broad Institute genome resources (web resources). Phasing was performed with Eagle v2.4.^19^ Because our final dataset included complete genomes from very diverse human populations together with a large number of local West Island Southeast Asian and Papuan groups, we did not use any reference datasets to avoid potential phasing bias.

### Genotype array data processing

Approximately 1 μg of DNA from each of 42 individuals were sent to Macrogen for genotyping on the Illumina Infinium Omni2.5-8 v1.3 BeadChip array. Samples were processed according to the manufacturer’s instructions. Array data was processed in PLINK v1.9.^20^ The average missing rate per person in the raw dataset was around 0.45% (min 0.27%, max 2.5%); 2,194,297 autosomal positions were kept after excluding SNPs with more than 5% of missing data.

Array data were imputed with Beagle v5.1^21^ with complete genome sequences as a reference. Two imputation reference panels were generated containing both published^6^ and unpublished data. For the imputation of 18 Mentawai samples, we applied a reference panel that included 97 complete genome sequences from western Indonesia (Bali, Borneo, Java, Mentawai, Nias, Sulawesi, and Sumatra), the Philippines, and Taiwan. For the imputation of 24 Korowai and Sumba samples, we applied a reference panel made of 249 complete genomes sequence from eastern Indonesia (Alor, Flores, Kei, Lembata, Sumba, and Tanimbar) and Papua (Bougainville, New Britain, New Guinea, including Korowai, and New Ireland).

We filtered variant sites with bcftools and VCFtools^22^ to retain only high-quality imputed sites with dosage R^2^ > 0.95 (estimated squared correlation between the estimated allele dose and the true allele dose, DR2). We extracted these positions from the complete genomes from Korowai, Mentawai, and Sumba (n = 84) to produce a new combined SNP set made of imputed and WGS data. We filtered these data to retain SNPs with a proportion of missing data < 0.3 and minor allele frequency (MAF) > 0.05, which resulted in 4,077,164 variants. Imputed genotypes were further filtered to retain genotypes with genotype probability (GP) > 0.90.

### RNA sequencing and data processing

RNA sequencing and data processing were carried out as previously described.^8^ Whole blood RNA was collected and extracted with the Tempus Blood RNA tube and Tempus Spin RNA Isolation Kit (Invitrogen). The quality and concentration of all extracted RNA samples were assessed with a Bioanalyzer 2100 (Agilent) and a Qubit device (Life Technologies). We selected samples for sequencing on the basis of their RIN (RNA integrity number) by focusing on villages with at least 10 samples with RIN ≥ 6. Library preparation was performed by Macrogen (South Korea) with 750 ng of RNA and the Globin-Zero Gold rRNA Removal Kit (Illumina) according to the manufacturer’s instructions. Samples were sequenced with a 100 bp paired-end configuration on an Illumina HiSeq 2500 to an average depth of 30 million read pairs per individual in three batches (Table S2).

FASTQ read files underwent quality control with FastQC v0.11.5 (web resources), and leading and trailing bases below a Phred score of 20 were removed with Trimmomatic v0.36.^23^ Reads were aligned to the human genome (GRCh38 Ensembl release 90, web resources) with STAR v2.5.3a^24^ and a two-pass alignment mode. Read counts were quantified with featureCounts v1.5.3^25^ against a subset of GENCODE basic (release 27) annotations^26^ (web resources) that included only transcripts with support levels 1–3. Coordinates were converted to hg19 with the R package liftOver v1.8.0 (web resources). Gene expression data were filtered to retain 12,539 genes with FPKM (fragments per kilobase of transcript per million mapped reads) > 0.1 and a read count of >6 in at least 50 samples. The distributions of FPKM in each sample and gene were transformed into the quantiles of the standard normal distribution.

### DNA methylation data processing

1 μg of DNA from each sample was shipped to Macrogen, bisulfite-converted, and hybridized to Illumina EPIC BeadChips according to the manufacturer’s instructions. We randomized samples with respect to village and island across two array batches, and three samples were processed on both batches to control for technical variation (Table S1). DNA methylation data were obtained and processed as previously described^8^ with *minfi* v1.30.0^27^ We combined and preprocessed the two arrays to correct for array background signal. Signal strength across all probes was evaluated and probes with signal p < 0.01 in >75% of samples were retained. To avoid potential spurious signals due to differences in probe hybridization affinity, we discarded 6,072 probes overlapping known SNPs segregating in any of the study populations based on previously published genotype data.^6^ The final number of probes retained was 859,404. Subset-quantile within array normalization (SWAN) was carried out with the “preprocessSWAN” function.^28^ Methylated and unmethylated signals were quantile normalized with *lumi* v2.36.0.^29^

### Local ancestry inference

We used ChromoPainter v2^30^ (CP) to perform local ancestry (LA) inference and detect Asian and Papuan ancestry in all published and newly generated complete genomes from Korowai (n = 17), Mentawai (n = 38), and Sumba (n = 29). This method relies on phased haplotype data and describes each individual recipient chromosome as a mixture of genetic blocks from the set of predefined donor individuals.

First, East Asian and Papuan reference panels were generated to assign local genomic ancestry in target samples. We selected unadmixed East Asian and Papuan samples by running ADMIXTURE v1.3^31^ at K = 3 with all available East and Southeast Asian, European, and Papuan samples from the combined WGS dataset. For the East Asian reference panel, we kept only Asian samples (n = 102) with less than 0.05% of non-East Asian ancestry. For the Papuan reference panel, we kept only Papuan samples (n = 63) with less than 0.05% of non-Papuan ancestry and excluded all Korowai samples. To balance the sample size of the two reference panels, we randomly selected 63 East Asian samples from the unadmixed reference dataset.

Next, we painted each of 84 target genomes individually with the East Asian and Papuan reference panels as donors. We used the following protocol.

(1) We performed the initial CP run with ten expectation-maximization steps to estimate prior copying probabilities for each individual and chromosome separately.
(2) Estimated prior copying probabilities were averaged across the genome for each individual. The main CP run was performed with a recombination scaling constant and global mutation probability from the first step and genome-wide average prior copying probability.
(3) Either East Asian or Papuan ancestry was then assigned to individual SNPs with a probability threshold of 0.85. Unknown ancestry was assigned to SNPs with intermediate copying probability.

### Identifying archaic introgression

We defined the high-confidence Denisovan archaic haplotypes as outlined previously^6^ but with a larger group of sub-Saharan African individuals (61 sub-Saharan Africans in total, Table S3) For each individual, we started with Denisovan-introgressed haplotypes as inferred by CP, then filtered out those that did not overlap (by >0.001%) the Denisovan-introgressed haplotypes as inferred by a previously published hidden Markov model (HMM),^6^ then filtered out those that did not overlap (by >0.001%) archaic introgressed haplotypes inferred by another HMM approach,^32^ and finally filtered out any of the remaining haplotypes that did overlap (by >0.001%) Neanderthal-introgressed haplotypes as inferred by CP. We then annotated each SNP found in several target sample groups (i.e., monomorphic SNPs in that group are skipped, as are any that are masked out by the alignability/gap mask) according to how often the reference/alternative (REF/ALT) state appears on an inferred high-confidence Denisovan-introgressed haplotype in that group. This was done for three separate groups: (1) all Korowai individuals, (2) all Korowai individuals and Sumba individuals and those Mentawai individuals who are from the new dataset, and (3) all individuals in the “Papuan” continental group, which includes all Papuans and Melanesians except Baining. We used an analogous process to annotate Neanderthal ancestry SNPs, beginning instead with Neanderthal-introgressed haplotypes inferred by CP before requiring intersection with Neanderthal-introgressed haplotypes inferred by the HMM and archaic haplotypes inferred by HMM_Archaic_ and removing those intersecting CP Denisovan haplotypes.

### Accounting for population structure and non-genetic sources of variation in the QTL analyses

Principal-component analysis (PCA) of the genotype data was carried out with the R package *SNPRelate* v1.18.1.^33^ We included five genotype principal components (PCs) as covariates in QTL analyses to account for population structure. We used a probabilistic estimation of expression residuals^34^ (PEER) to infer hidden sources of variation in expression and methylation data. These latent factors were used as surrogate variables for unknown technical batch effects and included as covariates in the QTL analyses. 29 hidden factors (25% of the number of samples) were included in models, as recommended in Stegle et al. (2012)^34^ (for technical details, see Stegle et al. [2010]^35^).

### eQTL and methylQTL analyses

Variant effects on gene expression and CpG methylation were identified by linear regression as implemented in QTLtools.^36^ Genotype, gene expression, and methylation data were available for 115 individuals: 48 Mentawai, 48 Sumba, and 19 Korowai (Tables S1 and S2). Variants within 1 Mb of the gene/CpG under investigation were considered for testing. p values of top associations adjusted for the number of variants tested in *cis* were obtained from 10,000 permutations. We calculated false discovery rate (FDR)-adjusted p values to adjust for multiple phenotypes tested. Significant associations were selected with an FDR-adjusted p value threshold of 0.01. Nominal p values for all sites within the *cis*-window were obtained with the QTLtools nominal pass. QTL power calculations were carried out with the R package *powerEQTL* v0.1.7.^37^

### Variant annotation and variant set enrichment analyses

To understand the genomic context of the putative eQTLs and methylQTLs, we annotated top SNPs from the permutation-based analyses and the target CpGs of methylQTLs by using the R package *annotatr* v1.10.0.^38^ Genic annotations (1–5 kb upstream of the transcription start site (TSS), the promoter [<1 kb upstream of the TSS], 5ʹ UTR, first exons, exons, introns, coding sequences [CDS], 3ʹ UTR, and intergenic regions) were obtained with the *TxDb.Hsapiens.UCSC.hg19.knownGene* R package v3.2.2 (web resources), CpG annotations with the *AnnotationHub* R package v2.16.1 (web resources), and enhancer annotations from FANTOM5.^39^

We tested for the enrichment of the eQTL and methylQTL variants among genomic features by using the *VSE* R package v0.99.^40^ A null-distribution was constructed on the basis of 100 matched random variant sets. Consolidated chromatin immunoprecipitation sequencing (ChIP-seq) peaks for histone marks derived from primary mononuclear cells from peripheral blood were downloaded from the NIH Epigenomics Roadmap FTP site.^41^ Additionally, annotations for DNaseI hypersensitivity peaks and histone marks for K562 and GM12878 cell lines were downloaded from the ENCODE portal.^42^

We tested for the overrepresentation of the population-specific eGenes among Gene Ontology (GO) terms and canonical pathways by using *clusterProfiler* 3.14.3.^43^ We used a background set of all eGenes to test for overrepresentation.

### eQTL-methylQTL colocalization analysis

We used a Bayesian test, as implemented in the R package *coloc* v4,^44^^,^^45^ to assess the probability of colocalization of methylQTL and eQTL signals between 3,057 pairs of CpGs and genes. We used masking to allow for multiple causal loci for each trait. Masking implemented in *coloc* allows for multiple causal variants per trait with the assumption that if multiple causal variants exist for any individual trait, they are in linkage equilibrium. All SNPs independently associated within a dataset were identified with the function “finemap.signals.” For the pairs of CpGs and genes with multiple signals, colocalization analysis was performed for each pair of signals, restricting the search space to SNPs not in linkage disequilibrium (LD) with any-but-one of each signal SNP. The p value threshold for calling a signal was set to 1 × 10^−6^, and the maximum r^2^ between two SNPs for them to be considered independent was 0.01.

Pairs with the posterior probability for a common causal variant (CCV) > 0.8 and the ratio of the posterior probability for a CCV and different causal variants (DCVs) CCV/DCV > 5 were considered to show strong evidence of colocalization. As the posterior probability for colocalization is dependent on the prior probability, we used the coloc post hoc sensitivity analysis to determine the range of prior probabilities (1.0 × 10^−8^ to 1.0 × 10^−4^) for which colocalization is supported. Pairs passing the colocalization threshold with a range of ppCCV values from <1.0 × 10^−6^ to 1.0 × 10^−4^ (lower bound of ppCCV below 1.0 × 10^−6^) were considered as showing robust support for colocalization.

### eQTL sharing with European eQTLs

Similarly to eQTL-methylQTL colocalization, we used *coloc* v4 to test for colocalization between 3,300 permutation-based eQTLs detected here with an FDR-adjusted p < 0.10 and three European whole blood eQTL studies: GTEx^46^ (n = 670), the Estonian Biobank cohort^47^ (n = 491), and Twins UK^48^ (n = 384). The European eQTL summary statistics were obtained from the EBI eQTL catalog.^49^ The methods used to call the eQTLs in the EBI eQTL catalog are comparable to the methods used in this study. Out of the 3,300 genes selected for testing, 3,049 were present in the European data and had shared variants with the Indonesian data. We identified colocalized genes with the threshold CCV > 0.8 and a ratio CCV/DCV > 5. To identify genes that do not show support for colocalization even with a relaxed threshold, we used a threshold of CCV > 0.5 and CCV/DCV > 2.

To compare the alternative and minor allele frequencies of eSNPs between populations, European genotype data were obtained from the 1000 Genomes dataset.^50^ After subsetting the VCFs for the samples belonging to the European superpopulation, alternative allele counts and frequencies were recalculated with VCFtools.^22^ Minor allele frequencies in Indonesia and Europe were calculated in relation to the minor allele in the Indonesian data.

### Estimating and testing for differences in effect sizes between populations

We used multivariate adaptive shrinkage as implemented in the R package *mashr*^51^ to more reliably estimate effect sizes and to identify shared and population-specific eQTLs. The model was fit with both data-driven and canonical covariances. The data-driven covariance matrix was constructed by identifying strong signals based on a significance threshold of 0.05, by obtaining the initial data-driven covariance matrix for the first two PCs of the strong signals, and then applying the built-in extreme deconvolution algorithm. To facilitate computational limitations, we fit the model by using a random subset of 100,000 SNP-gene pairs. For the calculation of pairwise sharing of eQTLs, an eQTL was considered shared between datasets if the effects are the same sign and within a factor of 0.5.

### Colocalization with blood trait GWAS loci

To connect the QTLs detected here to blood traits, we tested for colocalization between the FDR-significant permutation-based QTLs (FDR-p < 0.1 eQTLs and FDR-p < 0.01 methylQTLs) and 36 hematological traits by using genome-wide summary statistics from Astle et al.^52^ GWAS summary statistics were downloaded from the GWAS catalog.^53^ As no LD information was available, these colocalization analyses were carried out without allowing for multiple causal variants. Similarly, eQTL-GWAS colocalization analysis was carried out with the European datasets.

### Selection scan

We performed a selection scan using a haplotype-based statistic (number of segregating sites by length, nSL),^54^ as implemented in Selscan v1.2.0.^55^ This test identifies ongoing positive selection in the genome by looking for the tracts of extended haplotype homozygosity and is capable of identifying both sweeps from standing variation and incomplete sweeps. To identify the traces of positive selection in three target populations, we used our combined dataset of WGS and imputed genotyping array data represented by approximately 4M SNPs. The following Selscan parameters were used: the maximum allowed gap between loci of 50 kb, the gap scale parameter of 5 kb, and the maximum extent of haplotype homozygosity decay curve of 1,333 loci (approximately 1 Mb given the obtained SNP density). Raw nSL results were normalized with Selscan’s norm package in 50 kb non-overlapping genomic windows with ten allele frequency bins. Windows with less than 21 SNPs were discarded. The proportion of absolute nSL scores > 2 in each 50 kb genomic window was used as a test statistic. Windows with a proportion of SNPs with an absolute nSL > 2 of 30% were considered to be outliers and showing evidence of past positive selection.

To identify regulatory loci under positive selection, we used a colocalization-based method to detect shared signals between the QTL p values and nSL values. We calculated empirical p values for the nSL values by using an outlier approach by sorting all the scores genome-wide and then dividing the rank by the total number of values in the distribution.

### Identifying eQTL effects driven by local ancestry

We calculated the variance explained by modern LA in the genotype of each significant (FDR-p < 0.01) permutation-based eQTL and methylQTL as previously described.^56^ For each eVariant and methylVariant, we fit the linear model V = α × PAP + β, where V is the genotype vector (number of QTL B alleles) and PAP is the LA covariate, representing the number of alleles assigned to the Papuan population. This analysis was carried out with the 73 WGS (30 Mentawai, 29 Sumba, 14 Korowai) samples included in the LA inference. Variants with an absolute R^2^ > 0.7 were considered to exhibit a high correlation with LA. Similarly, we calculated the variance explained by archaic Denisovan and Neanderthal ancestry.

## Results

### Genetic determinants of gene expression and CpG methylation levels in Indonesia

**Figure 1:**
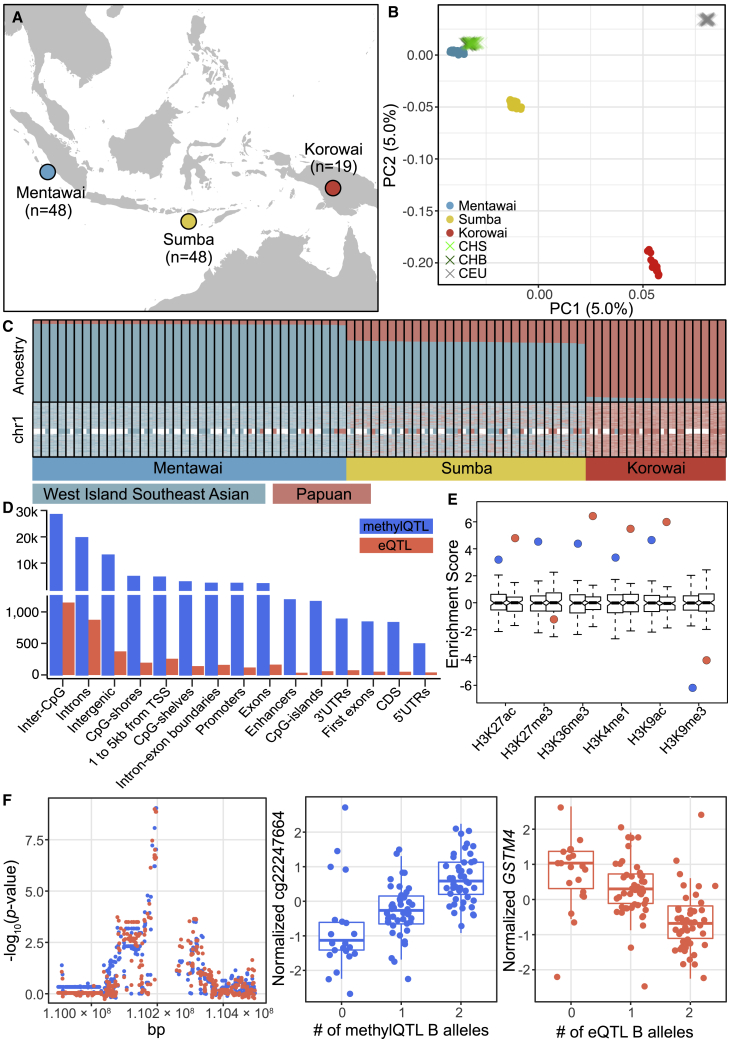
Genetic ancestry and QTL features across 115 Indonesian individuals (A) Map of the sampling locations of the three study populations: Mentawai, blue; Sumba, yellow; Korowai, red. The numbers of samples used in the QTL analyses are indicated. (B) PCA of genotype data from study samples as well as Han Chinese from Beijing (CHB), Southern Han Chinese (CHS), and individuals of European ancestry (CEU) from the 1000 Genomes project. (C) Global and local ancestry across 73 Indonesian individuals (bars) with available WGS data. The top plot shows the average proportion of West Island Southeast Asian and Papuan ancestry genome wide. The bottom plot shows patterns of local ancestry across the two haplotypes in each individual in chr1. (D) Genomic locations of eQTLs (orange) and methylQTLs (blue). Each QTL can have multiple annotations. (E) Enrichment of eQTLs (orange) and methylQTLs (blue) among histone marks derived from primary mononuclear cells from peripheral blood in the Epigenomics Roadmap project compared to a null-distribution of 100 matched random variant sets. (F) An example of a colocalized eQTL-methylQTL pair exhibiting an opposing effect direction on the target trait. The left-side plot shows the −log_10_(p values) of the associations between variants in *cis* and *GSTM4* expression (orange) or cg22247664 methylation (blue). The middle plot shows the relationship between the top-SNP genotype and cg22247664 methylation, and the right-side shows the relationship between the top-SNP genotype and *GSTM4* expression.

To contextualize the genetic diversity in our dataset, we began by clustering the 115 Indonesian samples (Figure 1A) through PCA of genotype data, along with European and Han Chinese samples from the 1000 Genomes project. The first two PCs clearly separate the three study populations (Figure 1B). The Mentawai, of West Island Southeast Asian ancestry, cluster closest to mainland Chinese populations, whereas the Korowai, representative of Papuan ancestry (not well represented in existing public datasets), form a distinct cluster from all other populations. Individuals from Sumba—a mixture of the two ancestries—fall between Mentawai and Korowai, as expected.^8^

We genotyped our samples by using two separate platforms, whole-genome sequencing (WGS, n = 73) and the Illumina Omni 2.5M genotyping array (n = 42; material and methods, Table S1). Using only the complete genome sequences, we inferred patterns of global and local ancestry (LA) and archaic introgression across the three populations. On average, the proportion of the genome for which we can make a confident ancestry assignment is 80% for Mentawai, 71% for Sumba, and 85% for Korowai. The average proportion of ancestry-called individual haploid genomes assigned as Papuan is 5.3% in Mentawai, 26.8% in Sumba, and 95.0% in Korowai (Figure 1C). In addition, we were able to identify Denisovan-introgressed haplotypes covering, on average, 0.13%, 0.48%, and 1.44%, of each haploid genome in Mentawai, Sumba, and Korowai, respectively, consistent with a previous study^6^ showing a high frequency of Denisovan sequence in Korowai (Figure S1). Proportions of inferred Papuan ancestry and Denisovan introgression are highly correlated (Pearson’s r = 0.995, Figure S1). Further, we identified Neanderthal-introgressed haplotypes covering on average 1.08%, 1.19%, and 1.40% of each haploid genome from the three study populations, raising the possibility that either archaic ancestry source has made contributions to gene regulatory architecture in these populations.

To identify genetic variants associated with changes in expression (eQTL) and methylation (methylQTL) levels, we used a linear regression-based approach (material and methods). At an FDR of 0.01, we detect a total of 1,975 significant *cis*-eQTLs (Data S1) and 48,014 *cis*-methylQTLs (Data S2). As expected, the majority of QTLs are located in non-coding parts of the genome, enriched among transcriptionally active histone marks and accessible chromatin, and mostly depleted from marks associated with heterochromatin and repression of transcription across three blood cell lines (Figures 1D and 1E, Figure S2). We then tested for colocalization between 4,639 pairs of CpGs and genes that potentially harbor a common causal variant by using a Bayesian approach (material and methods, supplemental note 1) to better understand how genetic regulation of methylation levels contributes to the regulation of gene expression. Over a wide range of prior probabilities, 720 (15.5%) of the tested pairs show robust support for a common causal variant (material and methods, Figure S3, Data S4), corresponding to 621 unique CpGs and 222 unique genes. As expected, CpGs located on promoters are more likely to show an opposite direction of effect with the gene than CpGs located outside regulatory regions (Fisher’s test p = 3.835 × 10^−6^, Figure 1F, supplemental note 1, Figure S4); additionally, we had previously identified 80 (36.0%) of the 222 genes as showing a negative correlation between expression and promoter methylation levels.^8^

### Population specificity of Indonesian eQTLs

**Figure 2:**
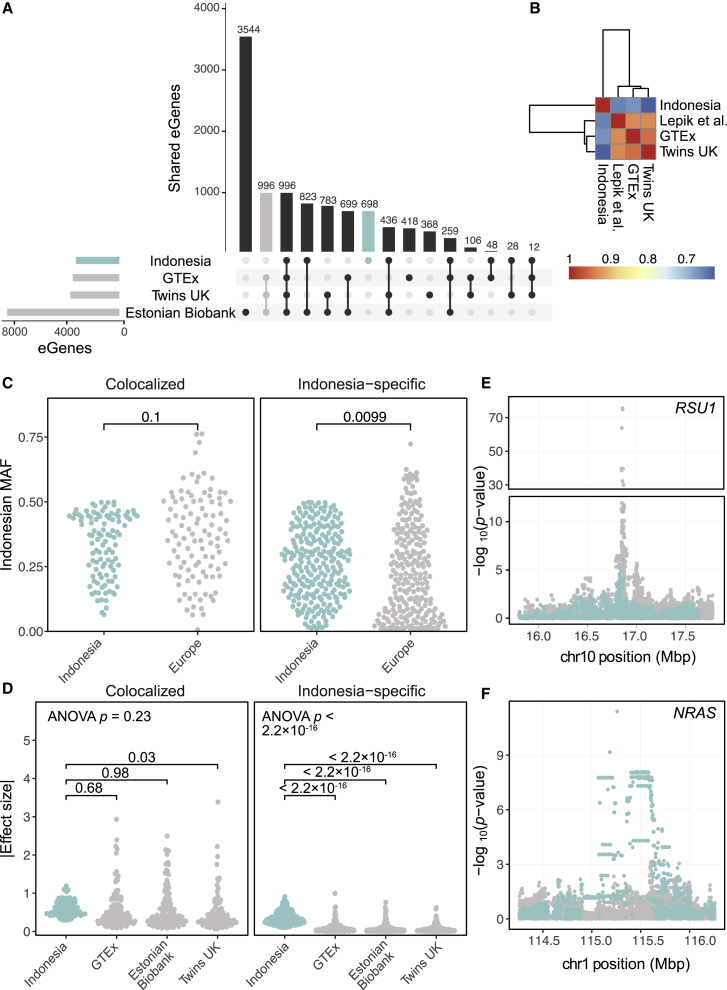
Sharing of eQTLs between Indonesian and European populations (A) Overlap of permutation-significant (FDR-p < 0.1) eGenes between Indonesia and three European eQTL cohorts. (B) Pairwise sharing of eQTLs between the datasets across all tested SNP-gene pairs. (C) Minor allele frequencies of shared and Indonesia-specific eSNPs in Indonesia and the 1000 Genomes European super-population. MAFs are reported relative to the minor allele in Indonesia. (D) Absolute effect sizes of the shared and Indonesia-specific eQTLs. In (C) and (D), ANOVA p values and t test p values between Indonesia and the European datasets are indicated. (E) An example of a colocalized gene, *RSU1*. (F) An example of an Indonesia-specific eQTL for the gene *NRAS*. In (E) and (F), −log_10_(p values) for Indonesia are indicated in blue and for the European datasets are indicated in gray.

The bulk of eQTL studies to date have been carried out in European populations. To better understand the impact of ancestry on the genetic architecture of gene regulation, we compared eQTLs detected here with those identified in three comparable mostly European datasets with publicly available genome-wide summary statistics: GTEx^46^ (n = 670), the Estonian Biobank cohort^47^ (n = 491), and Twins UK^48^ (n = 384). While 996 (9.8%) of the 10,214 unique eGenes were shared across cohorts, the same number of eGenes were detected in all European cohorts but not in Indonesia (Figure 2A). Furthermore, 698 (6.8%) were only detected in the Indonesian data. After relaxing our FDR threshold to p < 0.10 to account for differences in power, we tested 3,300 Indonesian eGenes for eQTL colocalization with any of the European datasets. On average, 26.9% of tested genes showed some evidence of colocalization and 6.71% showed robust support across a wide range of prior probabilities with each European dataset (Table S4). In total, 1,177 (35.7%) of tested genes showed some evidence of colocalization and 318 (9.6%) robust support for colocalization with a wide range of prior probabilities for a common causal variant with at least one European dataset. Of these, 105 (33.0%) genes (Table S4), including Ras suppressor *RSU1* (Figure 2E, Figure S5), showed robust support for colocalization between Indonesia and all European datasets. In contrast, we found much higher levels of colocalization when comparing between European datasets at a similarly relaxed FDR: on average, 46.3% of tested genes colocalized across at least one pair of datasets with some support and 17.8% with robust support (Table S4). These results suggest a true difference in eQTL architecture between the Indonesian and European data. Indeed, 1,081 (32.8%) of genes exhibited no evidence of colocalization between Indonesia and any European dataset, even with relaxed thresholds (material and methods). We followed up on these results by using a multivariate adaptive shrinkage model (material and methods) that enables joint analysis of all the datasets simultaneously and is geared toward more accurately estimating effect sizes and identifying shared and specific effects. Concordant with the colocalization results above, we found eQTL effects were largely shared between the European datasets (Figure 2B). We also identified 2,411 Indonesia-specific eSNP-eGene pairs, corresponding to a total of 1,599 eGenes. To generate a robust set of Indonesia-specific eQTLs, we intersected the results from the colocalization and multivariate analyses and identified 245 genes that harbored Indonesia-specific eQTLs and had no evidence of colocalization with European eQTLs. It should be noted that these Indonesia-specific eQTLs are likely to be present in some unsurveyed populations and thus specific to particular populations and not only Indonesia. As our comparison using the available datasets is between European and Indonesian eQTLs, we are calling this set of eQTLs Indonesia specific in the current study.

To identify the attributes of Indonesia-specific eQTLs, we compared these 245 genes to the set of 105 genes with robust support for colocalization between Indonesia and all European datasets. Although there is no overall enrichment for GO or KEGG terms among Indonesian-specific eGenes, there are clinically relevant genes in this set (Table S5), including the *NRAS* proto-oncogene (Figure 2F, Figure S5). Concordant with previous reports, eQTLs that are shared between populations exhibit larger effect sizes than other eQTLs (t test p < 2.2 × 10^−16^), and most (97%–100%) shared eQTLs show the same direction of effect in both populations^57^ (Figure S6). Indonesia-specific eQTLs exhibit significantly larger effect sizes in Indonesia than in the European datasets, while colocalized eQTLs show no statistically significant differences in effect sizes between datasets, as expected (Figure 2D). We hypothesized that differences in genetic trait architecture may underlie these population-specific eQTLs. Indeed, when we compared the MAF of eSNPs between Indonesians and Europeans, we found no significant difference among the colocalized eQTLs but a significantly higher MAF in Indonesians for the Indonesian-specific eQTLs (t test; mean MAF in Indonesia 0.26; in the 1000 Genomes European super population 0.22; p = 0.0099), suggesting population differences in haplotype structure contribute to our observation. However, they alone were insufficient to explain the entirety of Indonesia-specific eQTLs. Thus, we also examined the role of gene expression levels—i.e., whether a gene is highly expressed in Indonesian samples but expressed at low levels or not at all in European ones. Indeed, as with MAF, we observed no significant difference in expression levels of colocalized eGenes across the datasets, but there was a significant increase in expression of the Indonesia-specific eGenes (t test; mean log_2_(TPM) in Indonesia 3.5, in GTEx whole blood 2.6, p < 2.8 × 10^−12^, Figure S7). Interestingly, there were a number of Indonesia-specific eQTLs where little or no difference in MAF or gene expression was observed, highlighting the need for a deeper mechanistic investigation of these loci (Figure S8).

While some of the population specificity we detect may be attributable to limited statistical power across all datasets, our findings illustrate the value of performing analyses on diverse populations to achieve a comprehensive understanding of the genetic regulation of molecular traits. Importantly, of all datasets under consideration, the Indonesian one is the smallest, making our inability to replicate Indonesian eQTLs in European studies more likely to be biologically meaningful and clear candidates for functional follow-up.

### Subsets of expression and methylation QTLs are largely driven by modern local ancestry and archaic introgression

**Figure 3:**
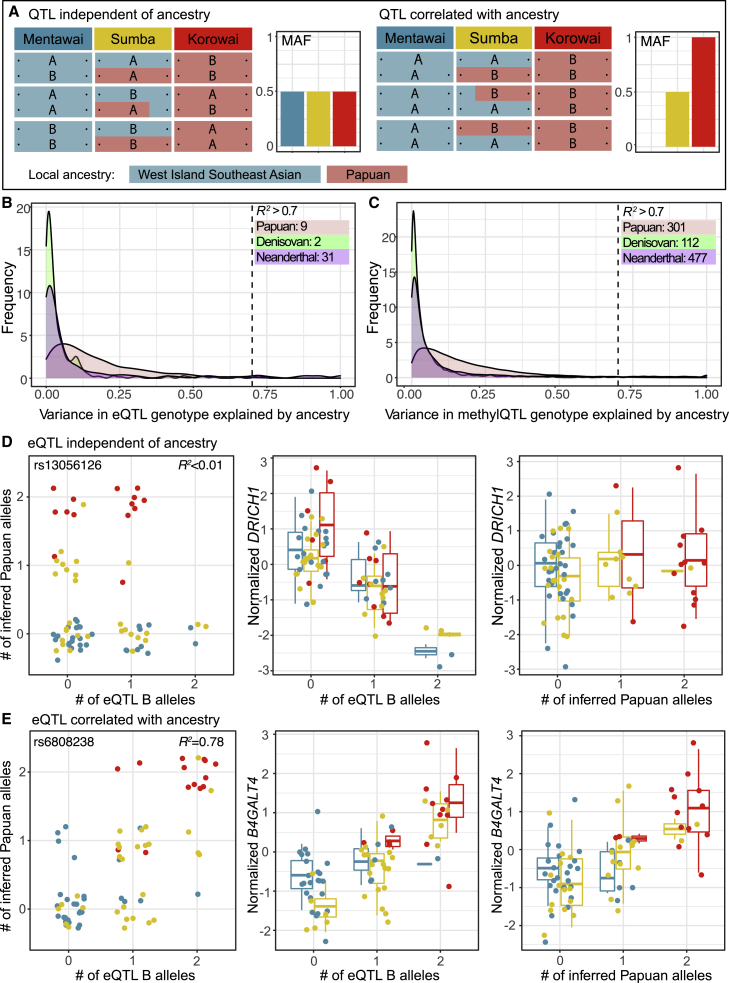
Integrating local ancestry inference at regulatory loci to detect QTLs driven by ancestry and archaic introgression (A) Schematic illustrations of variation in QTL genotype (A = major allele, B = minor allele) and local ancestry are shown across the two haplotypes in three individuals in three populations. In the first example, QTL genotype variation is independent of local ancestry and allele frequencies are equal between populations. In the second example, QTL B allele closely segregates with the ancestry informative marker and allele frequencies differ between populations. There is an expected correlation between the genotype and the molecular trait, as well as inferred ancestry and the trait. (B and C) Linear regression between the numbers of QTL B alleles and numbers of inferred Papuan, Denisovan, and Neanderthal alleles reveal subsets of (B) eQTLs and (C) methylQTLs largely driven by modern LA and archaic introgression. The numbers of QTLs exceeding the *R*^*2*^ threshold of 0.7 are indicated. (D) An example of an eQTL independent of modern LA. (E) An example of an eQTL highly correlated with modern LA. In (D) and (E), the leftmost plot shows the correlation between the number of inferred Papuan alleles and eQTL B alleles. rs ID and *R*^*2*^ are indicated. The middle plot shows the effect of the eQTL B allele dosage on the normalized expression level of the target gene. The rightmost plot shows the effect of the inferred Papuan allele dosage on the target gene.

In addition to differences between Indonesians and Europeans, we sought to understand the extent to which the two distinct sources of LA in modern Indonesians, as well as introgression from archaic hominins, have impacted gene regulatory architecture. We examined the haplotype background on which our QTLs occur and asked whether there was a relationship between the inferred ancestral source of the genotype and expression/methylation levels (material and methods, Figures 3A, 3D, and 3E). We find nine, two, and 31 instances where variance in eQTL genotype is largely driven (*R*^*2*^ > 0.7) by modern LA, archaic Denisovan introgression, and archaic Neanderthal introgression, respectively (Figure 3B, Data S5, Data S6, Data S7), directly linking ancestry-informative alleles to expression differences between individuals. Similarly, we find 301, 112, and 477 instances where the methylQTL genotype is driven by modern LA, Denisovan introgression, and Neanderthal introgression, respectively (Figure 3C, Data S8, Data S9, Data S10). In total, 2.1% of eQTLs and 2.29% of methylQTLs are driven by modern LA or archaic introgression; given the unbalanced representation of the two ancestries in our dataset (Figure S1) this number represents only a lower boundary, and the true number is likely to be much greater.

Of the nine and 373 unique Papuan-driven QTL target genes and CpGs, we had previously identified seven (77.8%) and 270 (72.4%) as differentially expressed/methylated in at least one of the pairwise comparisons between the three study populations.^8^ Further, 42 out of the 122 (34.4%) Denisovan-driven methylQTL targets were differentially methylated and seven (22.6%) and 149 (25.6%) of the Neanderthal-driven eQTL and methylQTL targets are differentially expressed/methylated. However, despite multiple lines of evidence suggesting that some introgressed Neanderthal and Denisovan alleles have been positively selected for in human populations,^58^, ^59^, ^60^ we were unable to identify evidence of recent positive selection among these loci (supplemental note 3, Table S6, Table S7). Altogether, our findings highlight the potential for local sources of genetic ancestry, whether modern or archaic, to variably impact gene expression architecture across populations and again emphasize the importance of truly diverse sampling.

### Connecting regulatory variants to complex traits

Differences in genetic architecture between populations, including differences in allele frequencies and patterns of LD, are known to limit the transferability of GWASs and polygenic risk scores across populations.^1^^,^^4^ Thus, we sought to examine how the differences in QTL architecture between Indonesian and European populations propagate through to the genetic underpinnings of complex traits.

First, using the same Bayesian methodology as above, we tested for colocalization between the significant Indonesian QTLs and 36 hematological traits by using genome-wide summary statistics from a GWAS on 173,480 participants of European ancestry.^52^ We detected 30 (1.5%) and 614 (1.3%) unique eGenes and methylCpGs, respectively, that colocalize with at least one trait (Table S8); in total, we identified 78 significant trait-eGene pairs. The genes and CpGs colocalized with the most unique traits were *ITGA4* and cg18815117, colocalizing with ten and 16 traits, respectively. *ITGA4* has been previously implicated in blood trait GWASs across diverse populations.^61^, ^62^, ^63^, ^64^, ^65^ The CpG cg18815117 is located in the body of *CRHR1*, an important regulator of the hypothalamic–pituitary–adrenal (HPA) axis. Epigenetic changes in the body and promoter of *CRHR1* have been found to be highly predictive of major depressive disorder and panic disorder in some cohorts.^66^, ^67^, ^68^

**Figure 4:**
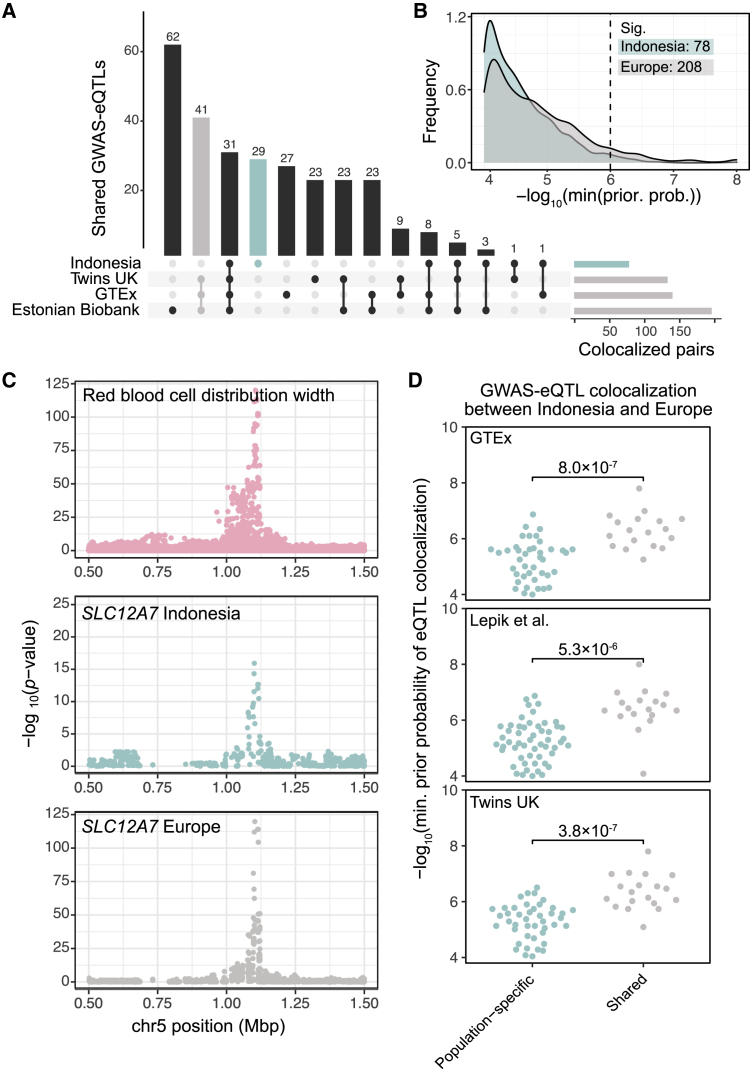
GWAS colocalization with eQTLs from diverse populations identifies shared and population-specific variant-gene-trait associations (A) Overlap of colocalized trait-gene pairs. The horizontal bar plot shows the numbers of significant colocalized pairs for each dataset. The dot plot shows the intersections and the vertical bar plot shows the numbers of shared trait-gene pairs for each intersection. (B) European GWAS shows more colocalization with European eQTLs than Indonesian eQTLs. The x axis shows the −log_10_ of the lower bound of the prior probability of colocalization where the gene passes the colocalization threshold, and larger values indicate a more robust support for eQTL colocalization. The minimum prior probability threshold of 1.0 × 10^−6^ for robust colocalization is indicated. (C) An example of a GWAS-eQTL significantly colocalized across Indonesia and all European datasets. −log_10_(p values) for red blood cell component distribution width (top), *SLC12A7* eQTLs in Indonesia (middle), and *SLC12A7* eQTLs in the three European datasets (bottom) are shown. (D) Population-specific GWAS-colocalized eGenes are less likely to show eQTL colocalization between Indonesia and Europe than shared GWAS-genes. The y axis shows the −log_10_ of the lower bound of the prior probability of colocalization where the gene passes the colocalization threshold, and larger values indicate more robust support for eQTL colocalization.

Next, we repeated the GWAS colocalization analysis, this time by using the three European eQTL datasets alone. Compared to the 30 eGenes that colocalized with at least one GWAS trait, here we found an average of 55 unique eGenes colocalizing with at least one trait (45 in GTEx, 48 in TwinsUK, and 77 in the Estonian Biobank). Focusing on pairs of colocalized GWAS-eQTLs—rather than unique eGenes—we found that 72 (28%) of the 257 unique significant trait-gene pairs detected across all datasets were shared across all three European datasets (Figure 4A) and an average of 25.7% of colocalized genes were shared between datasets for a given trait (Table S9). Furthermore, 31 GWAS-eQTL pairs (12%) were shared between all three European datasets and Indonesia. The GWAS-eQTLs shared between Indonesia and Europe show robust support for eQTL colocalization, indicative of a shared genetic architecture underlying the regulation of these genes and the biological association identified by GWAS (Figure 4B). Among the shared GWAS-eQTLs, we identify *SLC12A7*, which colocalized with red blood cell distribution width, a biomarker representing the variability in the size of circulating erythrocytes (Figure 4C). Variants annotated with *SLC12A7* have previously been uncovered in GWASs on this and other datasets.^52^^,^^69^^,^^70^

In addition to these population-shared GWAS-eQTLs, we also identified population-specific colocalized pairs of GWAS-eQTLs. Overall, European eQTLs show more evidence for colocalization with GWAS traits than Indonesian eQTLs (Figures 4A and 4B). Indeed, 208 (80.9%) of the 257 unique eGene-trait pairs that were detected across at least one of the European datasets could not be replicated in Indonesia. Intriguingly, although the GWAS was performed in a predominantly European sample, 29 GWAS-eQTL pairs colocalized exclusively in the Indonesian data and not in any of the European datasets (Figure 4A). Out of these, eight were not colocalized in any of the European datasets even with a relaxed significance threshold (see material and methods). Reassuringly, these population-specific GWAS genes show low support for eQTL colocalization (Figure 4D), again strongly arguing for differences in the genetic architecture underlying gene regulation, while simultaneously supporting the importance of the genes in question in contributing to the overall trait. The striking difference in GWAS colocalization between European and Indonesian eQTLs reflects the poor transferability of genetic association studies between populations.

Finally, we sought to identify local ancestry or archaic introgression-driven QTLs that are associated with hematological traits. Among the GWAS-colocalizing QTLs were a Papuan-driven methylQTL that colocalizes with hemoglobin measurements, and notably, four Denisovan-associated methylQTLs that colocalize with platelet count. We further examined these four methylQTLs to gain insight into possible mechanisms underlying the connection with platelet count (supplemental note 4, Table S10). All four target CpGs are located near the *HLA* superlocus. While these methylQTLs do not significantly colocalize with eQTLs in our data, all four methylSNPs are nominally associated with the expression of the nearby gene *ZFP57* (lowest p value = 5.85 × 10^−6^). *ZFP57* is a transcriptional regulator known to have an important role in DNA methylation, epigenetic regulation, and imprinting during development.^71^ Expression of *ZFP57* is dependent on underlying genetic variation, and while the biology of *ZFP57* in adults is not well studied, it has been implicated as the causal gene connecting some GWAS variants to cancer and HIV/AIDS progression.^72^ As above, the geographically restricted ancestry of the methylSNPs and their linkage structure suggests that the regulatory interactions may not be fully shared between populations. Further GWASs and functional studies on diverse populations are needed for the fine-mapping of causal variants underlying gene regulation and complex traits.

## Discussion

Indonesia is the world’s fourth most populous country and a region that has been vastly understudied, but it is also one that is undergoing a rapid demographic and lifestyle shift giving rise to an expanding middle class and where non-infectious, complex diseases are already contributing substantially to mortality and morbidity. As is happening elsewhere in the Global South, this transition accelerates the need to understand the molecular underpinnings of complex disease, and in this context, our study adds to a growing literature demonstrating the importance of characterizing functional genomics within traditionally understudied populations.^73^^,^^74^

We have explored the degree to which functional variation differs between Europeans and Indonesians, and more broadly, the difficult problem of translating eQTL knowledge across populations. Focusing largely on a set of eQTLs that had strong evidence of being colocalized between populations and a set of eQTLs with strong evidence of being specific to Indonesia, we were able to examine potential drivers of population specificity. At least some portion of the population-specific effects we observe are explained by population-specific genomic architecture at *cis*-regulatory regions. The future identification of such eQTLs is fully contingent on performing large-scale QTL studies in underrepresented populations, and our work demonstrates the value of such approaches. Other drivers of population differences in architecture are likely to include *trans* effects (themselves most likely driven by population-specific *cis* effects),^75^ as well as environmental differences between populations.^76^ The identification of 245 putative population-specific eQTLs—a number of which implicate genes involved with biological processes such as immunity and cancer progression—coupled with the insights gained from exploring the genetic underpinnings of these eQTLs advances our understanding of the genetic architecture of gene regulation. In the future, the collection of multimodal data and mapping of QTLs across tissues and diverse populations can allow a more comprehensive assessment of population specificity and the exact mechanisms underlying population differences in gene regulation. In particular, future surveys across South East Asia can further clarify the genomic and environmental drivers of gene regulatory variation in the area.

In addition to comparisons between Indonesian and European populations, we were able to leverage the unique cline of Asian and Papuan ancestry in Indonesia to identify both QTLs driven by local ancestry or introgression from archaic hominin species. A large proportion of the target genes and CpGs of these LA-driven QTLs were previously identified as differentially expressed or methylated between islands, demonstrating how between-island differences in genetic ancestry can contribute to differences in molecular phenotypes in the region. Notably, these loci, which might *a priori* appear to be prime candidates for local adaptation, showed no evidence of having been targets of positive selection. This suggests that the relationship between trait architecture, demographic history, and adaptation to local pressures such as pathogens is not straightforward. However, our analysis demands a strong correlation between allelic state and ancestry, making our estimates of LA-driven QTLs highly conservative and identifies loci that have evolved under specific scenarios, e.g., drift to high frequency in the Papuan population. Relaxing these demands may lead to the discovery of other regulatory variants that arose within Papua after divergence from Asia. Furthermore, our analysis is limited by the small sample size. Future sample collection efforts across diverse populations will address this limitation. Understanding how complex gene regulatory landscapes and the polygenicity of most traits constrain the action of natural selection is an open challenge in human genomics, one underscored by the difficulty many studies of genome-wide positive selection in humans have encountered in linking evidence of selection at the DNA level to tangible phenotypes.^77^^,^^78^

Similarly, the modest overlap of GWAS hits with the population-specific QTLs represents a non-trivial challenge in the field of functional genomics: how do we connect population-specific functional variation to loci associated with complex traits identified in European populations? From a practical perspective, we do not anticipate a robust expansion of traditional GWASs’ being carried out in understudied populations. To this end, the field will need to move away from simple intersections of GWASs and QTL hits, which rely upon shared LD structure, and instead integrate genetic variation, GWAS results, context-specific multi-omics (in simulated or actual disease states and in a range of cell types), and robust functional validations to define common sets of regulatory elements that contribute to disease and are shared across populations.

## Data and code availability

All genotype data, RNA sequencing reads, and Illumina Epic iDat files are available through the Data Access Committee of the official data repository at the European Genome-phenome Archive (EGA; web resources). Illumina Omni 2.5M genotyping array data are deposited in study EGA: EGAS00001003670 and the whole-genome data are deposited in study EGA: EGAS00001003654. The RNA sequencing data are deposited in study EGA: EGAS00001003671 and the methylation data are deposited in study EGA: EGAS00001003653. Matrices of unfiltered read counts (https://doi.org/10.26188/5d12023f77da8) and M-values (https://doi.org/10.26188/5d13fb401e305) for all samples, including replicates, are freely available on Figshare (web resources). The nominal eQTL statistics for all tested SNP-gene pairs are available as Data S3 and the nominal methylQTL statistics for all SNPs tested for the targets of the permutation-significant methylQTLs are available at Mendeley Data: https://doi.org/10.17632/z7rgf54jd6.2. The code generated during this study is available on GitHub (web resources).

## Web resources

ADMIXTURE v1.3, https://dalexander.github.io/admixture/
AnnotationHub v2.16.1, https://bioconductor.org/packages/release/bioc/html/AnnotationHub.html
annotatr v1.10.0, https://bioconductor.org/packages/release/bioc/html/annotatr.html
bcftools v1.9, https://samtools.github.io/bcftools/
Beagle v5.1, https://faculty.washington.edu/browning/beagle/beagle.html
BLAST, https://blast.ncbi.nlm.nih.gov/Blast.cgi?PAGE_TYPE=BlastSearch
Broad Institute genome annotations, http://software.broadinstitute.org/software/genomestrip/node_ReferenceMetadata.html
ChromoPainter v2, https://people.maths.bris.ac.uk/∼madjl/finestructure-old/chromopainter_info.html
clusterProfiler v3.14.3, https://bioconductor.org/packages/release/bioc/html/clusterProfiler.html
coloc v4, https://chr1swallace.github.io/coloc/
Eagle v2.4, https://alkesgroup.broadinstitute.org/Eagle/
ENCODE portal, https://www.encodeproject.org/
eQTL catalog, https://www.ebi.ac.uk/eqtl/
European Genome-Phenome Archive, https://ega-archive.org/
FANTOM5, https://fantom.gsc.riken.jp/data/
FastQC v0.11.5, https://www.bioinformatics.babraham.ac.uk/projects/fastqc/
featureCounts v1.5.3, http://subread.sourceforge.net/
FigShare, https://figshare.com
GATK4, https://gatk.broadinstitute.org/hc
GENCODE release 27, https://www.gencodegenes.org/human/release_27.html
GitHub repository for this publication, https://github.com/heinin/Indonesia_QTL
GRCh37 human reference sequence (hs37d5), ftp://ftp.1000genomes.ebi.ac.uk/vol1/ftp/technical/reference/phase2_reference_assembly_sequence
GRCh38 Ensembl release 90 August 2017, ftp://ftp.ensembl.org/pub/release-90/gtf/homo_sapiens/Homo_sapiens.GRCh38.90.chr.gtf.gz
GWAS catalog, https://www.ebi.ac.uk/gwas/
liftOver v1.8.0, https://www.bioconductor.org/packages/release/workflows/html/liftOver.html
lumi v2.36.0, https://www.bioconductor.org/packages/release/bioc/html/lumi.html
mashr v0.2.50, https://cran.r-project.org/web/packages/mashr/index.html
MatchIt v3.0.2, https://cran.r-project.org/web/packages/MatchIt/
minfi v1.30.0, https://bioconductor.org/packages/release/bioc/html/minfi.html
NIH Epigenomics Roadmap FTP, ftp: //ftp.ncbi.nlm.nih.gov/pub/geo/DATA/roadmapepigenomics/
PEER v1.3, https://github.com/PMBio/peer
PLINK v1.9, https://www.cog-genomics.org/plink/
powerEQTL v0.1.7, https://cran.r-project.org/web/packages/powerEQTL/index.html
QTLtools v1.0, https://qtltools.github.io/qtltools/
Selscan v1.2.0, https://github.com/szpiech/selscan
SNPRelate v1.18.1, https://www.bioconductor.org/packages/release/bioc/html/SNPRelate.html
STAR v2.5.3a, https://github.com/alexdobin/STAR
The Polaris Project, https://github.com/Illumina/Polaris
Trimmomatic v0.36, http://www.usadellab.org/cms/?page=trimmomatic
TxDb.Hsapiens.UCSC.hg19.knownGene v3.2.2, https://bioconductor.org/packages/release/data/annotation/html/TxDb.Hsapiens.UCSC.hg19.knownGene.html
UCSC genome annotations, http://hgdownload.soe.ucsc.edu/goldenPath/hg19/database/genomicSuperDups.txt.gz
Uniquely mappable regions with various kmers, https://bismap.hoffmanlab.org/
VCFtools v0.1.13, https://vcftools.github.io/index.html
VSE v0.99, https://cran.r-project.org/src/contrib/Archive/VSE/

## Supplemental information

Document S1. Figures S1–S13 and supplemental notes 1–4

Data S1. Permutation-significant eQTLs

Data S2. Permutation-significant methylQTLs

Data S3. Nominal eQTL statistics

Data S4. eQTL-methylQTL colocalization results for robust colocalized pairs

Data S5. eQTL-LAI correlation results for Papuan ancestry

Data S6. eQTL-LAI correlation results for Denisovan introgression

Data S7. eQTL-LAI correlation results for Neanderthal introgression

Data S8. methylQTL-LAI correlation results for Papuan ancestry

Data S9. **methylQTL-LAI correlation results for Denisovan introgression**

Data S10. **methylQTL-LAI correlation results for Neanderthal introgression**

Table S1. Sampling locations and batch information

Table S2. RNA sequencing sample information

Table S3. The 61 sub-Saharan African samples used in archaic introgression inference

Table S4. Numbers of colocalized and non-colocalized genes in each pairwise comparison between Indonesia and the European cohorts

Table S5. Descriptions of the target genes of Indonesian eQTLs that show no evidence of colocalization with any of the tested European datasets and harbored an Indonesia-specific eQTL according to the *mashr* analysis

Table S6. p values for overrepresentation (Fisher’s test) of modern and archaic LAI-driven QTLs among the genomic windows under positive selection in each population

Table S7. Ancestry-driven QTLs under positive selection

Table S8. Summary of Indonesian eQTLs and methylQTLs that colocalize with each of the 36 hematological GWAS traits reported in Astle et al. (2016) ^52^

Table S9. Summary of European eQTLs that colocalize with each of the 36 hematological GWAS traits reported in Astle et al. (2016) ^52^

Table S10. Qualities of the Denisovan-driven methylQTLs that colocalize with platelet count GWAS

Document S2. Article plus supplemental information

## References

[1] Duncan L., Shen H., Gelaye B., Meijsen J., Ressler K., Feldman M., Peterson R., Domingue B. (2019). Analysis of polygenic risk score usage and performance in diverse human populations. Nat. Commun..

[2] Landry L.G., Ali N., Williams D.R., Rehm H.L., Bonham V.L. (2018). Lack Of Diversity In Genomic Databases Is A Barrier To Translating Precision Medicine Research Into Practice. Health Aff. (Millwood).

[3] Sirugo G., Williams S.M., Tishkoff S.A. (2019). The Missing Diversity in Human Genetic Studies. Cell.

[4] Durvasula A., Lohmueller K.E. (2021). Negative selection on complex traits limits phenotype prediction accuracy between populations. Am. J. Hum. Genet..

[5] Hudjashov G., Karafet T.M., Lawson D.J., Downey S., Savina O., Sudoyo H., Lansing J.S., Hammer M.F., Cox M.P. (2017). Complex Patterns of Admixture across the Indonesian Archipelago. Mol. Biol. Evol..

[6] Jacobs G.S., Hudjashov G., Saag L., Kusuma P., Darusallam C.C., Lawson D.J., Mondal M., Pagani L., Ricaut F.-X., Stoneking M. (2019). Multiple Deeply Divergent Denisovan Ancestries in Papuans. Cell.

[7] Quintana-Murci L. (2019). Human Immunology through the Lens of Evolutionary Genetics. Cell.

[8] Natri H.M., Bobowik K.S., Kusuma P., Crenna Darusallam C., Jacobs G.S., Hudjashov G., Lansing J.S., Sudoyo H., Banovich N.E., Cox M.P., Gallego Romero I. (2020). Genome-wide DNA methylation and gene expression patterns reflect genetic ancestry and environmental differences across the Indonesian archipelago. PLoS Genet..

[9] University of Tartu (2018). https://neic.no/utrocket/.

[10] Van der Auwera G.A., Carneiro M.O., Hartl C., Poplin R., Del Angel G., Levy-Moonshine A., Jordan T., Shakir K., Roazen D., Thibault J. (2013). From FastQ data to high confidence variant calls: the Genome Analysis Toolkit best practices pipeline. Curr. Protoc. Bioinformatics.

[11] Poplin R., Ruano-Rubio V., DePristo M.A., Fennell T.J., Carneiro M.O., Van der Auwera G.A., Kling D.E., Gauthier L.D., Levy-Moonshine A., Roazen D. (2018). Scaling accurate genetic variant discovery to tens of thousands of samples. bioRxiv.

[12] Mallick S., Li H., Lipson M., Mathieson I., Gymrek M., Racimo F., Zhao M., Chennagiri N., Nordenfelt S., Tandon A. (2016). The Simons Genome Diversity Project: 300 genomes from 142 diverse populations. Nature.

[13] Malaspinas A.-S., Westaway M.C., Muller C., Sousa V.C., Lao O., Alves I., Bergström A., Athanasiadis G., Cheng J.Y., Crawford J.E. (2016). A genomic history of Aboriginal Australia. Nature.

[14] Vernot B., Tucci S., Kelso J., Schraiber J.G., Wolf A.B., Gittelman R.M., Dannemann M., Grote S., McCoy R.C., Norton H. (2016). Excavating Neandertal and Denisovan DNA from the genomes of Melanesian individuals. Science.

[15] Lan T., Lin H., Zhu W., Laurent T.C.A.M., Yang M., Liu X., Wang J., Wang J., Yang H., Xu X., Guo X. (2017). Deep whole-genome sequencing of 90 Han Chinese genomes. Gigascience.

[16] Li H. (2011). A statistical framework for SNP calling, mutation discovery, association mapping and population genetical parameter estimation from sequencing data. Bioinformatics.

[17] Meyer M., Kircher M., Gansauge M.-T., Li H., Racimo F., Mallick S., Schraiber J.G., Jay F., Prüfer K., de Filippo C. (2012). A high-coverage genome sequence from an archaic Denisovan individual. Science.

[18] Prüfer K., Racimo F., Patterson N., Jay F., Sankararaman S., Sawyer S., Heinze A., Renaud G., Sudmant P.H., de Filippo C. (2014). The complete genome sequence of a Neanderthal from the Altai Mountains. Nature.

[19] Loh P.-R., Palamara P.F., Price A.L. (2016). Fast and accurate long-range phasing in a UK Biobank cohort. Nat. Genet..

[20] Chang C.C., Chow C.C., Tellier L.C., Vattikuti S., Purcell S.M., Lee J.J. (2015). Second-generation PLINK: rising to the challenge of larger and richer datasets. Gigascience.

[21] Browning B.L., Zhou Y., Browning S.R. (2018). A One-Penny Imputed Genome from Next-Generation Reference Panels. Am. J. Hum. Genet..

[22] Danecek P., Auton A., Abecasis G., Albers C.A., Banks E., DePristo M.A., Handsaker R.E., Lunter G., Marth G.T., Sherry S.T. (2011). The variant call format and VCFtools. Bioinformatics.

[23] Bolger A.M., Lohse M., Usadel B. (2014). Trimmomatic: a flexible trimmer for Illumina sequence data. Bioinformatics.

[24] Dobin A., Davis C.A., Schlesinger F., Drenkow J., Zaleski C., Jha S., Batut P., Chaisson M., Gingeras T.R. (2013). STAR: ultrafast universal RNA-seq aligner. Bioinformatics.

[25] Liao Y., Smyth G.K., Shi W. (2014). featureCounts: an efficient general purpose program for assigning sequence reads to genomic features. Bioinformatics.

[26] Frankish A., Diekhans M., Ferreira A.-M., Johnson R., Jungreis I., Loveland J., Mudge J.M., Sisu C., Wright J., Armstrong J. (2019). GENCODE reference annotation for the human and mouse genomes. Nucleic Acids Res..

[27] Aryee M.J., Jaffe A.E., Corrada-Bravo H., Ladd-Acosta C., Feinberg A.P., Hansen K.D., Irizarry R.A. (2014). Minfi: a flexible and comprehensive Bioconductor package for the analysis of Infinium DNA methylation microarrays. Bioinformatics.

[28] Maksimovic J., Gordon L., Oshlack A. (2012). SWAN: Subset-quantile within array normalization for illumina infinium HumanMethylation450 BeadChips. Genome Biol..

[29] Du P., Kibbe W.A., Lin S.M. (2008). lumi: a pipeline for processing Illumina microarray. Bioinformatics.

[30] Lawson D.J., Hellenthal G., Myers S., Falush D. (2012). Inference of population structure using dense haplotype data. PLoS Genet..

[31] Alexander D.H., Novembre J., Lange K. (2009). Fast model-based estimation of ancestry in unrelated individuals. Genome Res..

[32] Skov L., Hui R., Shchur V., Hobolth A., Scally A., Schierup M.H., Durbin R. (2018). Detecting archaic introgression using an unadmixed outgroup. PLoS Genet..

[33] Zheng X., Levine D., Shen J., Gogarten S.M., Laurie C., Weir B.S. (2012). A high-performance computing toolset for relatedness and principal component analysis of SNP data. Bioinformatics.

[34] Stegle O., Parts L., Piipari M., Winn J., Durbin R. (2012). Using probabilistic estimation of expression residuals (PEER) to obtain increased power and interpretability of gene expression analyses. Nat. Protoc..

[35] Stegle O., Parts L., Durbin R., Winn J. (2010). A Bayesian framework to account for complex non-genetic factors in gene expression levels greatly increases power in eQTL studies. PLoS Comput. Biol..

[36] Delaneau O., Ongen H., Brown A.A., Fort A., Panousis N.I., Dermitzakis E.T. (2017). A complete tool set for molecular QTL discovery and analysis. Nat. Commun..

[37] Dong X., Li X., Chang T.-W., Scherzer C.R., Weiss S.T., Qiu W. (2021). powerEQTL: An R package and shiny application for sample size and power calculation of bulk tissue and single-cell eQTL analysis. Bioinformatics.

[38] Cavalcante R.G., Sartor M.A. (2017). annotatr: genomic regions in context. Bioinformatics.

[39] Andersson R., Gebhard C., Miguel-Escalada I., Hoof I., Bornholdt J., Boyd M., Chen Y., Zhao X., Schmidl C., Suzuki T. (2014). An atlas of active enhancers across human cell types and tissues. Nature.

[40] Ahmed M., Sallari R.C., Guo H., Moore J.H., He H.H., Lupien M. (2017). Variant Set Enrichment: an R package to identify disease-associated functional genomic regions. BioData Min..

[41] Chadwick L.H. (2012). The NIH Roadmap Epigenomics Program data resource. Epigenomics.

[42] Davis C.A., Hitz B.C., Sloan C.A., Chan E.T., Davidson J.M., Gabdank I., Hilton J.A., Jain K., Baymuradov U.K., Narayanan A.K. (2018). The Encyclopedia of DNA elements (ENCODE): data portal update. Nucleic Acids Res..

[43] Yu G., Wang L.-G., Han Y., He Q.-Y. (2012). clusterProfiler: an R package for comparing biological themes among gene clusters. OMICS.

[44] Giambartolomei C., Vukcevic D., Schadt E.E., Franke L., Hingorani A.D., Wallace C., Plagnol V. (2014). Bayesian test for colocalisation between pairs of genetic association studies using summary statistics. PLoS Genet..

[45] Wallace C. (2020). Eliciting priors and relaxing the single causal variant assumption in colocalisation analyses. PLoS Genet..

[46] GTEx Consortium (2020). The GTEx Consortium atlas of genetic regulatory effects across human tissues. Science.

[47] Lepik K., Annilo T., Kukuškina V., Kisand K., Kutalik Z., Peterson P., Peterson H., eQTLGen Consortium (2017). C-reactive protein upregulates the whole blood expression of CD59 - an integrative analysis. PLoS Comput. Biol..

[48] Buil A., Brown A.A., Lappalainen T., Viñuela A., Davies M.N., Zheng H.-F., Richards J.B., Glass D., Small K.S., Durbin R. (2015). Gene-gene and gene-environment interactions detected by transcriptome sequence analysis in twins. Nat. Genet..

[49] Kerimov N., Hayhurst J.D., Peikova K., Manning J.R., Walter P., Kolberg L., Samoviča M., Sakthivel M.P., Kuzmin I., Trevanion S.J. (2021). A compendium of uniformly processed human gene expression and splicing quantitative trait loci. Nat. Genet..

[50] Auton A., Brooks L.D., Durbin R.M., Garrison E.P., Kang H.M., Korbel J.O., Marchini J.L., McCarthy S., McVean G.A., Abecasis G.R., 1000 Genomes Project Consortium (2015). A global reference for human genetic variation. Nature.

[51] Urbut S.M., Wang G., Carbonetto P., Stephens M. (2019). Flexible statistical methods for estimating and testing effects in genomic studies with multiple conditions. Nat. Genet..

[52] Astle W.J., Elding H., Jiang T., Allen D., Ruklisa D., Mann A.L., Mead D., Bouman H., Riveros-Mckay F., Kostadima M.A. (2016). The Allelic Landscape of Human Blood Cell Trait Variation and Links to Common Complex Disease. Cell.

[53] MacArthur J., Bowler E., Cerezo M., Gil L., Hall P., Hastings E., Junkins H., McMahon A., Milano A., Morales J. (2017). The new NHGRI-EBI Catalog of published genome-wide association studies (GWAS Catalog). Nucleic Acids Res..

[54] Ferrer-Admetlla A., Liang M., Korneliussen T., Nielsen R. (2014). On detecting incomplete soft or hard selective sweeps using haplotype structure. Mol. Biol. Evol..

[55] Szpiech Z.A., Hernandez R.D. (2014). selscan: an efficient multithreaded program to perform EHH-based scans for positive selection. Mol. Biol. Evol..

[56] Gay N.R., Gloudemans M., Antonio M.L., Abell N.S., Balliu B., Park Y., Martin A.R., Musharoff S., Rao A.S., Aguet F. (2020). Impact of admixture and ancestry on eQTL analysis and GWAS colocalization in GTEx. Genome Biol..

[57] Stranger B.E., Montgomery S.B., Dimas A.S., Parts L., Stegle O., Ingle C.E., Sekowska M., Smith G.D., Evans D., Gutierrez-Arcelus M. (2012). Patterns of cis regulatory variation in diverse human populations. PLoS Genet..

[58] Sankararaman S., Mallick S., Patterson N., Reich D. (2016). The Combined Landscape of Denisovan and Neanderthal Ancestry in Present-Day Humans. Curr. Biol..

[59] Vespasiani D.M., Jacobs G.S., Cook L.E., Brucato N., Leavesley M., Kinipi C., Ricaut F.-X., Cox M.P., Romero I.G. (2021). Denisovan introgression has shaped the immune system of present-day Papuans. bioRxiv.

[60] Gittelman R.M., Schraiber J.G., Vernot B., Mikacenic C., Wurfel M.M., Akey J.M. (2016). Archaic Hominin Admixture Facilitated Adaptation to Out-of-Africa Environments. Curr. Biol..

[61] Okada Y., Hirota T., Kamatani Y., Takahashi A., Ohmiya H., Kumasaka N., Higasa K., Yamaguchi-Kabata Y., Hosono N., Nalls M.A. (2011). Identification of nine novel loci associated with white blood cell subtypes in a Japanese population. PLoS Genet..

[62] Crosslin D.R., McDavid A., Weston N., Zheng X., Hart E., de Andrade M., Kullo I.J., McCarty C.A., Doheny K.F., Pugh E. (2013). Genetic variation associated with circulating monocyte count in the eMERGE Network. Hum. Mol. Genet..

[63] Jain D., Hodonsky C.J., Schick U.M., Morrison J.V., Minnerath S., Brown L., Schurmann C., Liu Y., Auer P.L., Laurie C.A. (2017). Genome-wide association of white blood cell counts in Hispanic/Latino Americans: the Hispanic Community Health Study/Study of Latinos. Hum. Mol. Genet..

[64] Chen M.-H., Raffield L.M., Mousas A., Sakaue S., Huffman J.E., Moscati A., Trivedi B., Jiang T., Akbari P., Vuckovic D. (2020). Trans-ethnic and Ancestry-Specific Blood-Cell Genetics in 746,667 Individuals from 5 Global Populations. Cell.

[65] Vuckovic D., Bao E.L., Akbari P., Lareau C.A., Mousas A., Jiang T., Chen M.-H., Raffield L.M., Tardaguila M., Huffman J.E. (2020). The Polygenic and Monogenic Basis of Blood Traits and Diseases. Cell.

[66] Humphreys K.L., Moore S.R., Davis E.G., MacIsaac J.L., Lin D.T.S., Kobor M.S., Gotlib I.H. (2019). DNA methylation of HPA-axis genes and the onset of major depressive disorder in adolescent girls: a prospective analysis. Transl. Psychiatry.

[67] Schartner C., Ziegler C., Schiele M.A., Kollert L., Weber H., Arolt V., Pauli P., Zwanzger P., Reif A., Deckert J. (2016). Hypomethylation of corticotropin releasing hormone receptor 1 promoter region: Converging evidence for a role in panic disorder. Eur. Neuropsychopharmacol..

[68] Jokinen J., Boström A.E., Dadfar A., Ciuculete D.M., Chatzittofis A., Åsberg M., Schiöth H.B. (2018). Epigenetic Changes in the CRH Gene are Related to Severity of Suicide Attempt and a General Psychiatric Risk Score in Adolescents. EBioMedicine.

[69] Pilling L.C., Atkins J.L., Duff M.O., Beaumont R.N., Jones S.E., Tyrrell J., Kuo C.-L., Ruth K.S., Tuke M.A., Yaghootkar H. (2017). Red blood cell distribution width: Genetic evidence for aging pathways in 116,666 volunteers. PLoS ONE.

[70] Kichaev G., Bhatia G., Loh P.-R., Gazal S., Burch K., Freund M.K., Schoech A., Pasaniuc B., Price A.L. (2019). Leveraging Polygenic Functional Enrichment to Improve GWAS Power. Am. J. Hum. Genet..

[71] Li X., Ito M., Zhou F., Youngson N., Zuo X., Leder P., Ferguson-Smith A.C. (2008). A maternal-zygotic effect gene, Zfp57, maintains both maternal and paternal imprints. Dev. Cell.

[72] Plant K., Fairfax B.P., Makino S., Vandiedonck C., Radhakrishnan J., Knight J.C. (2014). Fine mapping genetic determinants of the highly variably expressed MHC gene ZFP57. Eur. J. Hum. Genet..

[73] Mogil L.S., Andaleon A., Badalamenti A., Dickinson S.P., Guo X., Rotter J.I., Johnson W.C., Im H.K., Liu Y., Wheeler H.E. (2018). Genetic architecture of gene expression traits across diverse populations. PLoS Genet..

[74] Tehranchi A., Hie B., Dacre M., Kaplow I., Pettie K., Combs P., Fraser H.B. (2019). Fine-mapping *cis*-regulatory variants in diverse human populations. eLife.

[75] Liu X., Li Y.I., Pritchard J.K. (2019). Trans Effects on Gene Expression Can Drive Omnigenic Inheritance. Cell.

[76] Favé M.-J., Lamaze F.C., Soave D., Hodgkinson A., Gauvin H., Bruat V., Grenier J.-C., Gbeha E., Skead K., Smargiassi A. (2018). Gene-by-environment interactions in urban populations modulate risk phenotypes. Nat. Commun..

[77] Sella G., Barton N.H. (2019). Thinking About the Evolution of Complex Traits in the Era of Genome-Wide Association Studies. Annu. Rev. Genomics Hum. Genet..

[78] Akey J.M. (2009). Constructing genomic maps of positive selection in humans: where do we go from here?. Genome Res..

