# Supplementary material for: Genetic architecture of gene regulation in Indonesian populations identifies QTLs associated with global and local ancestries
Source: Am J Hum Genet. 2021 Dec 16;109(1):50–65. doi: 10.1016/j.ajhg.2021.11.017 (PMC8764200; doi:10.1016/j.ajhg.2021.11.017)
Supplement: Document S1. Figures S1–S13 and supplemental notes 1–4 [file mmc1.pdf]

The American Journal of Human Genetics, Volume *109*

Supplemental information

Genetic architecture of gene regulation in Indonesian populations identifies QTLs associated with global and local ancestries

Heini M. Natri, Georgi Hudjashov, Guy Jacobs, Pradiptajati Kusuma, Lauri Saag, Chelzie Crenna Darusallam, Mait Metspalu, Herawati Sudoyo, Murray P. Cox, Irene Gallego Romero, and Nicholas E. Banovich

Supplementary Information

Supplementary Notes 1–4 and Supplementary Figures 1–13

Supplementary Figure 1. Proportions of inferred Papuan ancestry and Denisovan introgression are highly correlated (Pearson's correlation coefficient 0.995).

Supplementary Figure 2. Enrichment of methylQTLs (a) and eQTLs (b) among DNase hypersensitive sites (DHS) and histone marks in ENCODE GM12878 and K562 cell lines.

Supplementary Note 1. Colocalized *cis*-eQTLs and *cis*-methylQTLs indicate shared causal variants.

Supplementary Figure 3. Distribution of the lower bounds of the prior probabilities (p12) that suggest colocalization across 4,639 tested methylQTL-eQTL pairs. As the posterior probability for colocalization is dependent on the prior probability, a post-hoc sensitivity analysis was used to determine the range of prior probabilities for which colocalization is supported. Pairs passing the colocalization threshold with a range of p12 values from $<1.0\times10^{-6}$ to 1.0×10^{-4} (lower bound of p12 below 1.0×10^{-6}) were considered to show robust support for colocalization.

Supplementary Figure 4. Genetically regulated promoter methylation alters target gene expression levels. Relationship between the absolute effect sizes of colocalized methylQTLs and eQTLs that show the same direction of effect (a) and opposing direction of effect (b) on the target trait. Pairs that share the same top-SNP are plotted and variants located on promoter regions are highlighted. Smoothed means based on linear models in the form y ~ x and 95% confidence intervals are shown for each set.

Supplementary Figure 5. Manhattan plots of the eQTL -log10(p-values) for a colocalized gene and an Indonesia-specific gene in the Indonesian data and three European eQTL datasets.

Supplementary Figure 6. Effect sizes of colocalized eQTLs in the Indonesian and European datasets.

Supplementary Figure 7. Expression levels of colocalized and Indonesia-specific eGenes in the Indonesian data and GTEx whole blood data.

Supplementary Figure 8. Relationship between absolute differences in ALT allele frequencies and expression levels of the Indonesia-specific eQTLs between Indonesia and Europe.

Supplementary Note 2. Qualities of eQTLs driven by archaic ancestry.

Supplementary Figure 9. Minor allele frequencies (MAF) and absolute effect sizes of eQTLs driven by Denisovan or Neanderthal introgression and eQTLs not driven by archaic introgression ("other") before (a) and after (b, c) allele frequency matching. t-test p-values are indicated for each pairwise comparison.

Supplementary Figure 10. Minor allele frequencies (MAF) and absolute effect sizes of methylQTLs driven by Denisovan or Neanderthal introgression and methylQTLs not driven by archaic introgression ("other") before (a) and after (b, c) allele frequency matching. t-test p-values are indicated for each pairwise comparison.

Supplementary Figure 11. a: Power to detect QTLs as a function of MAF when N=115. b: Minimum detectable slope in simple linear regression as a function of MAF, with various power levels. In both models, the type I error rate was set to 0.01 and the SD of the linear model to 0.2.

Supplementary Note 3. Identifying ancestry-driven QTLs under positive selection.

Supplementary Figure 12. Modern ancestry and archaic introgression -driven eQTLs overlapping genomic windows that show evidence of recent positive selection in each of the three study populations. Variance in QTL genotype explained (R2) is shown on the x-axis of each plot. Variants with R2 > 0.7 were considered to be highly correlated with ancestry (vertical line). The proportion of positions within 50Kb windows that show an

nSL > 2 is shown on the y-axis. Genomic windows with this proportion >0.3 were considered to be showing evidence of positive selection (horizontal line). The target genes of eQTLs showing both a significant correlation with ancestry and evidence of selection are labeled.

Supplementary Figure 13. Modern ancestry and archaic introgression -driven methylQTLs overlapping genomic windows that show evidence of recent positive selection in each of the three study populations. Variance in QTL genotype explained (R2) is shown on the x-axis of each plot. Variants with R2 > 0.7 were considered to be highly correlated with ancestry (vertical line). The proportion of positions within 50Kb windows that show an nSL > 2 is shown on the y-axis. Genomic windows with this proportion >0.3 were considered to be showing evidence of positive selection (horizontal line). The target CpGs of methylQTLs showing both a significant correlation with ancestry and evidence of selection are labeled.

Supplementary Note 4. Qualities of the Denisovan driven methylQTLs that colocalize with platelet count GWAS.

Supplementary Tables 1–10

Supplementary Table 1: Sample information.

Supplementary Table 2: Sequencing batch information for RNAseq samples.

Supplementary Table 3: Sub-Saharan African samples used in archaic introgression inference.

Supplementary Table 4. Numbers of colocalized and non-colocalized genes in each pairwise comparison between Indonesia and the European cohorts. eGenes with an FDR-p<0.1 were included in testing. The analysis was carried out separately using the top genes from each dataset. Proportions of robustly colocalized genes out of all tested genes are indicated in percentage.

Supplementary Table 5. Descriptions of the target genes of Indonesian eQTLs that show no evidence of colocalization with any of the tested European datasets and harbor an Indonesia-specific eQTL according to the *mashr* analysis.

Supplementary Table 6: p-values for overrepresentation (Fisher's test) of modern and archaic LAI -driven QTLs among the genomic windows under positive selection in each population. QTLs with an FDR-p < 0.01 in the permutation-based analysis were included in testing. QTLs driven by Denisovan or Neanderthal ancestry with an R2>0.7 were considered LAI driven. Windows with a proportion of SNPs with an absolute nSL > 2 of 30% were considered to be under positive selection.

Supplementary Table 7: Ancestry-driven QTLs under positive selection. For methylQTLs, annotations based on the Illumina EPIC array manifest are provided.

Supplementary Table 8: Summary of Indonesian eQTLs and methylQTLs that colocalize with each of the 36 hematological GWAS traits reported by Astle et al. 2016.

Supplementary Table 9. Summary of European eQTLs that colocalize with each of the 36 hematological GWAS traits reported in the Astle et al. 2016.

Supplementary Table 10. Qualities of the Denisovan driven methylQTLs that colocalize with platelet count GWAS.

Supplementary Data Files 1–11

Data S1: Permutation-significant eQTLs. Columns: target, chromosome, target start, N of tested SNPs, top-SNP distance to the target, rsID, top-SNP position, slope, nominal *p*, FDR-*p*

Data S2: Permutation-significant methylQTLs. Columns: target, chromosome, target position, N of tested SNPs, top-SNPdistance to the target, rsID, top-SNP position, slope, nominal *p*, FDR-*p*

Data S3: Nominal eQTL statistics. Columns: target, target chromosome, target start, N of tested SNPs, SNP distance to the target, rsID, SNP position, nominal *p*, slope.

Data S4. eQTL-methylQTL colocalization results for robust colocalized pairs. Columns: Target CpG, Target gene, number of tested SNPs, Tag SNP 1 and Tag SNP 2 (testing between all independent signals), PP0, PP1, PP2, PP3, PP4, PP4/PP3, lower bound of prior probability for colocalization (p12) that passes the threshold.

Data S5: eQTL-LAI correlation results for Papuan ancestry. Columns: chr, pos, R^2, p, target

Data S6: eQTL-LAI correlation results for Denisovan introgression. Columns: chr, pos, R^2, p, target

Data S7: eQTL-LAI correlation results for Neanderthal introgression. Columns: chr, pos, R^2, p, target

Data S8: methylQTL-LAI correlation results for Papuan ancestry. Columns: chr, pos, R^2, p, target

Data S9: methylQTL-LAI correlation results for Denisovan introgression. Columns: chr, pos, R^2, p, target

Data S10: methylQTL-LAI correlation results for Neanderthal introgression. Columns: chr, pos, R^2, p, target

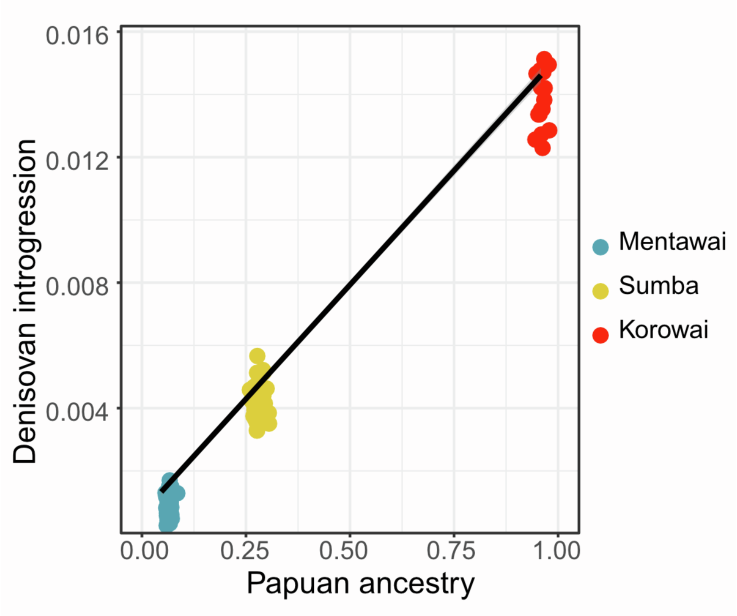

Supplementary Figure 1. Proportions of inferred Papuan ancestry and Denisovan introgression are highly correlated (Pearson's correlation coefficient 0.995).

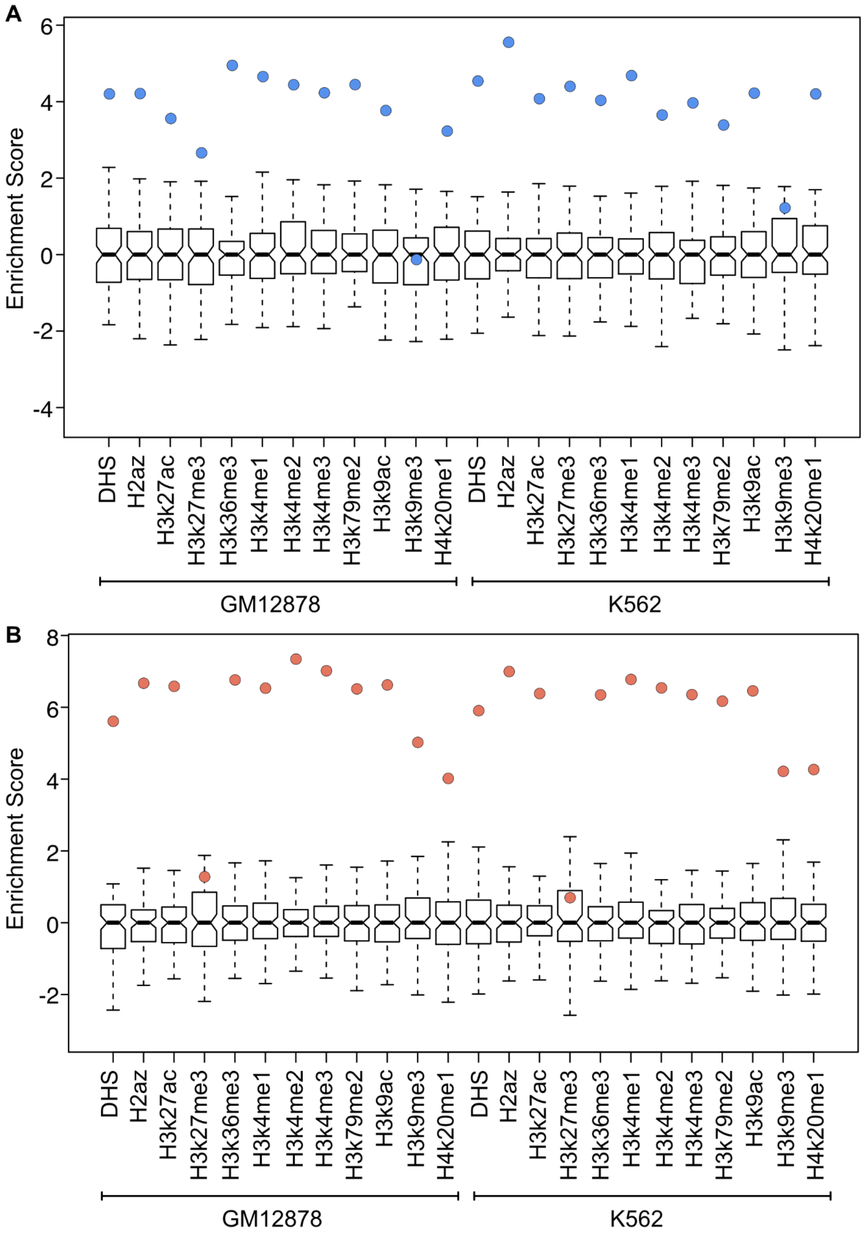

Supplementary Figure 2. Enrichment of methylQTLs (**A**) and eQTLs (**B**) among DNase hypersensitive sites (DHS) and histone marks in ENCODE GM12878 and K562 cell lines.

Supplementary Note 1. *Colocalized cis-eQTLs and cis-methylQTLs indicate shared causal variants.* We integrated the methylQTL and eQTL calls to gain insight into how genetic regulation of CpG methylation may contribute to the regulation of gene expression. 1,140 of the unique permutation significant eVariants were also nominally associated (nominal $p<1\times10^{-7}$) with the methylation of at least one CpG, and 2,015 of the unique permutation-significant methylVariants were also nominally associated with the expression of at least one gene,

suggesting that a substantial number of causal eVariants may also be causal methylVariants, and vice versa. This overlap corresponds to 4,639 CpG-gene combinations potentially harboring a common causal variant (CCV). We tested for colocalization between these pairs of CpGs and genes using a Bayesian method as implemented in *coloc* v4. Among the tested pairs, we detected 720 (15.5%) eQTL-methylQTL pairs that showed robust support for colocalization with a wide range of prior probabilities for a common causal variant (Supplementary Figure 3, Methods).

We explored the direction of the effects of top-SNPs associated with the 720 CpG and gene pairs that exhibit a high probability of a single shared causal variant. Concordant with previous studies[1,2], 56.1% of these eQTL-methylQTL pairs show an opposite effect direction. This proportion is 61.9% when only including pairs that had the same top-SNP based on QTL *p*-values, and 69.1% when further limiting to CpGs that are located on promoter regions. Pairs that show an opposite effect also show a high correlation between the absolute effect sizes (Pearson's correlation 0.49, $p < 2.2\times10^{-16}$), while pairs with the same effect directions don't (Pearson's correlation 0.03, p=0.64) (Supplementary Figure 4). Colocalized CpGs located on promoters are more likely to show an opposite direction in effect with the gene than CpGs located outside promoters or enhancers (Fisher's test $p=3.835\times10^{-6}$), but the same is not observed for CpGs located on enhancers when compared to those located outside promoters or enhancers (Fisher's test p=0.6808).

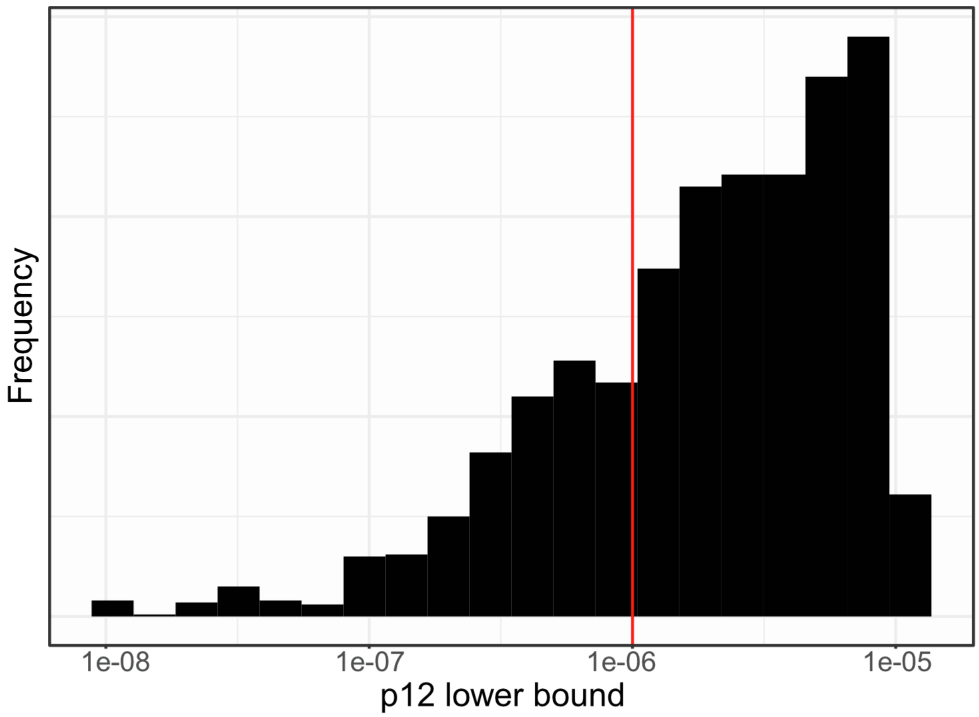

Supplementary Figure 3. Distribution of the lower bounds of the prior probabilities (p12) that suggest colocalization across 4,639 tested methylQTL-eQTL pairs. As the posterior probability for colocalization is dependent on the prior probability, a post-hoc sensitivity analysis was used to determine the range of prior probabilities for which colocalization is supported. Pairs passing the colocalization threshold with a range of p12 values from $<1.0\times10^{-6}$ to 1.0×10^{-4} (lower bound of p12 below 1.0×10^{-6}) were considered to show robust support for colocalization.

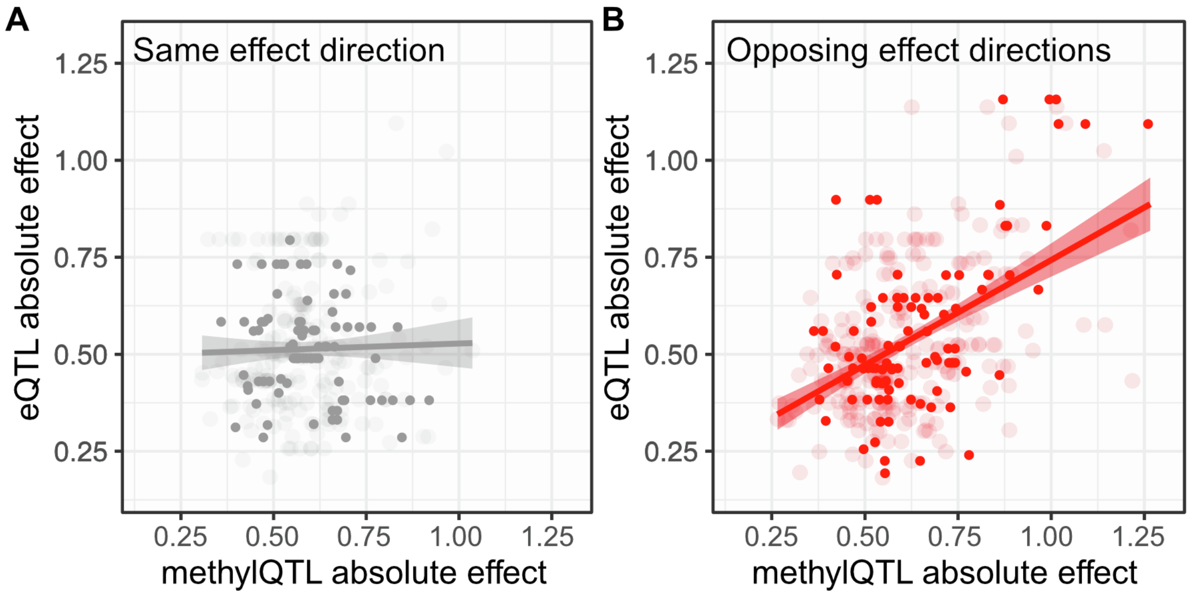

Supplementary Figure 4. Genetically regulated promoter methylation alters target gene expression levels. Relationship between the absolute effect sizes of colocalized methylQTLs and eQTLs that show the same direction of effect (**A**) and opposing direction of effect (**B**) on the target trait. Pairs that share the same top-SNP are plotted and variants located on promoter regions are highlighted. Smoothed means based on linear models in the form y ~ x and 95% confidence intervals are shown for each set.

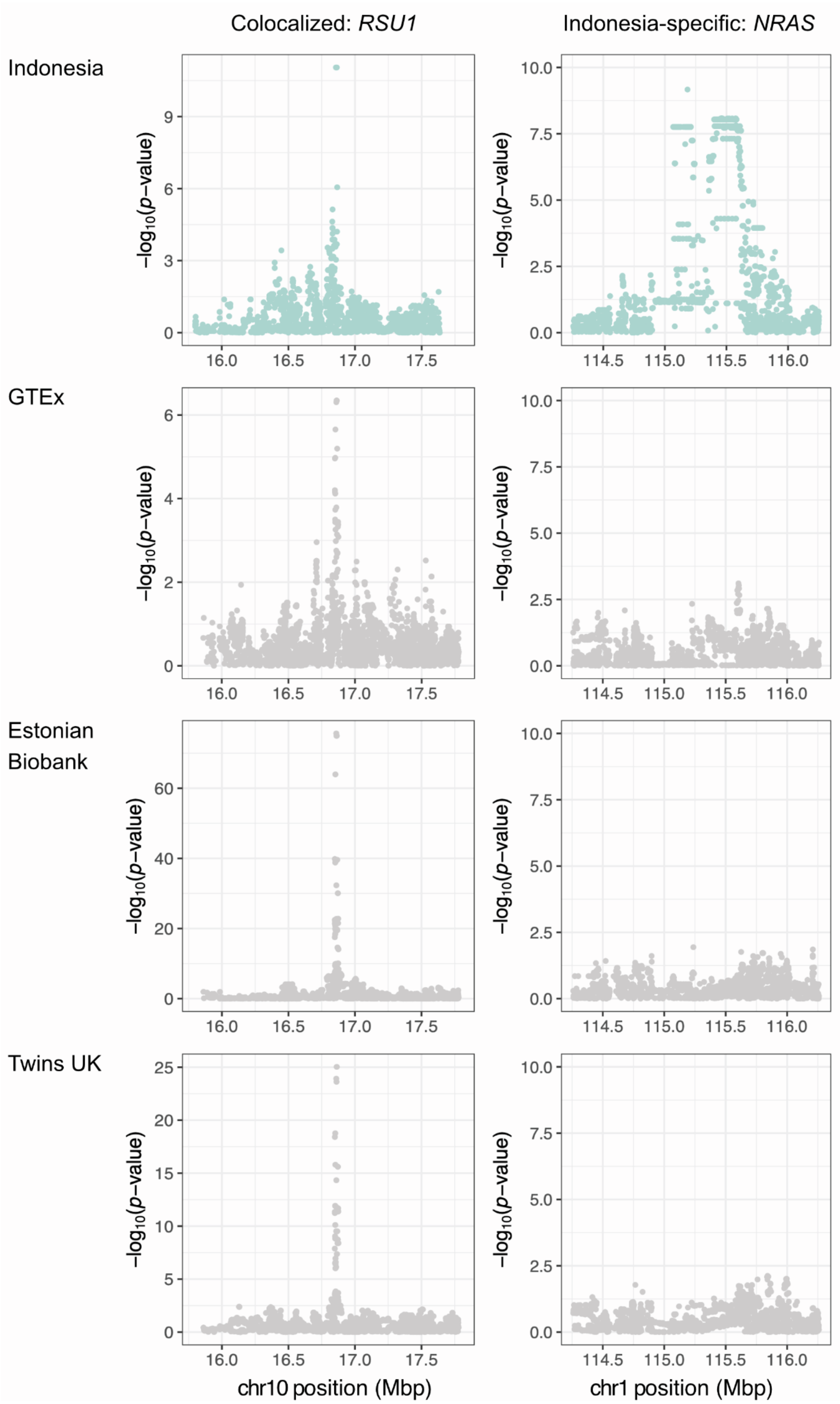

Supplementary Figure 5. Manhattan plots of the eQTL -log10(*p*-values) for a colocalized gene and an Indonesia-specific gene in the Indonesian data and three European eQTL datasets.

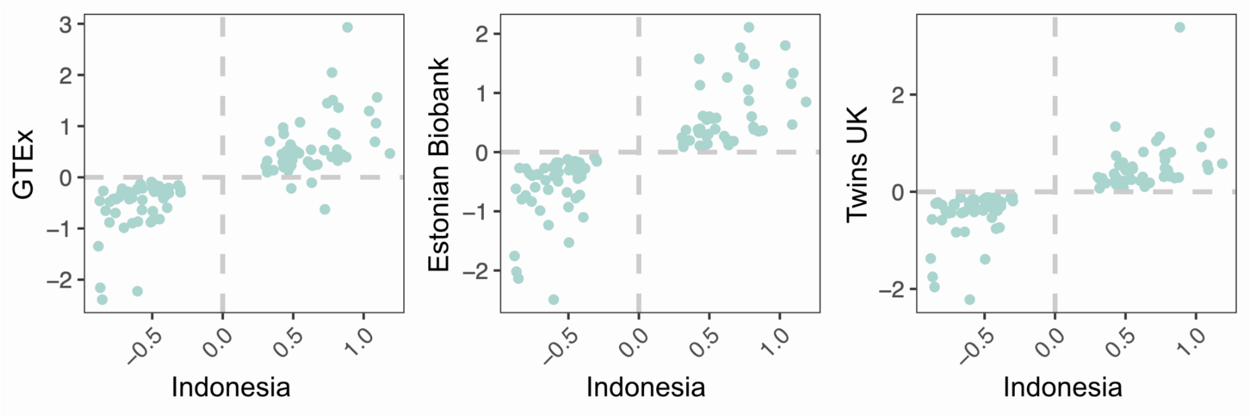

Supplementary Figure 6. Effect sizes of colocalized eQTLs in the Indonesian and European datasets.

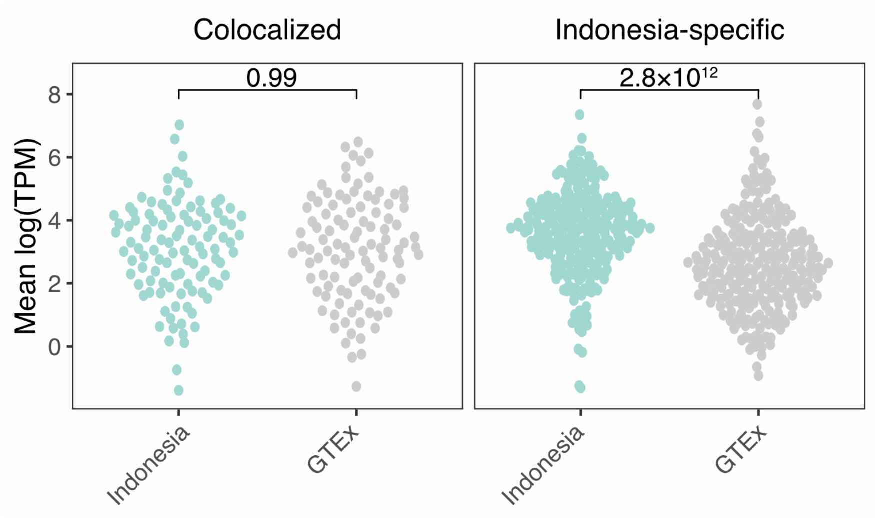

Supplementary Figure 7. Expression levels of colocalized and Indonesia-specific eGenes in the Indonesian data and GTEx whole blood data.

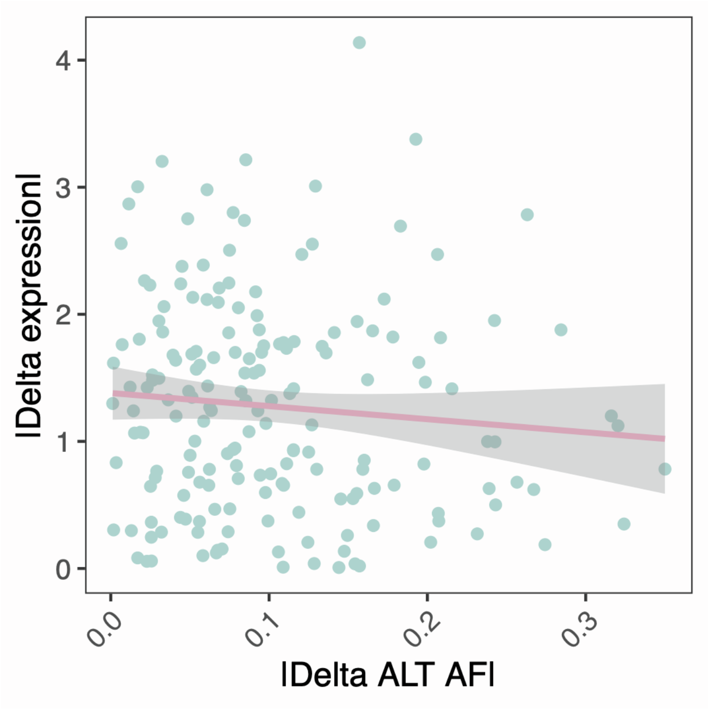

Supplementary Figure 8. Relationship between absolute differences in ALT allele frequencies and expression levels of the Indonesia-specific eQTLs between Indonesia and Europe.

Supplementary Note 2. *Qualities of eQTLs driven by archaic ancestry.* We compared the absolute effect sizes of the archaic ancestry-driven QTLs and the effect sizes of the significant QTLs not driven by archaic ancestry. Denisovan and Neanderthal ancestry-driven eQTLs (Supplementary Figure 9) and methylQTLs (Supplementary Figure 10) exhibit significantly larger absolute effect sizes than methylQTLs not driven by archaic ancestry. However, as the minor allele frequencies of the archaic driven QTLs are lower, we are less powered to detect small effect QTLs driven by archaic ancestry (Supplementary Figure 11). We performed allele frequency matching with the nearest neighbor matching method of the R package *MatchIt* v3.0.2[3]. There were no significant differences in the mean absolute effect sizes of the MAF matched sets and the archaic driven QTLs.

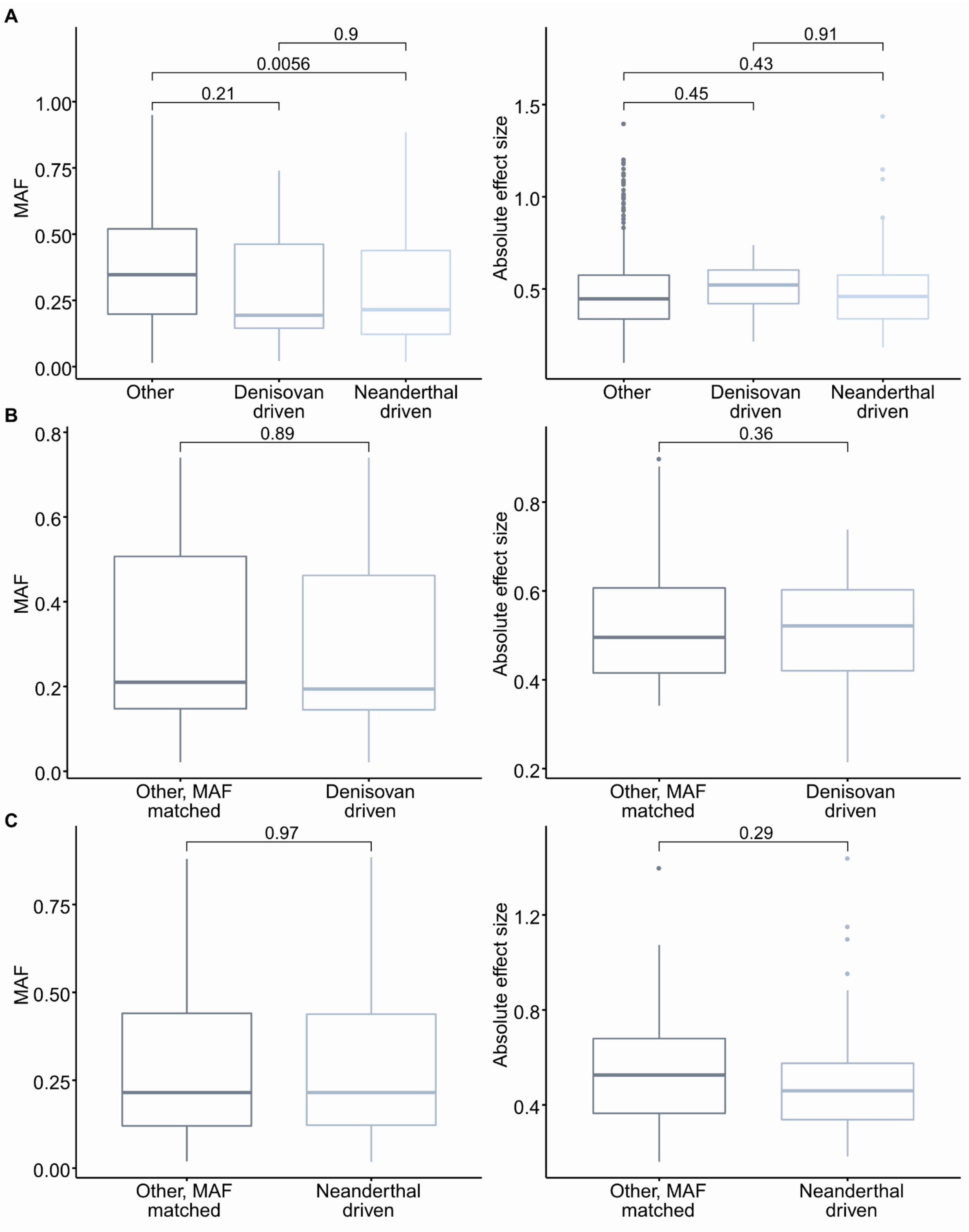

Supplementary Figure 9. Minor allele frequencies (MAF) and absolute effect sizes of eQTLs driven by Denisovan or Neanderthal introgression and eQTLs not driven by archaic

introgression (“other”) before (**A**) and after (**B**, **C**) allele frequency matching. t-test *p*-values are indicated for each pairwise comparison.

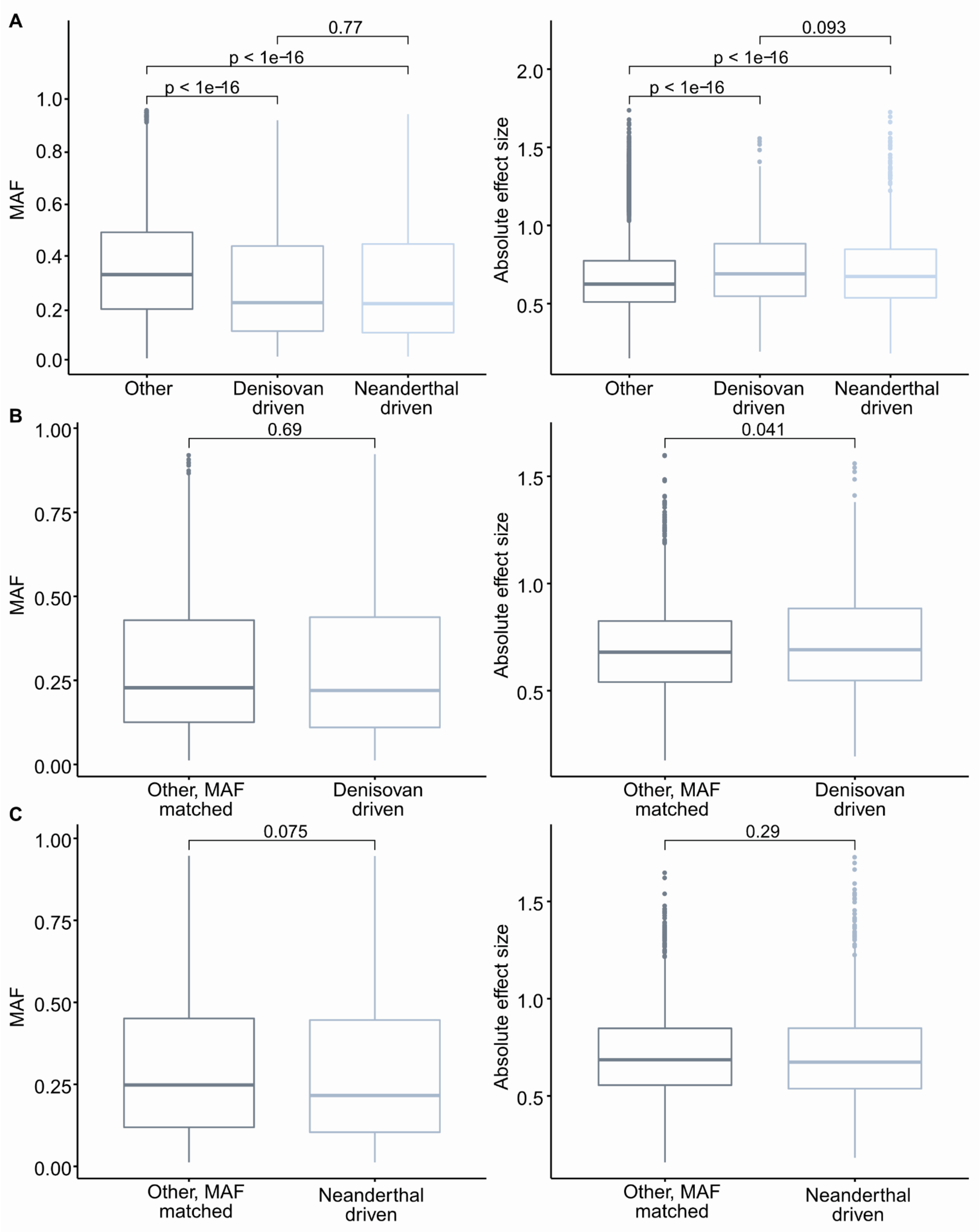

Supplementary Figure 10. Minor allele frequencies (MAF) and absolute effect sizes of methylQTLs driven by Denisovan or Neanderthal introgression and methylQTLs not driven by archaic introgression (“other”) before (**A**) and after (**B**, **C**) allele frequency matching. t-test *p*-values are indicated for each pairwise comparison.

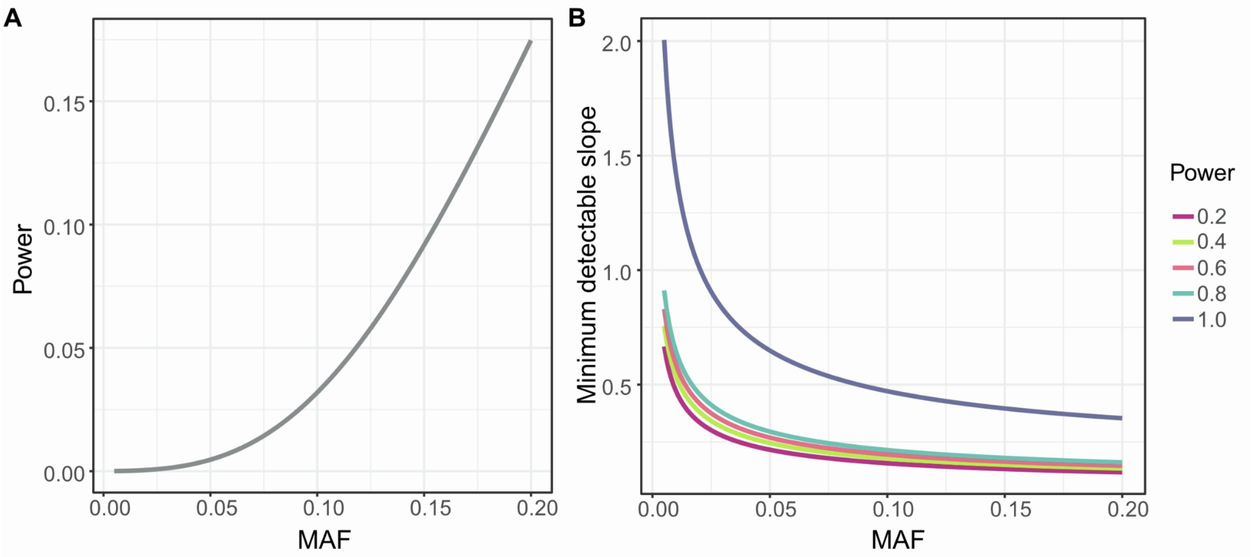

Supplementary Figure 11. A: Power to detect QTLs as a function of MAF when N=115. **B:** Minimum detectable slope in simple linear regression as a function of MAF, with various power levels. In both models, the type I error rate was set to 0.01 and the SD of the linear model to 0.2.

Supplementary Note 3. ***Identifying ancestry-driven QTLs under positive selection.*** We asked whether positive selection on ancestry informative regulatory variants may have contributed to the between-population variation in molecular phenotypes in the region. We used a haplotype-based nSL selection scan (Methods) to identify genomic regions that show signs of past selective sweeps and found 4.7%, 4.6%, and 5.0% of the genome to be under positive selection in Mentawai, Sumba, and Korowai, respectively. We used a colocalization-based method (Methods) to identify shared signals between the QTLs and nSL and detect no significant overlap between QTLs and selection.

Additionally, we overlapped the ancestry-driven QTLs with genomic regions with strong evidence of positive selection. We detect no clear overrepresentation of ancestry-driven QTLs among these regions (Supplementary Table 5). However, we find individual QTLs that overlap them (Supplementary Table 6, Supplementary Figures 10, 11), including one Papuan-driven

eQTL under selection in Mentawai, one Neanderthal-driven eQTL in Korowai, and one in Mentawai, as well as Papuan-driven methylQTLs under selection in Mentawai (2), Sumba (2), and Korowai (12).

Moreover, we detect one Denisovan-driven methylQTL under selection in Korowai, associated with a CpG located on the promoter of *ZNF426*. Genetic variation associated with *ZNF426* and other KRAB-ZNF genes has previously been identified on candidate regions for positive selection in multiple human populations[4,5]. Further, we identified 13, 6, and 3 Neanderthal-driven methylQTLs under selection in Mentawai, Sumba, and Korowai (Supplementary Table 6). For example, a Neanderthal-driven methylQTL under selection in Mentawai was also nominally associated ($p = 2.596 \times 10^{-7}$) with *CATSPER3* (Cation Channel Sperm Associated 3) expression, which was differentially expressed between Mentawai and Korowai, as well as Sumba and Korowai[6]. Neanderthal variation in sodium channel genes was recently linked to increased pain sensitivity in modern humans[7].

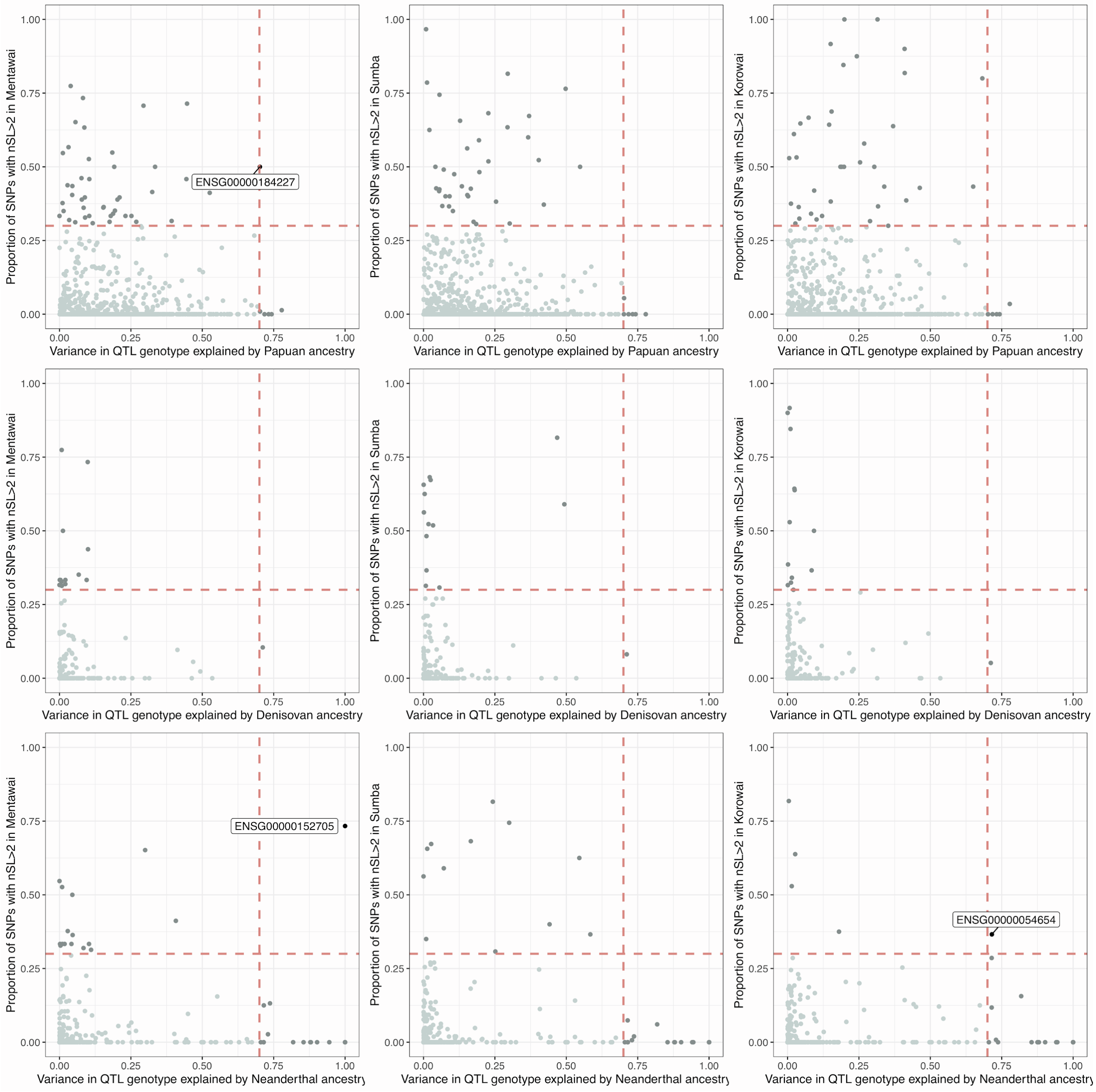

Supplementary Figure 12. Modern ancestry and archaic introgression -driven eQTLs overlapping genomic windows that show evidence of recent positive selection in each of the three study populations. Variance in QTL genotype explained (R^2) is shown on the x-axis of each plot. Variants with $R^2 > 0.7$ were considered to be highly correlated with ancestry (vertical line). The proportion of positions within 50Kb windows that show an nSL > 2 is shown on the y-axis. Genomic windows with this proportion >0.3 were considered to be showing evidence of positive selection (horizontal line). The target genes of eQTLs showing both a significant correlation with ancestry and evidence of selection are labeled.

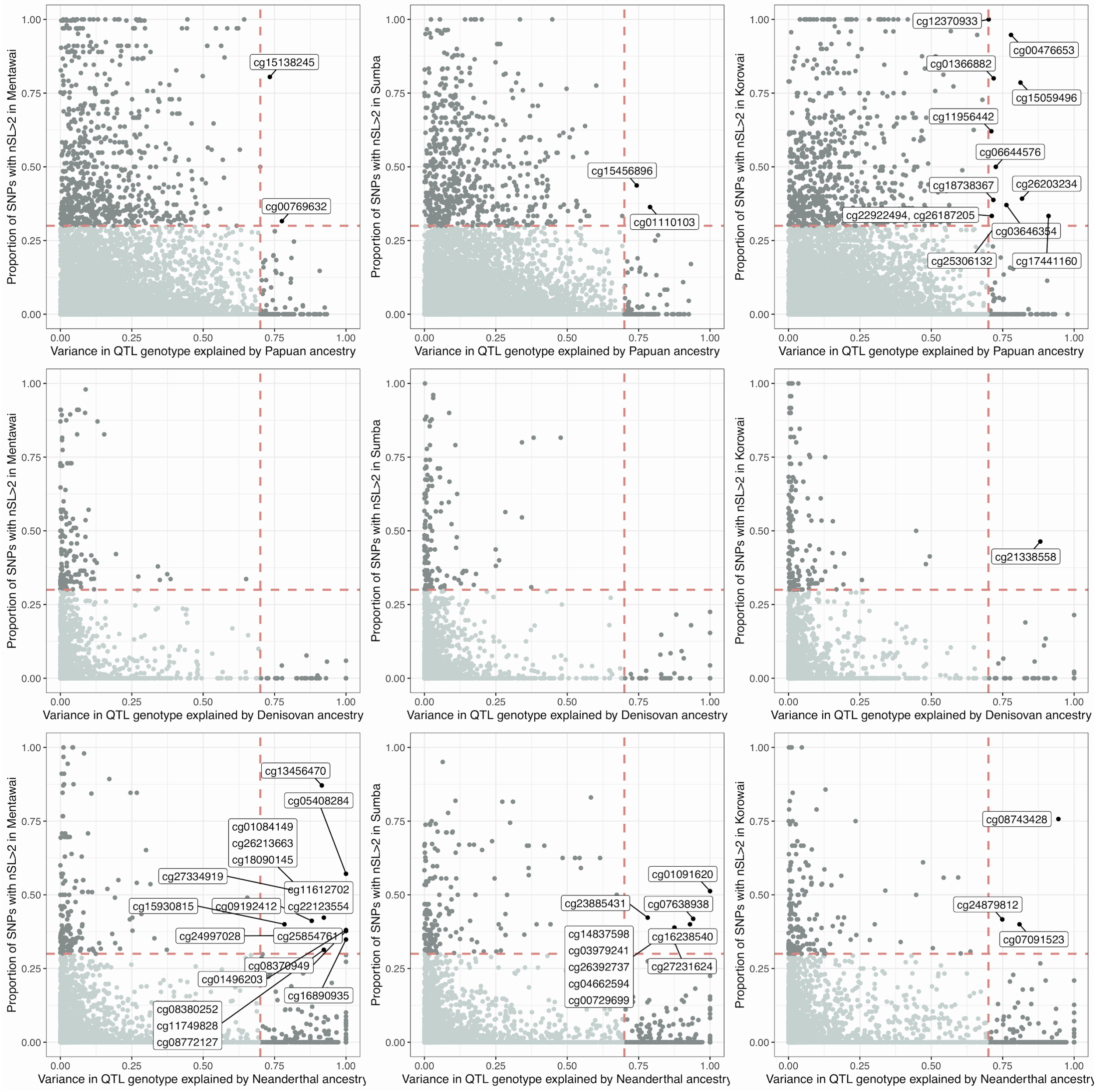

Supplementary Figure 13. Modern ancestry and archaic introgression driven methylQTLs overlapping genomic windows that show evidence of recent positive selection in each of the three study populations. Variance in QTL genotype explained (R^2) is shown on the x-axis of each plot. Variants with $R^2 > 0.7$ were considered to be highly correlated with ancestry (vertical line). Proportion of positions within 50Kb windows that show an nSL > 2 is shown on the y-axis. Genomic windows with this proportion >0.3 were considered to be showing evidence of positive selection (horizontal line). The target CpGs of methylQTLs showing both a significant correlation with ancestry and evidence of selection are labeled.

Supplementary Note 4. Qualities of the Denisovan-driven GWAS-eQTLs. We assessed the credibility of the four Denisovan-driven methylQTLs that colocalize with platelet count GWAS loci. First, we assessed our ability to correctly call genotypes on these positions, to correctly call methylQTLs, and to identify the correlation between the genotypes and the numbers of inferred Denisovan alleles. We used mappability scores generated with Umap[8] to assess mappability on regions overlapping these methylVariants. Umap calculates the single-read mappability of genome for a range of sequencing read lengths, the single-read mappability of a genomic region being defined as a fraction of that region that overlaps with at least one uniquely mappable kmer. For a given sequence, mappability of 1 means that the sequence is uniquely mappable on the forward strand. Uniquely mappable regions with various kmers were downloaded from the Hoffman Lab website (Web Resources). All four variants are located on regions that are uniquely mappable with kmer lengths of 24, 36, 50, and 100bp, apart from chr6:29,799,383 which is on a region that is only uniquely mappable with kmers 36, 50, and 100bp. All four variants were called with high read depth, ranging from 29,492 to 37,373. All four variants have adequate MAFs, ranging from 0.161 to 0.302. All four methylQTLs show large effect sizes, the absolute effect size ranging from 0.66 to 0.88. Furthermore, all methylVariants show a clear correlation with the number of inferred Denisovan alleles, R^2 ranging from 0.73 to 0.90. The methylVariants associated with cg03118604 and cg03861427 are located within 741bp of each other and are in LD.

Then, we assessed whether sequence similarity across the genome could lead to spurious signals in the CpG methylation measurements using the Illumina EPIC array. We used megablast of BLASTN[9] to map the forward sequences flanking the CpGs to the human reference genome. All four sequences map to the HLA locus with high confidence and do not map to other regions (Supplementary Table 10).

References

1. Pierce, B.L., Tong, L., Argos, M., Demanelis, K., Jasmine, F., Rakibuz-Zaman, M., Sarwar, G., Islam, M.T., Shahriar, H., Islam, T., et al. (2018). Co-occurring expression and methylation QTLs allow detection of common causal variants and shared biological mechanisms. Nat. Commun. *9*, 804.

2. Banovich, N.E., Lan, X., McVicker, G., van de Geijn, B., Degner, J.F., Blischak, J.D., Roux, J., Pritchard, J.K., and Gilad, Y. (2014). Methylation QTLs are associated with coordinated changes in transcription factor binding, histone modifications, and gene expression levels. PLoS Genet. *10*, e1004663.

3. Ho, D.E., Imai, K., King, G., and Stuart, E.A. (2011). MatchIt: Nonparametric Preprocessing for Parametric Causal Inference. Journal of Statistical Software *42*,.

4. Perdomo-Sabogal, Á., and Nowick, K. (2019). Genetic Variation in Human Gene Regulatory Factors Uncovers Regulatory Roles in Local Adaptation and Disease. Genome Biol. Evol. *11*, 2178–2193.

5. Ávila-Arcos, M.C., McManus, K.F., Sandoval, K., Rodríguez-Rodríguez, J.E., Villa-Islas, V., Martin, A.R., Luisi, P., Peñaloza-Espinosa, R.I., Eng, C., Huntsman, S., et al. (2020). Population History and Gene Divergence in Native Mexicans Inferred from 76 Human Exomes. Mol. Biol. Evol. *37*, 994–1006.

6. Natri, H.M., Bobowik, K.S., Kusuma, P., Crenna Darusallam, C., Jacobs, G.S., Hudjashov, G., Lansing, J.S., Sudoyo, H., Banovich, N.E., Cox, M.P., et al. (2020). Genome-wide DNA methylation and gene expression patterns reflect genetic ancestry and environmental differences across the Indonesian archipelago. PLoS Genet. *16*, e1008749.

7. Zeberg, H., Dannemann, M., Sahlholm, K., Tsuo, K., Maricic, T., Wiebe, V., Hevers, W., Robinson, H.P.C., Kelso, J., and Pääbo, S. (2020). A Neanderthal Sodium Channel Increases Pain Sensitivity in Present-Day Humans. Curr. Biol.

8. Karimzadeh, M., Ernst, C., Kundaje, A., and Hoffman, M.M. (2018). Umap and Bismap: quantifying genome and methylome mappability. Nucleic Acids Res. *46*, e120.

9. Zhang, Z., Schwartz, S., Wagner, L., and Miller, W. (2000). A greedy algorithm for aligning

DNA sequences. J. Comput. Biol. *7*, 203–214.
