# Supplementary material for: Genetic architecture of gene regulation in Indonesian populations identifies QTLs associated with global and local ancestries
Source: Am J Hum Genet. 2021 Dec 16;109(1):50–65. doi: 10.1016/j.ajhg.2021.11.017 (PMC8764200; doi:10.1016/j.ajhg.2021.11.017)
Supplement: Document S2. Article plus supplemental information [file mmc22.pdf]

ARTICLE

Genetic architecture of gene regulation in Indonesian populations identifies QTLs associated with global and local ancestries

Heini M. Natri,[1,2] Georgi Hudjashov,[3,4] Guy Jacobs,[5,6] Pradiptajati Kusuma,[6,7] Lauri Saag,[8] Chelzie Crenna Darusallam,[7] Mait Metspalu,[8] Herawati Sudoyo,[7] Murray P. Cox,[3,12] Irene Gallego Romero,[4,9,10,11,12] and Nicholas E. Banovich[2,12,*]

Summary

Lack of diversity in human genomics limits our understanding of the genetic underpinnings of complex traits, hinders precision medicine, and contributes to health disparities. To map genetic effects on gene regulation in the underrepresented Indonesian population, we have integrated genotype, gene expression, and CpG methylation data from 115 participants across three island populations that capture the major sources of genomic diversity in the region. In a comparison with European datasets, we identify eQTLs shared between Indonesia and Europe as well as population-specific eQTLs that exhibit differences in allele frequencies and/or overall expression levels between populations. By combining local ancestry and archaic introgression inference with eQTLs and methylQTLs, we identify regulatory loci driven by modern Papuan ancestry as well as introgressed Denisovan and Neanderthal variation. GWAS colocalization connects QTLs detected here to hematological traits, and further comparison with European datasets reflects the poor overall transferability of GWAS statistics across diverse populations. Our findings illustrate how population-specific genetic architecture, local ancestry, and archaic introgression drive variation in gene regulation across genetically distinct and in admixed populations and highlight the need for performing association studies on non-European populations.

Introduction

As we move into the age of precision medicine, the systematic undersampling of global genetic diversity limits our ability to broadly apply biomedical research efforts across diverse ethnicities and population backgrounds.[1,2] Indeed, the vast majority of human genomics studies to date have been conducted in individuals with European ancestry, who account for a minority of the global population.[3] To gain a comprehensive understanding of the genetic architecture of complex diseases and deliver on the promise of genomic medicine, it is critical to expand human genomics studies into diverse populations. The collection of multi-modal genomic data from traditionally undersampled populations will allow for the mapping of genetic associations with molecular phenotypes and integration with genome-wide association studies (GWASs) to fully understand the degree to which population differences impact genetic architecture.[4]

The Indonesian archipelago is one such undersampled region, absent from all existing large-scale catalogs of human diversity. Genetically and geographically structured, with a genomic cline of Asian to Papuan ancestry stretching from west to east,[5,6] Indonesia is the fourth largest country in the world by population. Its tropical location makes it an epicenter of infectious disease diversity both past and present, making it possible that individuals from the region have adapted to local immune challenges over evolutionary time.[7] We have previously described differences in gene expression and CpG methylation between Indonesian island populations associated with their genome-wide proportions of Papuan ancestry.[8] To investigate the effects of modern and archaic local ancestry on gene regulation in Indonesians, here we integrate genome-wide genotype data with gene expression and DNA methylation measurements from 115 Indonesian individuals. Using this rich multimodal dataset, we construct maps of eQTLs and methylQTLs and identify variants contributing to population differences—both within Indonesia and globally—in regulatory architecture.

Material and methods

Ethical approvals and dataset description

All samples were obtained from adult human male subjects. For full information about the new and published samples used in this study, refer to Tables S1 and S2. All samples used in this study

[1]Center for Evolution and Medicine, School of Life Sciences, Arizona State University, Tempe, AZ 85281, USA; [2]The Translational Genomics Research Institute, Phoenix, AZ 85004, USA; [3]Statistics and Bioinformatics Group, School of Fundamental Sciences, Massey University, Palmerston North 4410, New Zealand; [4]Centre for Genomics, Evolution and Medicine, Institute of Genomics, University of Tartu, Tartu 51010, Estonia; [5]Leverhulme Centre for Human Evolutionary Studies, Department of Archaeology, University of Cambridge, Cambridge CB2 1QH, UK; [6]Complexity Institute, Nanyang Technological University, Singapore, 637460; [7]Laboratory of Genome Diversity and Disease, Eijkman Institute for Molecular Biology, Jakarta 10430, Indonesia; [8]Institute of Genomics, University of Tartu, Tartu 51010, Estonia; [9]Melbourne Integrative Genomics, University of Melbourne, Parkville, VIC 3010, Australia; [10]School of BioSciences, University of Melbourne, Parkville, VIC 3010, Australia; [11]Centre for Stem Cell Systems, University of Melbourne, Parkville, VIC 3010, Australia
[12]These authors contributed equally
*Correspondence: nbanovich@tgen.org
https://doi.org/10.1016/j.ajhg.2021.11.017.
© 2021 The Author(s). This is an open access article under the CC BY license (http://creativecommons.org/licenses/by/4.0/).

were previously collected by H.S., J. Stephen Lansing, and an Indonesian team from the Eijkman Institute for Molecular Biology, Jakarta, Indonesia, with the assistance of Indonesian Public Health clinic staff. Collections followed protocols for the protection of human subjects established by institutional review boards at the Eijkman Institute (EIREC #90 and EIREC #126) and the University of Melbourne (Human Ethics Sub-Committee approval 1851639.1). All individuals gave written informed consent for participation in the study. Permission to conduct research in Indonesia was granted by the Indonesian Institute of Sciences and by the Ministry for Research, Technology, and Higher Education. Whole blood sample collection was carried out as described.[8] The gene expression and methylation data were previously published.[8]

Here, we report two new genomic datasets: (1) 42 samples genotyped with the Illumina Infinium Omni2.5-8 v1.3 BeadChip array, including five Korowai samples from New Guinea, 18 samples from Mentawai, western Indonesia, and 19 samples from Sumba, eastern Indonesia and (2) complete genomes for 70 samples, including 11 Korowai, 30 Mentawai, and 29 Sumba samples.

Whole-genome sequencing and data processing

Whole blood DNA from all individuals was extracted with Gentra Puregene for human whole blood kit (QIAGEN) and MagAttract HMW DNA kit (QIAGEN) according to the manufacturer's instructions. Approximately 1.3 μg of DNA from each of the 73 individuals were sent to Garvan and sequenced with TruSeq Nano v2.5 to an expected mean depth of 30×.

The newly generated genome sequences were processed closely following a previously described protocol[6] with the resources of the University of Tartu High Performance Computing Center.[9] Briefly, we first aligned the reads to the "decoy" version of the GRCh37 human reference sequence (hs37d5). After alignment, and keeping only properly paired reads that mapped to the same chromosome, the autosomal sequencing depth across the samples used in downstream analyses was as follows: min = 31.5×, Q1 = 35.3×, median = 36×, Q3 = 36.5×, max = 39.5×. Base-calling was undertaken with GATK best practices.[10,11] Following the generation of per-sample gVCF files with GATK4 HaplotypeCaller, single sample gVCFs were combined into multi-sample files with CombineGVCFs, and joint genotyping was performed with GATK4 GenotypeGVCFs, outputting all sites to a multi-sample VCF. To maximize the SNP discovery and phasing power, we used approximately 900 complete genomes in a multi-sample calling pipeline. In addition to the newly generated genomes, these included complete genome sequences from SGDP[12] and IGDP[6] projects, Malaspinas et al.,[13] Vernot et al.,[14] Lan et al.,[15] and the HiSeqX Diversity Cohort of Polaris project (web resources) as well as approximately 100 unpublished genome sequences from Estonia and Papua. SNP calling was performed on the combined dataset and published genomes were analyzed from raw reads exactly as they were for the new sequence data. Using bcftools v1.9,[16] we applied the following filters to each genotype call in multisample VCF files: base depth (DP) ≥ 8× and ≤ 400× and genotype quality (GQ) ≥ 30. Only bi-allelic SNPs and invariable reference sites were kept.

The published data included seven Korowai and ten Mentawai samples, however, two first-degree relatives (MTW-024 and MTW-066) were excluded from further analysis.[6] Our final whole-genome sequencing (WGS) dataset, therefore, included 84 samples from three target groups: 17 Korowai, 38 Mentawai, and 29 Sumba.

Next, modern human multi-sample VCF files were merged with two archaic individuals: Denisovan[17] and Neanderthal.[18] Positions with missing or low-quality calls (marked as "LowQual" in the original archaic VCF files) in one of the archaic samples were excluded during the merging procedure. We kept only sites that had high-quality variant calls in at least 99% of samples in the combined modern/archaic dataset. Applying this 99% call-rate filter yielded a total of 52,443,217 SNPs. However, we removed sites within segmental duplications, repeats, and low complexity regions, thus retaining 49,374,343 SNPs. These masks were downloaded from the UCSC and Broad Institute genome resources (web resources). Phasing was performed with Eagle v2.4.[19] Because our final dataset included complete genomes from very diverse human populations together with a large number of local West Island Southeast Asian and Papuan groups, we did not use any reference datasets to avoid potential phasing bias.

Genotype array data processing

Approximately 1 μg of DNA from each of 42 individuals were sent to Macrogen for genotyping on the Illumina Infinium Omni2.5-8 v1.3 BeadChip array. Samples were processed according to the manufacturer's instructions. Array data was processed in PLINK v1.9.[20] The average missing rate per person in the raw dataset was around 0.45% (min 0.27%, max 2.5%); 2,194,297 autosomal positions were kept after excluding SNPs with more than 5% of missing data.

Array data were imputed with Beagle v5.1[21] with complete genome sequences as a reference. Two imputation reference panels were generated containing both published[6] and unpublished data. For the imputation of 18 Mentawai samples, we applied a reference panel that included 97 complete genome sequences from western Indonesia (Bali, Borneo, Java, Mentawai, Nias, Sulawesi, and Sumatra), the Philippines, and Taiwan. For the imputation of 24 Korowai and Sumba samples, we applied a reference panel made of 249 complete genomes sequence from eastern Indonesia (Alor, Flores, Kei, Lembata, Sumba, and Tanimbar) and Papua (Bougainville, New Britain, New Guinea, including Korowai, and New Ireland).

We filtered variant sites with bcftools and VCFtools[22] to retain only high-quality imputed sites with dosage $R^2 > 0.95$ (estimated squared correlation between the estimated allele dose and the true allele dose, DR2). We extracted these positions from the complete genomes from Korowai, Mentawai, and Sumba ($n = 84$) to produce a new combined SNP set made of imputed and WGS data. We filtered these data to retain SNPs with a proportion of missing data < 0.3 and minor allele frequency (MAF) > 0.05, which resulted in 4,077,164 variants. Imputed genotypes were further filtered to retain genotypes with genotype probability (GP) > 0.90.

RNA sequencing and data processing

RNA sequencing and data processing were carried out as previously described.[8] Whole blood RNA was collected and extracted with the Tempus Blood RNA tube and Tempus Spin RNA Isolation Kit (Invitrogen). The quality and concentration of all extracted RNA samples were assessed with a Bioanalyzer 2100 (Agilent) and a Qubit device (Life Technologies). We selected samples for sequencing on the basis of their RIN (RNA integrity number) by focusing on villages with at least 10 samples with RIN ≥ 6. Library preparation was performed by Macrogen (South Korea) with 750 ng of RNA and the Globin-Zero Gold rRNA Removal Kit (Illumina) according to the manufacturer's instructions. Samples were

sequenced with a 100 bp paired-end configuration on an Illumina HiSeq 2500 to an average depth of 30 million read pairs per individual in three batches (Table S2).

FASTQ read files underwent quality control with FastQC v0.11.5 (web resources), and leading and trailing bases below a Phred score of 20 were removed with Trimmomatic v0.36.[23] Reads were aligned to the human genome (GRCh38 Ensembl release 90, web resources) with STAR v2.5.3a[24] and a two-pass alignment mode. Read counts were quantified with featureCounts v1.5.3[25] against a subset of GENCODE basic (release 27) annotations[26] (web resources) that included only transcripts with support levels 1–3. Coordinates were converted to hg19 with the R package liftOver v1.8.0 (web resources). Gene expression data were filtered to retain 12,539 genes with FPKM (fragments per kilobase of transcript per million mapped reads) > 0.1 and a read count of >6 in at least 50 samples. The distributions of FPKM in each sample and gene were transformed into the quantiles of the standard normal distribution.

DNA methylation data processing

1 μg of DNA from each sample was shipped to Macrogen, bisulfite-converted, and hybridized to Illumina EPIC BeadChips according to the manufacturer's instructions. We randomized samples with respect to village and island across two array batches, and three samples were processed on both batches to control for technical variation (Table S1). DNA methylation data were obtained and processed as previously described[8] with *minfi* v1.30.0[27] We combined and preprocessed the two arrays to correct for array background signal. Signal strength across all probes was evaluated and probes with signal $p < 0.01$ in $>75\%$ of samples were retained. To avoid potential spurious signals due to differences in probe hybridization affinity, we discarded 6,072 probes overlapping known SNPs segregating in any of the study populations based on previously published genotype data.[6] The final number of probes retained was 859,404. Subset-quantile within array normalization (SWAN) was carried out with the "preprocessSWAN" function.[28] Methylated and unmethylated signals were quantile normalized with *lumi* v2.36.0.[29]

Local ancestry inference

We used ChromoPainter v2[30] (CP) to perform local ancestry (LA) inference and detect Asian and Papuan ancestry in all published and newly generated complete genomes from Korowai ($n = 17$), Mentawai ($n = 38$), and Sumba ($n = 29$). This method relies on phased haplotype data and describes each individual recipient chromosome as a mixture of genetic blocks from the set of predefined donor individuals.

First, East Asian and Papuan reference panels were generated to assign local genomic ancestry in target samples. We selected unadmixed East Asian and Papuan samples by running ADMIXTURE v1.3[31] at $K = 3$ with all available East and Southeast Asian, European, and Papuan samples from the combined WGS dataset. For the East Asian reference panel, we kept only Asian samples ($n = 102$) with less than 0.05% of non-East Asian ancestry. For the Papuan reference panel, we kept only Papuan samples ($n = 63$) with less than 0.05% of non-Papuan ancestry and excluded all Korowai samples. To balance the sample size of the two reference panels, we randomly selected 63 East Asian samples from the unadmixed reference dataset.

Next, we painted each of 84 target genomes individually with the East Asian and Papuan reference panels as donors. We used the following protocol.

(1) We performed the initial CP run with ten expectation-maximization steps to estimate prior copying probabilities for each individual and chromosome separately.
(2) Estimated prior copying probabilities were averaged across the genome for each individual. The main CP run was performed with a recombination scaling constant and global mutation probability from the first step and genome-wide average prior copying probability.
(3) Either East Asian or Papuan ancestry was then assigned to individual SNPs with a probability threshold of 0.85. Unknown ancestry was assigned to SNPs with intermediate copying probability.

Identifying archaic introgression

We defined the high-confidence Denisovan archaic haplotypes as outlined previously[6] but with a larger group of sub-Saharan African individuals (61 sub-Saharan Africans in total, Table S3) For each individual, we started with Denisovan-introgressed haplotypes as inferred by CP, then filtered out those that did not overlap (by $>0.001\%$) the Denisovan-introgressed haplotypes as inferred by a previously published hidden Markov model (HMM),[6] then filtered out those that did not overlap (by $>0.001\%$) archaic introgressed haplotypes inferred by another HMM approach,[32] and finally filtered out any of the remaining haplotypes that did overlap (by $>0.001\%$) Neanderthal-introgressed haplotypes as inferred by CP. We then annotated each SNP found in several target sample groups (i.e., monomorphic SNPs in that group are skipped, as are any that are masked out by the alignability/gap mask) according to how often the reference/alternative (REF/ALT) state appears on an inferred high-confidence Denisovan-introgressed haplotype in that group. This was done for three separate groups: (1) all Korowai individuals, (2) all Korowai individuals and Sumba individuals and those Mentawai individuals who are from the new dataset, and (3) all individuals in the "Papuan" continental group, which includes all Papuans and Melanesians except Baining. We used an analogous process to annotate Neanderthal ancestry SNPs, beginning instead with Neanderthal-introgressed haplotypes inferred by CP before requiring intersection with Neanderthal-introgressed haplotypes inferred by the HMM and archaic haplotypes inferred by $HMM_{Archaic}$ and removing those intersecting CP Denisovan haplotypes.

Accounting for population structure and non-genetic sources of variation in the QTL analyses

Principal-component analysis (PCA) of the genotype data was carried out with the R package *SNPRelate* v1.18.1.[33] We included five genotype principal components (PCs) as covariates in QTL analyses to account for population structure. We used a probabilistic estimation of expression residuals[34] (PEER) to infer hidden sources of variation in expression and methylation data. These latent factors were used as surrogate variables for unknown technical batch effects and included as covariates in the QTL analyses. 29 hidden factors (25% of the number of samples) were included in models, as recommended in Stegle et al. (2012)[34] (for technical details, see Stegle et al. [2010][35]).

eQTL and methylQTL analyses

Variant effects on gene expression and CpG methylation were identified by linear regression as implemented in QTLtools.[36] Genotype, gene expression, and methylation data were available for

115 individuals: 48 Mentawai, 48 Sumba, and 19 Korowai (Tables S1 and S2). Variants within 1 Mb of the gene/CpG under investigation were considered for testing. p values of top associations adjusted for the number of variants tested in *cis* were obtained from 10,000 permutations. We calculated false discovery rate (FDR)-adjusted p values to adjust for multiple phenotypes tested. Significant associations were selected with an FDR-adjusted p value threshold of 0.01. Nominal p values for all sites within the *cis*-window were obtained with the QTLtools nominal pass. QTL power calculations were carried out with the R package *powerEQTL* v0.1.7.[37]

Variant annotation and variant set enrichment analyses

To understand the genomic context of the putative eQTLs and methylQTLs, we annotated top SNPs from the permutation-based analyses and the target CpGs of methylQTLs by using the R package *annotatr* v1.10.0.[38] Genic annotations (1–5 kb upstream of the transcription start site (TSS), the promoter [<1 kb upstream of the TSS], 5′ UTR, first exons, exons, introns, coding sequences [CDS], 3′ UTR, and intergenic regions) were obtained with the *TxDb.Hsapiens.UCSC.hg19.knownGene* R package v3.2.2 (web resources), CpG annotations with the *AnnotationHub* R package v2.16.1 (web resources), and enhancer annotations from FANTOM5.[39]

We tested for the enrichment of the eQTL and methylQTL variants among genomic features by using the *VSE* R package v0.99.[40] A null-distribution was constructed on the basis of 100 matched random variant sets. Consolidated chromatin immunoprecipitation sequencing (ChIP-seq) peaks for histone marks derived from primary mononuclear cells from peripheral blood were downloaded from the NIH Epigenomics Roadmap FTP site.[41] Additionally, annotations for DNaseI hypersensitivity peaks and histone marks for K562 and GM12878 cell lines were downloaded from the ENCODE portal.[42]

We tested for the overrepresentation of the population-specific eGenes among Gene Ontology (GO) terms and canonical pathways by using *clusterProfiler* 3.14.3.[43] We used a background set of all eGenes to test for overrepresentation.

eQTL-methylQTL colocalization analysis

We used a Bayesian test, as implemented in the R package *coloc* v4,[44,45] to assess the probability of colocalization of methylQTL and eQTL signals between 3,057 pairs of CpGs and genes. We used masking to allow for multiple causal loci for each trait. Masking implemented in *coloc* allows for multiple causal variants per trait with the assumption that if multiple causal variants exist for any individual trait, they are in linkage equilibrium. All SNPs independently associated within a dataset were identified with the function "finemap.signals." For the pairs of CpGs and genes with multiple signals, colocalization analysis was performed for each pair of signals, restricting the search space to SNPs not in linkage disequilibrium (LD) with any-but-one of each signal SNP. The p value threshold for calling a signal was set to 1×10^{-6}, and the maximum r^2 between two SNPs for them to be considered independent was 0.01.

Pairs with the posterior probability for a common causal variant (CCV) > 0.8 and the ratio of the posterior probability for a CCV and different causal variants (DCVs) CCV/DCV > 5 were considered to show strong evidence of colocalization. As the posterior probability for colocalization is dependent on the prior probability, we used the coloc post hoc sensitivity analysis to determine the range of prior probabilities (1.0×10^{-8} to 1.0×10^{-4}) for which colocalization is supported. Pairs passing the colocalization threshold with a range of ppCCV values from $<1.0 \times 10^{-6}$ to 1.0×10^{-4} (lower bound of ppCCV below 1.0×10^{-6}) were considered as showing robust support for colocalization.

eQTL sharing with European eQTLs

Similarly to eQTL-methylQTL colocalization, we used *coloc* v4 to test for colocalization between 3,300 permutation-based eQTLs detected here with an FDR-adjusted $p < 0.10$ and three European whole blood eQTL studies: GTEx[46] ($n = 670$), the Estonian Biobank cohort[47] ($n = 491$), and Twins UK[48] ($n = 384$). The European eQTL summary statistics were obtained from the EBI eQTL catalog.[49] The methods used to call the eQTLs in the EBI eQTL catalog are comparable to the methods used in this study. Out of the 3,300 genes selected for testing, 3,049 were present in the European data and had shared variants with the Indonesian data. We identified colocalized genes with the threshold CCV > 0.8 and a ratio CCV/DCV > 5. To identify genes that do not show support for colocalization even with a relaxed threshold, we used a threshold of CCV > 0.5 and CCV/DCV > 2.

To compare the alternative and minor allele frequencies of eSNPs between populations, European genotype data were obtained from the 1000 Genomes dataset.[50] After subsetting the VCFs for the samples belonging to the European superpopulation, alternative allele counts and frequencies were recalculated with VCFtools.[22] Minor allele frequencies in Indonesia and Europe were calculated in relation to the minor allele in the Indonesian data.

Estimating and testing for differences in effect sizes between populations

We used multivariate adaptive shrinkage as implemented in the R package *mashr*[51] to more reliably estimate effect sizes and to identify shared and population-specific eQTLs. The model was fit with both data-driven and canonical covariances. The data-driven covariance matrix was constructed by identifying strong signals based on a significance threshold of 0.05, by obtaining the initial data-driven covariance matrix for the first two PCs of the strong signals, and then applying the built-in extreme deconvolution algorithm. To facilitate computational limitations, we fit the model by using a random subset of 100,000 SNP-gene pairs. For the calculation of pairwise sharing of eQTLs, an eQTL was considered shared between datasets if the effects are the same sign and within a factor of 0.5.

Colocalization with blood trait GWAS loci

To connect the QTLs detected here to blood traits, we tested for colocalization between the FDR-significant permutation-based QTLs (FDR-$p < 0.1$ eQTLs and FDR-$p < 0.01$ methylQTLs) and 36 hematological traits by using genome-wide summary statistics from Astle et al.[52] GWAS summary statistics were downloaded from the GWAS catalog.[53] As no LD information was available, these colocalization analyses were carried out without allowing for multiple causal variants. Similarly, eQTL-GWAS colocalization analysis was carried out with the European datasets.

Selection scan

We performed a selection scan using a haplotype-based statistic (number of segregating sites by length, nSL),[54] as implemented in Selscan v1.2.0.[55] This test identifies ongoing positive selection in the genome by looking for the tracts of extended haplotype

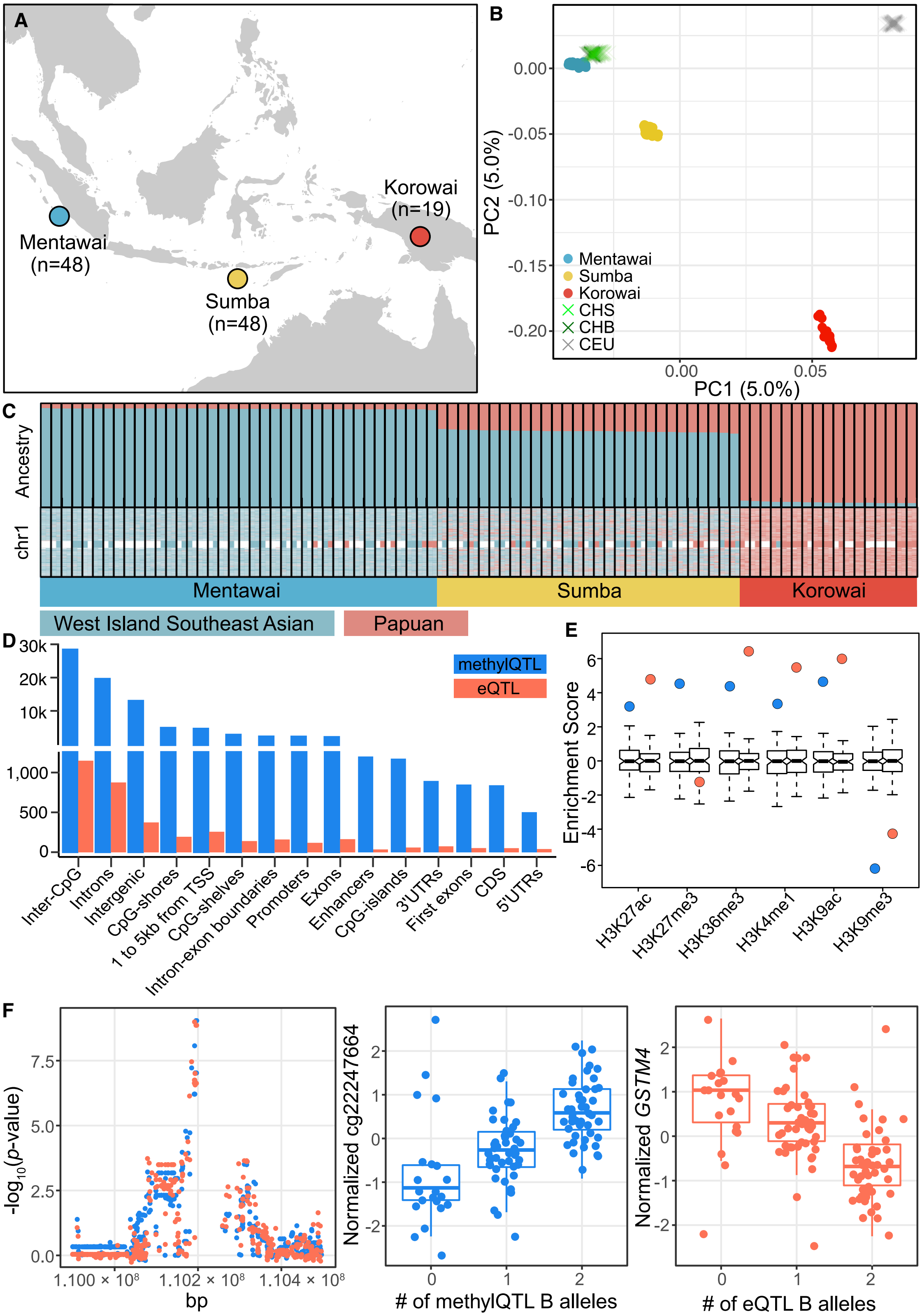

Figure 1. Genetic ancestry and QTL features across 115 Indonesian individuals
(A) Map of the sampling locations of the three study populations: Mentawai, blue; Sumba, yellow; Korowai, red. The numbers of samples used in the QTL analyses are indicated.
(B) PCA of genotype data from study samples as well as Han Chinese from Beijing (CHB), Southern Han Chinese (CHS), and individuals of European ancestry (CEU) from the 1000 Genomes project.

(legend continued on next page)

homozygosity and is capable of identifying both sweeps from standing variation and incomplete sweeps. To identify the traces of positive selection in three target populations, we used our combined dataset of WGS and imputed genotyping array data represented by approximately 4M SNPs. The following Selscan parameters were used: the maximum allowed gap between loci of 50 kb, the gap scale parameter of 5 kb, and the maximum extent of haplotype homozygosity decay curve of 1,333 loci (approximately 1 Mb given the obtained SNP density). Raw nSL results were normalized with Selscan's norm package in 50 kb non-overlapping genomic windows with ten allele frequency bins. Windows with less than 21 SNPs were discarded. The proportion of absolute nSL scores > 2 in each 50 kb genomic window was used as a test statistic. Windows with a proportion of SNPs with an absolute nSL > 2 of 30% were considered to be outliers and showing evidence of past positive selection.

To identify regulatory loci under positive selection, we used a colocalization-based method to detect shared signals between the QTL p values and nSL values. We calculated empirical p values for the nSL values by using an outlier approach by sorting all the scores genome-wide and then dividing the rank by the total number of values in the distribution.

Identifying eQTL effects driven by local ancestry

We calculated the variance explained by modern LA in the genotype of each significant (FDR-p < 0.01) permutation-based eQTL and methylQTL as previously described.[56] For each eVariant and methylVariant, we fit the linear model $V = \alpha \times PAP + \beta$, where V is the genotype vector (number of QTL B alleles) and PAP is the LA covariate, representing the number of alleles assigned to the Papuan population. This analysis was carried out with the 73 WGS (30 Mentawai, 29 Sumba, 14 Korowai) samples included in the LA inference. Variants with an absolute $R^2 > 0.7$ were considered to exhibit a high correlation with LA. Similarly, we calculated the variance explained by archaic Denisovan and Neanderthal ancestry.

Results

Genetic determinants of gene expression and CpG methylation levels in Indonesia

To contextualize the genetic diversity in our dataset, we began by clustering the 115 Indonesian samples (Figure 1A) through PCA of genotype data, along with European and Han Chinese samples from the 1000 Genomes project. The first two PCs clearly separate the three study populations (Figure 1B). The Mentawai, of West Island Southeast Asian ancestry, cluster closest to mainland Chinese populations, whereas the Korowai, representative of Papuan ancestry (not well represented in existing public datasets), form a distinct cluster from all other populations. Individuals from Sumba—a mixture of the two ancestries—fall between Mentawai and Korowai, as expected.[8]

We genotyped our samples by using two separate platforms, whole-genome sequencing (WGS, n = 73) and the Illumina Omni 2.5M genotyping array (n = 42; material and methods, Table S1). Using only the complete genome sequences, we inferred patterns of global and local ancestry (LA) and archaic introgression across the three populations. On average, the proportion of the genome for which we can make a confident ancestry assignment is 80% for Mentawai, 71% for Sumba, and 85% for Korowai. The average proportion of ancestry-called individual haploid genomes assigned as Papuan is 5.3% in Mentawai, 26.8% in Sumba, and 95.0% in Korowai (Figure 1C). In addition, we were able to identify Denisovan-introgressed haplotypes covering, on average, 0.13%, 0.48%, and 1.44%, of each haploid genome in Mentawai, Sumba, and Korowai, respectively, consistent with a previous study[6] showing a high frequency of Denisovan sequence in Korowai (Figure S1). Proportions of inferred Papuan ancestry and Denisovan introgression are highly correlated (Pearson's $r = 0.995$, Figure S1). Further, we identified Neanderthal-introgressed haplotypes covering on average 1.08%, 1.19%, and 1.40% of each haploid genome from the three study populations, raising the possibility that either archaic ancestry source has made contributions to gene regulatory architecture in these populations.

To identify genetic variants associated with changes in expression (eQTL) and methylation (methylQTL) levels, we used a linear regression-based approach (material and methods). At an FDR of 0.01, we detect a total of 1,975 significant *cis*-eQTLs (Data S1) and 48,014 *cis*-methylQTLs (Data S2). As expected, the majority of QTLs are located in non-coding parts of the genome, enriched among transcriptionally active histone marks and accessible chromatin, and mostly depleted from marks associated with heterochromatin and repression of transcription across three blood cell lines (Figures 1D and 1E, Figure S2). We then tested for colocalization between 4,639 pairs of CpGs and genes that potentially harbor a common causal variant by using a Bayesian approach (material and methods, supplemental note 1) to better understand how genetic regulation of methylation levels contributes to the regulation of gene expression. Over a wide range of

(C) Global and local ancestry across 73 Indonesian individuals (bars) with available WGS data. The top plot shows the average proportion of West Island Southeast Asian and Papuan ancestry genome wide. The bottom plot shows patterns of local ancestry across the two haplotypes in each individual in chr1.
(D) Genomic locations of eQTLs (orange) and methylQTLs (blue). Each QTL can have multiple annotations.
(E) Enrichment of eQTLs (orange) and methylQTLs (blue) among histone marks derived from primary mononuclear cells from peripheral blood in the Epigenomics Roadmap project compared to a null-distribution of 100 matched random variant sets.
(F) An example of a colocalized eQTL-methylQTL pair exhibiting an opposing effect direction on the target trait. The left-side plot shows the $-\log_{10}$(p values) of the associations between variants in *cis* and *GSTM4* expression (orange) or cg22247664 methylation (blue). The middle plot shows the relationship between the top-SNP genotype and cg22247664 methylation, and the right-side shows the relationship between the top-SNP genotype and *GSTM4* expression.

prior probabilities, 720 (15.5%) of the tested pairs show robust support for a common causal variant (material and methods, Figure S3, Data S4), corresponding to 621 unique CpGs and 222 unique genes. As expected, CpGs located on promoters are more likely to show an opposite direction of effect with the gene than CpGs located outside regulatory regions (Fisher's test $p = 3.835 \times 10^{-6}$, Figure 1F, supplemental note 1, Figure S4); additionally, we had previously identified 80 (36.0%) of the 222 genes as showing a negative correlation between expression and promoter methylation levels.[8]

Population specificity of Indonesian eQTLs

The bulk of eQTL studies to date have been carried out in European populations. To better understand the impact of ancestry on the genetic architecture of gene regulation, we compared eQTLs detected here with those identified in three comparable mostly European datasets with publicly available genome-wide summary statistics: GTEx[46] (n = 670), the Estonian Biobank cohort[47] (n = 491), and Twins UK[48] (n = 384). While 996 (9.8%) of the 10,214 unique eGenes were shared across cohorts, the same number of eGenes were detected in all European cohorts but not in Indonesia (Figure 2A). Furthermore, 698 (6.8%) were only detected in the Indonesian data. After relaxing our FDR threshold to $p < 0.10$ to account for differences in power, we tested 3,300 Indonesian eGenes for eQTL colocalization with any of the European datasets. On average, 26.9% of tested genes showed some evidence of colocalization and 6.71% showed robust support across a wide range of prior probabilities with each European dataset (Table S4). In total, 1,177 (35.7%) of tested genes showed some evidence of colocalization and 318 (9.6%) robust support for colocalization with a wide range of prior probabilities for a common causal variant with at least one European dataset. Of these, 105 (33.0%) genes (Table S4), including Ras suppressor *RSU1* (Figure 2E, Figure S5), showed robust support for colocalization between Indonesia and all European datasets. In contrast, we found much higher levels of colocalization when comparing between European datasets at a similarly relaxed FDR: on average, 46.3% of tested genes colocalized across at least one pair of datasets with some support and 17.8% with robust support (Table S4). These results suggest a true difference in eQTL architecture between the Indonesian and European data. Indeed, 1,081 (32.8%) of genes exhibited no evidence of colocalization between Indonesia and any European dataset, even with relaxed thresholds (material and methods). We followed up on these results by using a multivariate adaptive shrinkage model (material and methods) that enables joint analysis of all the datasets simultaneously and is geared toward more accurately estimating effect sizes and identifying shared and specific effects. Concordant with the colocalization results above, we found eQTL effects were largely shared between the European datasets (Figure 2B). We also identified 2,411 Indonesia-specific eSNP-eGene pairs, corresponding to a total of 1,599 eGenes. To generate a robust set of Indonesia-specific eQTLs, we intersected the results from the colocalization and multivariate analyses and identified 245 genes that harbored Indonesia-specific eQTLs and had no evidence of colocalization with European eQTLs. It should be noted that these Indonesia-specific eQTLs are likely to be present in some unsurveyed populations and thus specific to particular populations and not only Indonesia. As our comparison using the available datasets is between European and Indonesian eQTLs, we are calling this set of eQTLs Indonesia specific in the current study.

To identify the attributes of Indonesia-specific eQTLs, we compared these 245 genes to the set of 105 genes with robust support for colocalization between Indonesia and all European datasets. Although there is no overall enrichment for GO or KEGG terms among Indonesian-specific eGenes, there are clinically relevant genes in this set (Table S5), including the *NRAS* proto-oncogene (Figure 2F, Figure S5). Concordant with previous reports, eQTLs that are shared between populations exhibit larger effect sizes than other eQTLs (t test $p < 2.2 \times 10^{-16}$), and most (97%–100%) shared eQTLs show the same direction of effect in both populations[57] (Figure S6). Indonesia-specific eQTLs exhibit significantly larger effect sizes in Indonesia than in the European datasets, while colocalized eQTLs show no statistically significant differences in effect sizes between datasets, as expected (Figure 2D). We hypothesized that differences in genetic trait architecture may underlie these population-specific eQTLs. Indeed, when we compared the MAF of eSNPs between Indonesians and Europeans, we found no significant difference among the colocalized eQTLs but a significantly higher MAF in Indonesians for the Indonesian-specific eQTLs (t test; mean MAF in Indonesia 0.26; in the 1000 Genomes European super population 0.22; $p = 0.0099$), suggesting population differences in haplotype structure contribute to our observation. However, they alone were insufficient to explain the entirety of Indonesia-specific eQTLs. Thus, we also examined the role of gene expression levels—i.e., whether a gene is highly expressed in Indonesian samples but expressed at low levels or not at all in European ones. Indeed, as with MAF, we observed no significant difference in expression levels of colocalized eGenes across the datasets, but there was a significant increase in expression of the Indonesia-specific eGenes (t test; mean $\log_2$(TPM) in Indonesia 3.5, in GTEx whole blood 2.6, $p < 2.8 \times 10^{-12}$, Figure S7). Interestingly, there were a number of Indonesia-specific eQTLs where little or no difference in MAF or gene expression was observed, highlighting the need for a deeper mechanistic investigation of these loci (Figure S8).

While some of the population specificity we detect may be attributable to limited statistical power across all datasets, our findings illustrate the value of performing analyses on diverse populations to achieve a comprehensive understanding of the genetic regulation of molecular traits. Importantly, of all datasets under consideration,

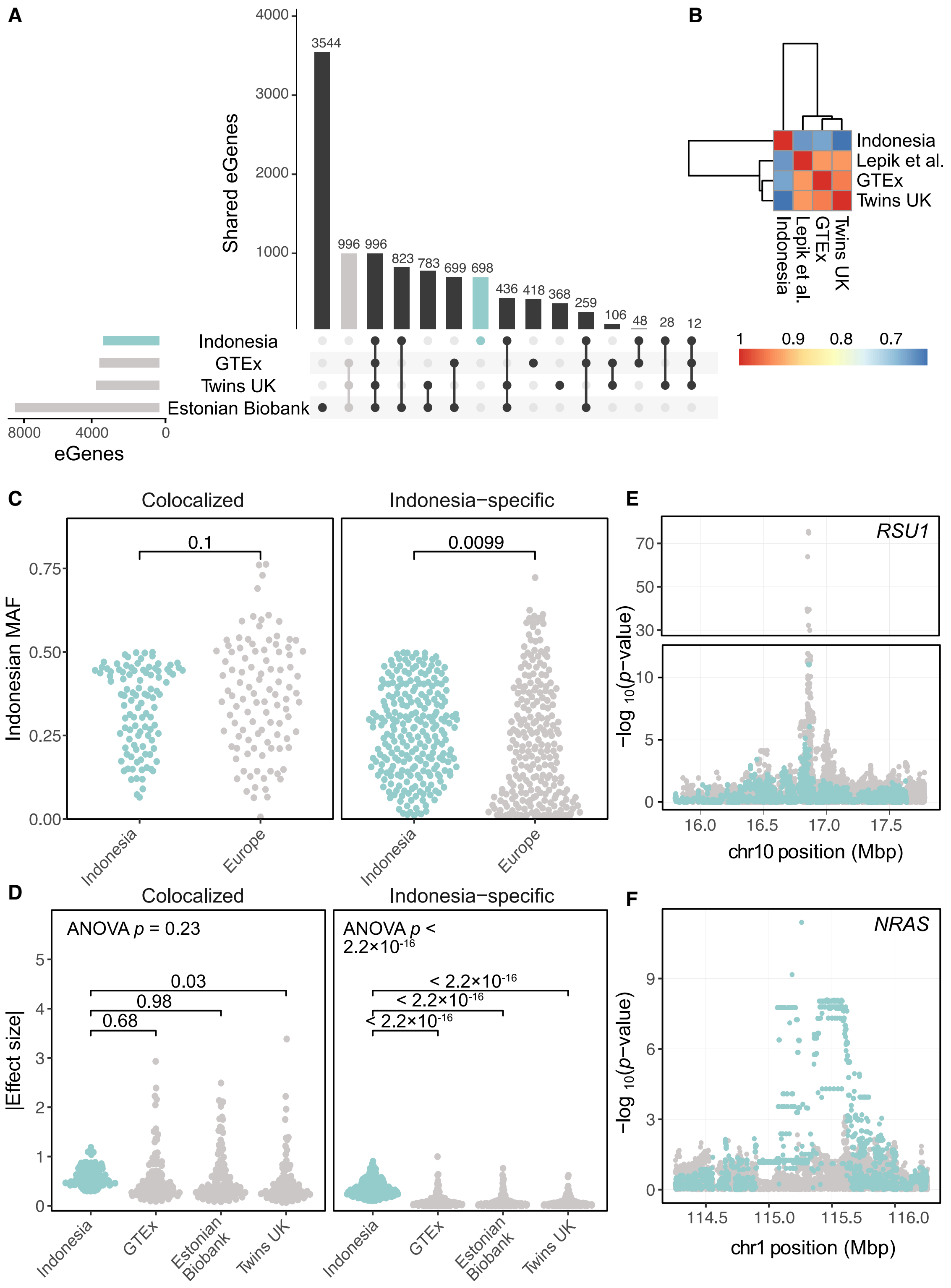

Figure 2. Sharing of eQTLs between Indonesian and European populations
(A) Overlap of permutation-significant (FDR-p < 0.1) eGenes between Indonesia and three European eQTL cohorts.
(B) Pairwise sharing of eQTLs between the datasets across all tested SNP-gene pairs.
(C) Minor allele frequencies of shared and Indonesia-specific eSNPs in Indonesia and the 1000 Genomes European super-population. MAFs are reported relative to the minor allele in Indonesia.
(D) Absolute effect sizes of the shared and Indonesia-specific eQTLs. In (C) and (D), ANOVA p values and t test p values between Indonesia and the European datasets are indicated.
(E) An example of a colocalized gene, *RSU1*.
(F) An example of an Indonesia-specific eQTL for the gene *NRAS*. In (E) and (F), $-\log_{10}$(p values) for Indonesia are indicated in blue and for the European datasets are indicated in gray.

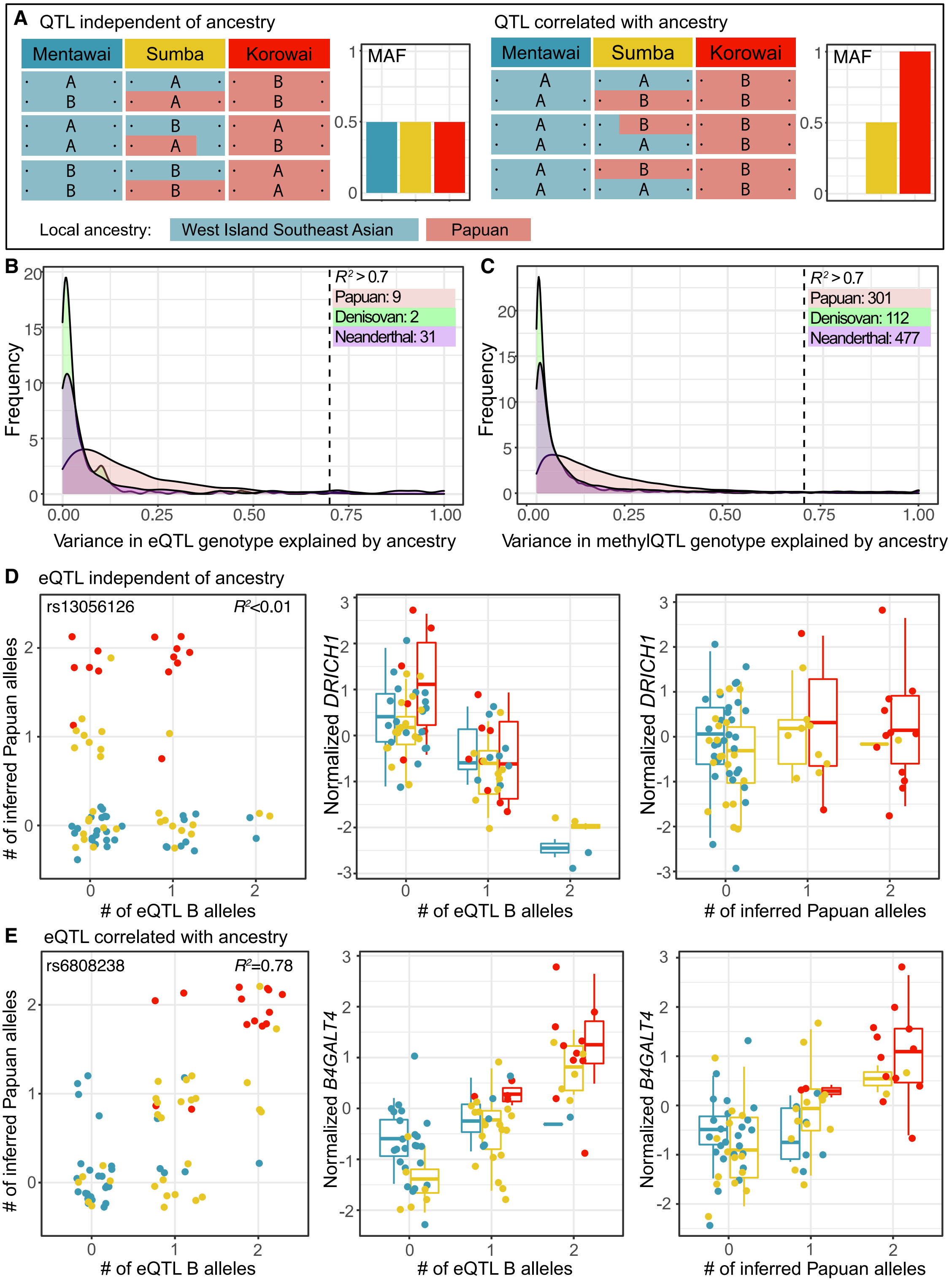

Figure 3. Integrating local ancestry inference at regulatory loci to detect QTLs driven by ancestry and archaic introgression
(A) Schematic illustrations of variation in QTL genotype (A = major allele, B = minor allele) and local ancestry are shown across the two haplotypes in three individuals in three populations. In the first example, QTL genotype variation is independent of local ancestry and allele frequencies are equal between populations. In the second example, QTL B allele closely segregates with the ancestry informative marker and allele frequencies differ between populations. There is an expected correlation between the genotype and the molecular trait, as well as inferred ancestry and the trait.
(B and C) Linear regression between the numbers of QTL B alleles and numbers of inferred Papuan, Denisovan, and Neanderthal alleles reveal subsets of (B) eQTLs and (C) methylQTLs largely driven by modern LA and archaic introgression. The numbers of QTLs exceeding the R^2 threshold of 0.7 are indicated.
(D) An example of an eQTL independent of modern LA.

(legend continued on next page)

the Indonesian one is the smallest, making our inability to replicate Indonesian eQTLs in European studies more likely to be biologically meaningful and clear candidates for functional follow-up.

Subsets of expression and methylation QTLs are largely driven by modern local ancestry and archaic introgression

In addition to differences between Indonesians and Europeans, we sought to understand the extent to which the two distinct sources of LA in modern Indonesians, as well as introgression from archaic hominins, have impacted gene regulatory architecture. We examined the haplotype background on which our QTLs occur and asked whether there was a relationship between the inferred ancestral source of the genotype and expression/methylation levels (material and methods, Figures 3A, 3D, and 3E). We find nine, two, and 31 instances where variance in eQTL genotype is largely driven ($R^2 > 0.7$) by modern LA, archaic Denisovan introgression, and archaic Neanderthal introgression, respectively (Figure 3B, Data S5, Data S6, Data S7), directly linking ancestry-informative alleles to expression differences between individuals. Similarly, we find 301, 112, and 477 instances where the methylQTL genotype is driven by modern LA, Denisovan introgression, and Neanderthal introgression, respectively (Figure 3C, Data S8, Data S9, Data S10). In total, 2.1% of eQTLs and 2.29% of methylQTLs are driven by modern LA or archaic introgression; given the unbalanced representation of the two ancestries in our dataset (Figure S1) this number represents only a lower boundary, and the true number is likely to be much greater.

Of the nine and 373 unique Papuan-driven QTL target genes and CpGs, we had previously identified seven (77.8%) and 270 (72.4%) as differentially expressed/methylated in at least one of the pairwise comparisons between the three study populations.[8] Further, 42 out of the 122 (34.4%) Denisovan-driven methylQTL targets were differentially methylated and seven (22.6%) and 149 (25.6%) of the Neanderthal-driven eQTL and methylQTL targets are differentially expressed/methylated. However, despite multiple lines of evidence suggesting that some introgressed Neanderthal and Denisovan alleles have been positively selected for in human populations,[58–60] we were unable to identify evidence of recent positive selection among these loci (supplemental note 3, Table S6, Table S7). Altogether, our findings highlight the potential for local sources of genetic ancestry, whether modern or archaic, to variably impact gene expression architecture across populations and again emphasize the importance of truly diverse sampling.

Connecting regulatory variants to complex traits

Differences in genetic architecture between populations, including differences in allele frequencies and patterns of LD, are known to limit the transferability of GWASs and polygenic risk scores across populations.[1,4] Thus, we sought to examine how the differences in QTL architecture between Indonesian and European populations propagate through to the genetic underpinnings of complex traits.

First, using the same Bayesian methodology as above, we tested for colocalization between the significant Indonesian QTLs and 36 hematological traits by using genome-wide summary statistics from a GWAS on 173,480 participants of European ancestry.[52] We detected 30 (1.5%) and 614 (1.3%) unique eGenes and methylCpGs, respectively, that colocalize with at least one trait (Table S8); in total, we identified 78 significant trait-eGene pairs. The genes and CpGs colocalized with the most unique traits were *ITGA4* and cg18815117, colocalizing with ten and 16 traits, respectively. *ITGA4* has been previously implicated in blood trait GWASs across diverse populations.[61–65] The CpG cg18815117 is located in the body of *CRHR1*, an important regulator of the hypothalamic–pituitary–adrenal (HPA) axis. Epigenetic changes in the body and promoter of *CRHR1* have been found to be highly predictive of major depressive disorder and panic disorder in some cohorts.[66–68]

Next, we repeated the GWAS colocalization analysis, this time by using the three European eQTL datasets alone. Compared to the 30 eGenes that colocalized with at least one GWAS trait, here we found an average of 55 unique eGenes colocalizing with at least one trait (45 in GTEx, 48 in TwinsUK, and 77 in the Estonian Biobank). Focusing on pairs of colocalized GWAS-eQTLs—rather than unique eGenes—we found that 72 (28%) of the 257 unique significant trait-gene pairs detected across all datasets were shared across all three European datasets (Figure 4A) and an average of 25.7% of colocalized genes were shared between datasets for a given trait (Table S9). Furthermore, 31 GWAS-eQTL pairs (12%) were shared between all three European datasets and Indonesia. The GWAS-eQTLs shared between Indonesia and Europe show robust support for eQTL colocalization, indicative of a shared genetic architecture underlying the regulation of these genes and the biological association identified by GWAS (Figure 4B). Among the shared GWAS-eQTLs, we identify *SLC12A7*, which colocalized with red blood cell distribution width, a biomarker representing the variability in the size of circulating erythrocytes (Figure 4C). Variants annotated with *SLC12A7* have previously been uncovered in GWASs on this and other datasets.[52,69,70]

In addition to these population-shared GWAS-eQTLs, we also identified population-specific colocalized pairs of

(E) An example of an eQTL highly correlated with modern LA. In (D) and (E), the leftmost plot shows the correlation between the number of inferred Papuan alleles and eQTL B alleles. rs ID and R^2 are indicated. The middle plot shows the effect of the eQTL B allele dosage on the normalized expression level of the target gene. The rightmost plot shows the effect of the inferred Papuan allele dosage on the target gene.

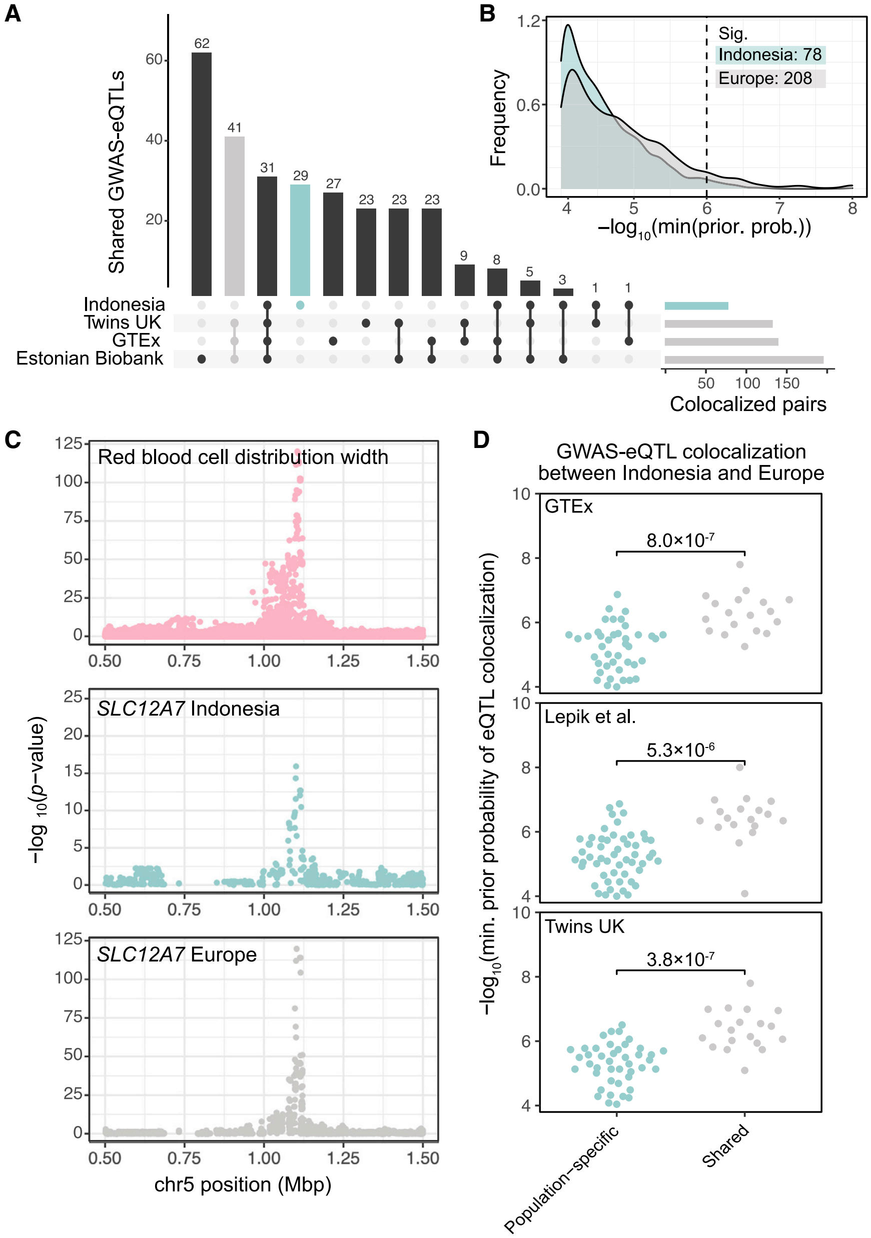

Figure 4. GWAS colocalization with eQTLs from diverse populations identifies shared and population-specific variant-gene-trait associations

(A) Overlap of colocalized trait-gene pairs. The horizontal bar plot shows the numbers of significant colocalized pairs for each dataset. The dot plot shows the intersections and the vertical bar plot shows the numbers of shared trait-gene pairs for each intersection.

(B) European GWAS shows more colocalization with European eQTLs than Indonesian eQTLs. The x axis shows the $-\log_{10}$ of the lower bound of the prior probability of colocalization where the gene passes the colocalization threshold, and larger values indicate a more robust support for eQTL colocalization. The minimum prior probability threshold of 1.0×10^{-6} for robust colocalization is indicated.

(C) An example of a GWAS-eQTL significantly colocalized across Indonesia and all European datasets. $-\log_{10}$(p values) for red blood cell component distribution width (top), *SLC12A7* eQTLs in Indonesia (middle), and *SLC12A7* eQTLs in the three European datasets (bottom) are shown.

(D) Population-specific GWAS-colocalized eGenes are less likely to show eQTL colocalization between Indonesia and Europe than shared GWAS-genes. The y axis shows the $-\log_{10}$ of the lower bound of the prior probability of colocalization where the gene passes the colocalization threshold, and larger values indicate more robust support for eQTL colocalization.

GWAS-eQTLs. Overall, European eQTLs show more evidence for colocalization with GWAS traits than Indonesian eQTLs (Figures 4A and 4B). Indeed, 208 (80.9%) of the 257 unique eGene-trait pairs that were detected across at least one of the European datasets could not be replicated in Indonesia. Intriguingly, although the GWAS was performed in a predominantly European sample, 29 GWAS-eQTL pairs colocalized exclusively in the Indonesian data and not in any of the European datasets (Figure 4A). Out of these, eight were not colocalized in any of the European datasets even with a relaxed significance threshold (see material and methods). Reassuringly, these population-specific GWAS genes show low support for eQTL colocalization (Figure 4D), again strongly arguing for differences in the genetic architecture underlying gene regulation, while simultaneously supporting the importance of the genes in question in contributing to the overall trait. The striking difference in GWAS colocalization between European and Indonesian eQTLs reflects the poor transferability of genetic association studies between populations.

Finally, we sought to identify local ancestry or archaic introgression-driven QTLs that are associated with hematological traits. Among the GWAS-colocalizing QTLs were a Papuan-driven methylQTL that colocalizes with hemoglobin measurements, and notably, four Denisovan-associated methylQTLs that colocalize with platelet count. We further examined these four methylQTLs to gain insight into possible mechanisms underlying the connection with platelet count (supplemental note 4, Table S10). All four target CpGs are located near the *HLA* superlocus. While these methylQTLs do not significantly colocalize with eQTLs in our data, all four methylSNPs are nominally associated with the expression of the nearby gene *ZFP57* (lowest p value $= 5.85 \times 10^{-6}$). *ZFP57* is a transcriptional regulator known to have an important role in DNA methylation, epigenetic regulation, and imprinting during development.[71] Expression of *ZFP57* is dependent

on underlying genetic variation, and while the biology of *ZFP57* in adults is not well studied, it has been implicated as the causal gene connecting some GWAS variants to cancer and HIV/AIDS progression.[72] As above, the geographically restricted ancestry of the methylSNPs and their linkage structure suggests that the regulatory interactions may not be fully shared between populations. Further GWASs and functional studies on diverse populations are needed for the fine-mapping of causal variants underlying gene regulation and complex traits.

Discussion

Indonesia is the world's fourth most populous country and a region that has been vastly understudied, but it is also one that is undergoing a rapid demographic and lifestyle shift giving rise to an expanding middle class and where non-infectious, complex diseases are already contributing substantially to mortality and morbidity. As is happening elsewhere in the Global South, this transition accelerates the need to understand the molecular underpinnings of complex disease, and in this context, our study adds to a growing literature demonstrating the importance of characterizing functional genomics within traditionally understudied populations.[73,74]

We have explored the degree to which functional variation differs between Europeans and Indonesians, and more broadly, the difficult problem of translating eQTL knowledge across populations. Focusing largely on a set of eQTLs that had strong evidence of being colocalized between populations and a set of eQTLs with strong evidence of being specific to Indonesia, we were able to examine potential drivers of population specificity. At least some portion of the population-specific effects we observe are explained by population-specific genomic architecture at *cis*-regulatory regions. The future identification of such eQTLs is fully contingent on performing large-scale QTL studies in underrepresented populations, and our work demonstrates the value of such approaches. Other drivers of population differences in architecture are likely to include *trans* effects (themselves most likely driven by population-specific *cis* effects),[75] as well as environmental differences between populations.[76] The identification of 245 putative population-specific eQTLs—a number of which implicate genes involved with biological processes such as immunity and cancer progression—coupled with the insights gained from exploring the genetic underpinnings of these eQTLs advances our understanding of the genetic architecture of gene regulation. In the future, the collection of multimodal data and mapping of QTLs across tissues and diverse populations can allow a more comprehensive assessment of population specificity and the exact mechanisms underlying population differences in gene regulation. In particular, future surveys across South East Asia can further clarify the genomic and environmental drivers of gene regulatory variation in the area.

In addition to comparisons between Indonesian and European populations, we were able to leverage the unique cline of Asian and Papuan ancestry in Indonesia to identify both QTLs driven by local ancestry or introgression from archaic hominin species. A large proportion of the target genes and CpGs of these LA-driven QTLs were previously identified as differentially expressed or methylated between islands, demonstrating how between-island differences in genetic ancestry can contribute to differences in molecular phenotypes in the region. Notably, these loci, which might *a priori* appear to be prime candidates for local adaptation, showed no evidence of having been targets of positive selection. This suggests that the relationship between trait architecture, demographic history, and adaptation to local pressures such as pathogens is not straightforward. However, our analysis demands a strong correlation between allelic state and ancestry, making our estimates of LA-driven QTLs highly conservative and identifies loci that have evolved under specific scenarios, e.g., drift to high frequency in the Papuan population. Relaxing these demands may lead to the discovery of other regulatory variants that arose within Papua after divergence from Asia. Furthermore, our analysis is limited by the small sample size. Future sample collection efforts across diverse populations will address this limitation. Understanding how complex gene regulatory landscapes and the polygenicity of most traits constrain the action of natural selection is an open challenge in human genomics, one underscored by the difficulty many studies of genome-wide positive selection in humans have encountered in linking evidence of selection at the DNA level to tangible phenotypes.[77,78]

Similarly, the modest overlap of GWAS hits with the population-specific QTLs represents a non-trivial challenge in the field of functional genomics: how do we connect population-specific functional variation to loci associated with complex traits identified in European populations? From a practical perspective, we do not anticipate a robust expansion of traditional GWASs' being carried out in understudied populations. To this end, the field will need to move away from simple intersections of GWASs and QTL hits, which rely upon shared LD structure, and instead integrate genetic variation, GWAS results, context-specific multi-omics (in simulated or actual disease states and in a range of cell types), and robust functional validations to define common sets of regulatory elements that contribute to disease and are shared across populations.

Data and code availability

All genotype data, RNA sequencing reads, and Illumina Epic iDat files are available through the Data Access Committee of the official data repository at the European Genome-phenome Archive (EGA; web resources). Illumina Omni 2.5M genotyping array data are deposited in study EGA: EGAS00001003670 and the whole-genome data are deposited in study EGA: EGAS0000100 3654. The RNA sequencing data are deposited in study EGA:

EGAS00001003671 and the methylation data are deposited in study EGA: EGAS00001003653. Matrices of unfiltered read counts (https://doi.org/10.26188/5d12023f77da8) and M-values (https://doi.org/10.26188/5d13fb401e305) for all samples, including replicates, are freely available on Figshare (web resources). The nominal eQTL statistics for all tested SNP-gene pairs are available as Data S3 and the nominal methylQTL statistics for all SNPs tested for the targets of the permutation-significant methylQTLs are available at Mendeley Data: https://doi.org/10.17632/z7rgf54jd6.2. The code generated during this study is available on GitHub (web resources).

Supplemental information

Supplemental information can be found online at https://doi.org/10.1016/j.ajhg.2021.11.017.

Acknowledgments

We especially thank all of our study participants. This study was supported by a Royal Society of New Zealand Marsden grant 17-MAU-040 to M.P.C. and I.G.R. and by the ASU Center for Evolution and Medicine and the Marcia and Frank Carlucci Charitable Foundation postdoctoral award from The Prevent Cancer Foundation to H.M.N. I.G.R., G.H., and M.M. were partially supported by EU Horizon 2020 grant 810645; M.M. and L.S. were partially supported by European Regional Development Fund projects 2014-2020.4.01.16-0030 and 2014-2020.4.01.15-0012 and by the Estonian Research Council grant PUT (PRG243).

Declaration of interests

The authors declare no competing interests.

Received: October 20, 2021
Accepted: November 16, 2021
Published: December 16, 2021

Web resources

ADMIXTURE v1.3, https://dalexander.github.io/admixture/
AnnotationHub v2.16.1, https://bioconductor.org/packages/release/bioc/html/AnnotationHub.html
annotatr v1.10.0, https://bioconductor.org/packages/release/bioc/html/annotatr.html
bcftools v1.9, https://samtools.github.io/bcftools/
Beagle v5.1, https://faculty.washington.edu/browning/beagle/beagle.html
BLAST, https://blast.ncbi.nlm.nih.gov/Blast.cgi?PAGE_TYPE=BlastSearch
Broad Institute genome annotations, http://software.broadinstitute.org/software/genomestrip/node_ReferenceMetadata.html
ChromoPainter v2, https://people.maths.bris.ac.uk/~madjl/finestructure-old/chromopainter_info.html
clusterProfiler v3.14.3, https://bioconductor.org/packages/release/bioc/html/clusterProfiler.html
coloc v4, https://chr1swallace.github.io/coloc/
Eagle v2.4, https://alkesgroup.broadinstitute.org/Eagle/
ENCODE portal, https://www.encodeproject.org/
eQTL catalog, https://www.ebi.ac.uk/eqtl/
European Genome-Phenome Archive, https://ega-archive.org/
FANTOM5, https://fantom.gsc.riken.jp/data/
FastQC v0.11.5, https://www.bioinformatics.babraham.ac.uk/projects/fastqc/
featureCounts v1.5.3, http://subread.sourceforge.net/
FigShare, https://figshare.com
GATK4, https://gatk.broadinstitute.org/hc
GENCODE release 27, https://www.gencodegenes.org/human/release_27.html
GitHub repository for this publication, https://github.com/heinin/Indonesia_QTL
GRCh37 human reference sequence (hs37d5), ftp://ftp.1000genomes.ebi.ac.uk/vol1/ftp/technical/reference/phase2_reference_assembly_sequence
GRCh38 Ensembl release 90 August 2017, ftp://ftp.ensembl.org/pub/release-90/gtf/homo_sapiens/Homo_sapiens.GRCh38.90.chr.gtf.gz
GWAS catalog, https://www.ebi.ac.uk/gwas/
liftOver v1.8.0, https://www.bioconductor.org/packages/release/workflows/html/liftOver.html
lumi v2.36.0, https://www.bioconductor.org/packages/release/bioc/html/lumi.html
mashr v0.2.50, https://cran.r-project.org/web/packages/mashr/index.html
MatchIt v3.0.2, https://cran.r-project.org/web/packages/MatchIt/
minfi v1.30.0, https://bioconductor.org/packages/release/bioc/html/minfi.html
NIH Epigenomics Roadmap FTP, ftp: //ftp.ncbi.nlm.nih.gov/pub/geo/DATA/roadmapepigenomics/
PEER v1.3, https://github.com/PMBio/peer
PLINK v1.9, https://www.cog-genomics.org/plink/
powerEQTL v0.1.7, https://cran.r-project.org/web/packages/powerEQTL/index.html
QTLtools v1.0, https://qtltools.github.io/qtltools/
Selscan v1.2.0, https://github.com/szpiech/selscan
SNPRelate v1.18.1, https://www.bioconductor.org/packages/release/bioc/html/SNPRelate.html
STAR v2.5.3a, https://github.com/alexdobin/STAR
The Polaris Project, https://github.com/Illumina/Polaris
Trimmomatic v0.36, http://www.usadellab.org/cms/?page=trimmomatic
TxDb.Hsapiens.UCSC.hg19.knownGene v3.2.2, https://bioconductor.org/packages/release/data/annotation/html/TxDb.Hsapiens.UCSC.hg19.knownGene.html
UCSC genome annotations, http://hgdownload.soe.ucsc.edu/goldenPath/hg19/database/genomicSuperDups.txt.gz
Uniquely mappable regions with various kmers, https://bismap.hoffmanlab.org/
VCFtools v0.1.13, https://vcftools.github.io/index.html
VSE v0.99, https://cran.r-project.org/src/contrib/Archive/VSE/

References

1. Duncan, L., Shen, H., Gelaye, B., Meijsen, J., Ressler, K., Feldman, M., Peterson, R., and Domingue, B. (2019). Analysis of polygenic risk score usage and performance in diverse human populations. Nat. Commun. *10*, 3328.
2. Landry, L.G., Ali, N., Williams, D.R., Rehm, H.L., and Bonham, V.L. (2018). Lack Of Diversity In Genomic Databases Is A Barrier To Translating Precision Medicine Research Into Practice. Health Aff. (Millwood) *37*, 780–785.
3. Sirugo, G., Williams, S.M., and Tishkoff, S.A. (2019). The Missing Diversity in Human Genetic Studies. Cell *177*, 1080.

4. Durvasula, A., and Lohmueller, K.E. (2021). Negative selection on complex traits limits phenotype prediction accuracy between populations. Am. J. Hum. Genet. *108*, 620–631.
5. Hudjashov, G., Karafet, T.M., Lawson, D.J., Downey, S., Savina, O., Sudoyo, H., Lansing, J.S., Hammer, M.F., and Cox, M.P. (2017). Complex Patterns of Admixture across the Indonesian Archipelago. Mol. Biol. Evol. *34*, 2439–2452.
6. Jacobs, G.S., Hudjashov, G., Saag, L., Kusuma, P., Darusallam, C.C., Lawson, D.J., Mondal, M., Pagani, L., Ricaut, F.-X., Stoneking, M., et al. (2019). Multiple Deeply Divergent Denisovan Ancestries in Papuans. Cell *177*, 1010–1021.e32.
7. Quintana-Murci, L. (2019). Human Immunology through the Lens of Evolutionary Genetics. Cell *177*, 184–199.
8. Natri, H.M., Bobowik, K.S., Kusuma, P., Crenna Darusallam, C., Jacobs, G.S., Hudjashov, G., Lansing, J.S., Sudoyo, H., Banovich, N.E., Cox, M.P., and Gallego Romero, I. (2020). Genome-wide DNA methylation and gene expression patterns reflect genetic ancestry and environmental differences across the Indonesian archipelago. PLoS Genet. *16*, e1008749.
9. University of Tartu (2018). UT Rocket. https://neic.no/utrocket/.
10. Van der Auwera, G.A., Carneiro, M.O., Hartl, C., Poplin, R., Del Angel, G., Levy-Moonshine, A., Jordan, T., Shakir, K., Roazen, D., Thibault, J., et al. (2013). From FastQ data to high confidence variant calls: the Genome Analysis Toolkit best practices pipeline. Curr. Protoc. Bioinformatics *43*, 11.10.1–11.10.33.
11. Poplin, R., Ruano-Rubio, V., DePristo, M.A., Fennell, T.J., Carneiro, M.O., Van der Auwera, G.A., Kling, D.E., Gauthier, L.D., Levy-Moonshine, A., Roazen, D., et al. (2018). Scaling accurate genetic variant discovery to tens of thousands of samples. bioRxiv. https://doi.org/10.1101/201178.
12. Mallick, S., Li, H., Lipson, M., Mathieson, I., Gymrek, M., Racimo, F., Zhao, M., Chennagiri, N., Nordenfelt, S., Tandon, A., et al. (2016). The Simons Genome Diversity Project: 300 genomes from 142 diverse populations. Nature *538*, 201–206.
13. Malaspinas, A.-S., Westaway, M.C., Muller, C., Sousa, V.C., Lao, O., Alves, I., Bergström, A., Athanasiadis, G., Cheng, J.Y., Crawford, J.E., et al. (2016). A genomic history of Aboriginal Australia. Nature *538*, 207–214.
14. Vernot, B., Tucci, S., Kelso, J., Schraiber, J.G., Wolf, A.B., Gittelman, R.M., Dannemann, M., Grote, S., McCoy, R.C., Norton, H., et al. (2016). Excavating Neandertal and Denisovan DNA from the genomes of Melanesian individuals. Science *352*, 235–239.
15. Lan, T., Lin, H., Zhu, W., Laurent, T.C.A.M., Yang, M., Liu, X., Wang, J., Wang, J., Yang, H., Xu, X., and Guo, X. (2017). Deep whole-genome sequencing of 90 Han Chinese genomes. Gigascience *6*, 1–7.
16. Li, H. (2011). A statistical framework for SNP calling, mutation discovery, association mapping and population genetical parameter estimation from sequencing data. Bioinformatics *27*, 2987–2993.
17. Meyer, M., Kircher, M., Gansauge, M.-T., Li, H., Racimo, F., Mallick, S., Schraiber, J.G., Jay, F., Prüfer, K., de Filippo, C., et al. (2012). A high-coverage genome sequence from an archaic Denisovan individual. Science *338*, 222–226.
18. Prüfer, K., Racimo, F., Patterson, N., Jay, F., Sankararaman, S., Sawyer, S., Heinze, A., Renaud, G., Sudmant, P.H., de Filippo, C., et al. (2014). The complete genome sequence of a Neanderthal from the Altai Mountains. Nature *505*, 43–49.
19. Loh, P.-R., Palamara, P.F., and Price, A.L. (2016). Fast and accurate long-range phasing in a UK Biobank cohort. Nat. Genet. *48*, 811–816.
20. Chang, C.C., Chow, C.C., Tellier, L.C., Vattikuti, S., Purcell, S.M., and Lee, J.J. (2015). Second-generation PLINK: rising to the challenge of larger and richer datasets. Gigascience *4*, *7*.
21. Browning, B.L., Zhou, Y., and Browning, S.R. (2018). A One-Penny Imputed Genome from Next-Generation Reference Panels. Am. J. Hum. Genet. *103*, 338–348.
22. Danecek, P., Auton, A., Abecasis, G., Albers, C.A., Banks, E., DePristo, M.A., Handsaker, R.E., Lunter, G., Marth, G.T., Sherry, S.T., et al. (2011). The variant call format and VCFtools. Bioinformatics *27*, 2156–2158.
23. Bolger, A.M., Lohse, M., and Usadel, B. (2014). Trimmomatic: a flexible trimmer for Illumina sequence data. Bioinformatics *30*, 2114–2120.
24. Dobin, A., Davis, C.A., Schlesinger, F., Drenkow, J., Zaleski, C., Jha, S., Batut, P., Chaisson, M., and Gingeras, T.R. (2013). STAR: ultrafast universal RNA-seq aligner. Bioinformatics *29*, 15–21.
25. Liao, Y., Smyth, G.K., and Shi, W. (2014). featureCounts: an efficient general purpose program for assigning sequence reads to genomic features. Bioinformatics *30*, 923–930.
26. Frankish, A., Diekhans, M., Ferreira, A.-M., Johnson, R., Jungreis, I., Loveland, J., Mudge, J.M., Sisu, C., Wright, J., Armstrong, J., et al. (2019). GENCODE reference annotation for the human and mouse genomes. Nucleic Acids Res. *47* (D1), D766–D773.
27. Aryee, M.J., Jaffe, A.E., Corrada-Bravo, H., Ladd-Acosta, C., Feinberg, A.P., Hansen, K.D., and Irizarry, R.A. (2014). Minfi: a flexible and comprehensive Bioconductor package for the analysis of Infinium DNA methylation microarrays. Bioinformatics *30*, 1363–1369.
28. Maksimovic, J., Gordon, L., and Oshlack, A. (2012). SWAN: Subset-quantile within array normalization for illumina infinium HumanMethylation450 BeadChips. Genome Biol. *13*, R44.
29. Du, P., Kibbe, W.A., and Lin, S.M. (2008). lumi: a pipeline for processing Illumina microarray. Bioinformatics *24*, 1547–1548.
30. Lawson, D.J., Hellenthal, G., Myers, S., and Falush, D. (2012). Inference of population structure using dense haplotype data. PLoS Genet. *8*, e1002453.
31. Alexander, D.H., Novembre, J., and Lange, K. (2009). Fast model-based estimation of ancestry in unrelated individuals. Genome Res. *19*, 1655–1664.
32. Skov, L., Hui, R., Shchur, V., Hobolth, A., Scally, A., Schierup, M.H., and Durbin, R. (2018). Detecting archaic introgression using an unadmixed outgroup. PLoS Genet. *14*, e1007641.
33. Zheng, X., Levine, D., Shen, J., Gogarten, S.M., Laurie, C., and Weir, B.S. (2012). A high-performance computing toolset for relatedness and principal component analysis of SNP data. Bioinformatics *28*, 3326–3328.
34. Stegle, O., Parts, L., Piipari, M., Winn, J., and Durbin, R. (2012). Using probabilistic estimation of expression residuals (PEER) to obtain increased power and interpretability of gene expression analyses. Nat. Protoc. *7*, 500–507.
35. Stegle, O., Parts, L., Durbin, R., and Winn, J. (2010). A Bayesian framework to account for complex non-genetic factors in gene expression levels greatly increases power in eQTL studies. PLoS Comput. Biol. *6*, e1000770.

36. Delaneau, O., Ongen, H., Brown, A.A., Fort, A., Panousis, N.I., and Dermitzakis, E.T. (2017). A complete tool set for molecular QTL discovery and analysis. Nat. Commun. *8*, 15452.
37. Dong, X., Li, X., Chang, T.-W., Scherzer, C.R., Weiss, S.T., and Qiu, W. (2021). powerEQTL: An R package and shiny application for sample size and power calculation of bulk tissue and single-cell eQTL analysis. Bioinformatics, btab385.
38. Cavalcante, R.G., and Sartor, M.A. (2017). annotatr: genomic regions in context. Bioinformatics *33*, 2381–2383.
39. Andersson, R., Gebhard, C., Miguel-Escalada, I., Hoof, I., Bornholdt, J., Boyd, M., Chen, Y., Zhao, X., Schmidl, C., Suzuki, T., et al. (2014). An atlas of active enhancers across human cell types and tissues. Nature *507*, 455–461.
40. Ahmed, M., Sallari, R.C., Guo, H., Moore, J.H., He, H.H., and Lupien, M. (2017). Variant Set Enrichment: an R package to identify disease-associated functional genomic regions. BioData Min. *10*, 9.
41. Chadwick, L.H. (2012). The NIH Roadmap Epigenomics Program data resource. Epigenomics *4*, 317–324.
42. Davis, C.A., Hitz, B.C., Sloan, C.A., Chan, E.T., Davidson, J.M., Gabdank, I., Hilton, J.A., Jain, K., Baymuradov, U.K., Narayanan, A.K., et al. (2018). The Encyclopedia of DNA elements (ENCODE): data portal update. Nucleic Acids Res. *46* (D1), D794–D801.
43. Yu, G., Wang, L.-G., Han, Y., and He, Q.-Y. (2012). clusterProfiler: an R package for comparing biological themes among gene clusters. OMICS *16*, 284–287.
44. Giambartolomei, C., Vukcevic, D., Schadt, E.E., Franke, L., Hingorani, A.D., Wallace, C., and Plagnol, V. (2014). Bayesian test for colocalisation between pairs of genetic association studies using summary statistics. PLoS Genet. *10*, e1004383.
45. Wallace, C. (2020). Eliciting priors and relaxing the single causal variant assumption in colocalisation analyses. PLoS Genet. *16*, e1008720.
46. GTEx Consortium (2020). The GTEx Consortium atlas of genetic regulatory effects across human tissues. Science *369*, 1318–1330.
47. Lepik, K., Annilo, T., Kukuškina, V., Kisand, K., Kutalik, Z., Peterson, P., Peterson, H.; and eQTLGen Consortium (2017). C-reactive protein upregulates the whole blood expression of CD59 - an integrative analysis. PLoS Comput. Biol. *13*, e1005766.
48. Buil, A., Brown, A.A., Lappalainen, T., Viñuela, A., Davies, M.N., Zheng, H.-F., Richards, J.B., Glass, D., Small, K.S., Durbin, R., et al. (2015). Gene-gene and gene-environment interactions detected by transcriptome sequence analysis in twins. Nat. Genet. *47*, 88–91.
49. Kerimov, N., Hayhurst, J.D., Peikova, K., Manning, J.R., Walter, P., Kolberg, L., Samoviča, M., Sakthivel, M.P., Kuzmin, I., Trevanion, S.J., et al. (2021). A compendium of uniformly processed human gene expression and splicing quantitative trait loci. Nat. Genet. *53*, 1290–1299.
50. Auton, A., Brooks, L.D., Durbin, R.M., Garrison, E.P., Kang, H.M., Korbel, J.O., Marchini, J.L., McCarthy, S., McVean, G.A., Abecasis, G.R.; and 1000 Genomes Project Consortium (2015). A global reference for human genetic variation. Nature *526*, 68–74.
51. Urbut, S.M., Wang, G., Carbonetto, P., and Stephens, M. (2019). Flexible statistical methods for estimating and testing effects in genomic studies with multiple conditions. Nat. Genet. *51*, 187–195.
52. Astle, W.J., Elding, H., Jiang, T., Allen, D., Ruklisa, D., Mann, A.L., Mead, D., Bouman, H., Riveros-Mckay, F., Kostadima, M.A., et al. (2016). The Allelic Landscape of Human Blood Cell Trait Variation and Links to Common Complex Disease. Cell *167*, 1415–1429.e19.
53. MacArthur, J., Bowler, E., Cerezo, M., Gil, L., Hall, P., Hastings, E., Junkins, H., McMahon, A., Milano, A., Morales, J., et al. (2017). The new NHGRI-EBI Catalog of published genome-wide association studies (GWAS Catalog). Nucleic Acids Res. *45* (D1), D896–D901.
54. Ferrer-Admetlla, A., Liang, M., Korneliussen, T., and Nielsen, R. (2014). On detecting incomplete soft or hard selective sweeps using haplotype structure. Mol. Biol. Evol. *31*, 1275–1291.
55. Szpiech, Z.A., and Hernandez, R.D. (2014). selscan: an efficient multithreaded program to perform EHH-based scans for positive selection. Mol. Biol. Evol. *31*, 2824–2827.
56. Gay, N.R., Gloudemans, M., Antonio, M.L., Abell, N.S., Balliu, B., Park, Y., Martin, A.R., Musharoff, S., Rao, A.S., Aguet, F., et al. (2020). Impact of admixture and ancestry on eQTL analysis and GWAS colocalization in GTEx. Genome Biol. *21*, 233.
57. Stranger, B.E., Montgomery, S.B., Dimas, A.S., Parts, L., Stegle, O., Ingle, C.E., Sekowska, M., Smith, G.D., Evans, D., Gutierrez-Arcelus, M., et al. (2012). Patterns of cis regulatory variation in diverse human populations. PLoS Genet. *8*, e1002639.
58. Sankararaman, S., Mallick, S., Patterson, N., and Reich, D. (2016). The Combined Landscape of Denisovan and Neanderthal Ancestry in Present-Day Humans. Curr. Biol. *26*, 1241–1247.
59. Vespasiani, D.M., Jacobs, G.S., Cook, L.E., Brucato, N., Leavesley, M., Kinipi, C., Ricaut, F.-X., Cox, M.P., and Romero, I.G. (2021). Denisovan introgression has shaped the immune system of present-day Papuans. bioRxiv. https://doi.org/10.1101/2020.07.09.196444.
60. Gittelman, R.M., Schraiber, J.G., Vernot, B., Mikacenic, C., Wurfel, M.M., and Akey, J.M. (2016). Archaic Hominin Admixture Facilitated Adaptation to Out-of-Africa Environments. Curr. Biol. *26*, 3375–3382.
61. Okada, Y., Hirota, T., Kamatani, Y., Takahashi, A., Ohmiya, H., Kumasaka, N., Higasa, K., Yamaguchi-Kabata, Y., Hosono, N., Nalls, M.A., et al. (2011). Identification of nine novel loci associated with white blood cell subtypes in a Japanese population. PLoS Genet. *7*, e1002067.
62. Crosslin, D.R., McDavid, A., Weston, N., Zheng, X., Hart, E., de Andrade, M., Kullo, I.J., McCarty, C.A., Doheny, K.F., Pugh, E., et al. (2013). Genetic variation associated with circulating monocyte count in the eMERGE Network. Hum. Mol. Genet. *22*, 2119–2127.
63. Jain, D., Hodonsky, C.J., Schick, U.M., Morrison, J.V., Minnerath, S., Brown, L., Schurmann, C., Liu, Y., Auer, P.L., Laurie, C.A., et al. (2017). Genome-wide association of white blood cell counts in Hispanic/Latino Americans: the Hispanic Community Health Study/Study of Latinos. Hum. Mol. Genet. *26*, 1193–1204.
64. Chen, M.-H., Raffield, L.M., Mousas, A., Sakaue, S., Huffman, J.E., Moscati, A., Trivedi, B., Jiang, T., Akbari, P., Vuckovic, D., et al. (2020). Trans-ethnic and Ancestry-Specific Blood-Cell Genetics in 746,667 Individuals from 5 Global Populations. Cell *182*, 1198–1213.e14.
65. Vuckovic, D., Bao, E.L., Akbari, P., Lareau, C.A., Mousas, A., Jiang, T., Chen, M.-H., Raffield, L.M., Tardaguila, M., Huffman, J.E., et al. (2020). The Polygenic and Monogenic Basis of Blood Traits and Diseases. Cell *182*, 1214–1231.e11.

66. Humphreys, K.L., Moore, S.R., Davis, E.G., MacIsaac, J.L., Lin, D.T.S., Kobor, M.S., and Gotlib, I.H. (2019). DNA methylation of HPA-axis genes and the onset of major depressive disorder in adolescent girls: a prospective analysis. Transl. Psychiatry *9*, 245.
67. Schartner, C., Ziegler, C., Schiele, M.A., Kollert, L., Weber, H., Arolt, V., Pauli, P., Zwanzger, P., Reif, A., Deckert, J., et al. (2016). Hypomethylation of corticotropin releasing hormone receptor 1 promoter region: Converging evidence for a role in panic disorder. Eur. Neuropsychopharmacol. *26*, S593.
68. Jokinen, J., Boström, A.E., Dadfar, A., Ciuculete, D.M., Chatzittofis, A., Åsberg, M., and Schiöth, H.B. (2018). Epigenetic Changes in the CRH Gene are Related to Severity of Suicide Attempt and a General Psychiatric Risk Score in Adolescents. EBioMedicine *27*, 123–133.
69. Pilling, L.C., Atkins, J.L., Duff, M.O., Beaumont, R.N., Jones, S.E., Tyrrell, J., Kuo, C.-L., Ruth, K.S., Tuke, M.A., Yaghootkar, H., et al. (2017). Red blood cell distribution width: Genetic evidence for aging pathways in 116,666 volunteers. PLoS ONE *12*, e0185083.
70. Kichaev, G., Bhatia, G., Loh, P.-R., Gazal, S., Burch, K., Freund, M.K., Schoech, A., Pasaniuc, B., and Price, A.L. (2019). Leveraging Polygenic Functional Enrichment to Improve GWAS Power. Am. J. Hum. Genet. *104*, 65–75.
71. Li, X., Ito, M., Zhou, F., Youngson, N., Zuo, X., Leder, P., and Ferguson-Smith, A.C. (2008). A maternal-zygotic effect gene, Zfp57, maintains both maternal and paternal imprints. Dev. Cell *15*, 547–557.
72. Plant, K., Fairfax, B.P., Makino, S., Vandiedonck, C., Radhakrishnan, J., and Knight, J.C. (2014). Fine mapping genetic determinants of the highly variably expressed MHC gene ZFP57. Eur. J. Hum. Genet. *22*, 568–571.
73. Mogil, L.S., Andaleon, A., Badalamenti, A., Dickinson, S.P., Guo, X., Rotter, J.I., Johnson, W.C., Im, H.K., Liu, Y., and Wheeler, H.E. (2018). Genetic architecture of gene expression traits across diverse populations. PLoS Genet. *14*, e1007586.
74. Tehranchi, A., Hie, B., Dacre, M., Kaplow, I., Pettie, K., Combs, P., and Fraser, H.B. (2019). Fine-mapping *cis*-regulatory variants in diverse human populations. eLife *8*, e39595.
75. Liu, X., Li, Y.I., and Pritchard, J.K. (2019). Trans Effects on Gene Expression Can Drive Omnigenic Inheritance. Cell *177*, 1022–1034.e6.
76. Favé, M.-J., Lamaze, F.C., Soave, D., Hodgkinson, A., Gauvin, H., Bruat, V., Grenier, J.-C., Gbeha, E., Skead, K., Smargiassi, A., et al. (2018). Gene-by-environment interactions in urban populations modulate risk phenotypes. Nat. Commun. *9*, 827.
77. Sella, G., and Barton, N.H. (2019). Thinking About the Evolution of Complex Traits in the Era of Genome-Wide Association Studies. Annu. Rev. Genomics Hum. Genet. *20*, 461–493.
78. Akey, J.M. (2009). Constructing genomic maps of positive selection in humans: where do we go from here? Genome Res. *19*, 711–722.

The American Journal of Human Genetics, Volume *109*

Supplemental information

Genetic architecture of gene regulation in Indonesian populations identifies QTLs associated with global and local ancestries

Heini M. Natri, Georgi Hudjashov, Guy Jacobs, Pradiptajati Kusuma, Lauri Saag, Chelzie Crenna Darusallam, Mait Metspalu, Herawati Sudoyo, Murray P. Cox, Irene Gallego Romero, and Nicholas E. Banovich

Supplementary Information

Supplementary Notes 1–4 and Supplementary Figures 1–13

Supplementary Figure 1. Proportions of inferred Papuan ancestry and Denisovan introgression are highly correlated (Pearson's correlation coefficient 0.995).

Supplementary Figure 2. Enrichment of methylQTLs (a) and eQTLs (b) among DNase hypersensitive sites (DHS) and histone marks in ENCODE GM12878 and K562 cell lines.

Supplementary Note 1. Colocalized *cis*-eQTLs and *cis*-methylQTLs indicate shared causal variants.

Supplementary Figure 3. Distribution of the lower bounds of the prior probabilities (p12) that suggest colocalization across 4,639 tested methylQTL-eQTL pairs. As the posterior probability for colocalization is dependent on the prior probability, a post-hoc sensitivity analysis was used to determine the range of prior probabilities for which colocalization is supported. Pairs passing the colocalization threshold with a range of p12 values from $<1.0\times10^{-6}$ to 1.0×10^{-4} (lower bound of p12 below 1.0×10^{-6}) were considered to show robust support for colocalization.

Supplementary Figure 4. Genetically regulated promoter methylation alters target gene expression levels. Relationship between the absolute effect sizes of colocalized methylQTLs and eQTLs that show the same direction of effect (a) and opposing direction of effect (b) on the target trait. Pairs that share the same top-SNP are plotted and variants located on promoter regions are highlighted. Smoothed means based on linear models in the form y ~ x and 95% confidence intervals are shown for each set.

Supplementary Figure 5. Manhattan plots of the eQTL -log10(p-values) for a colocalized gene and an Indonesia-specific gene in the Indonesian data and three European eQTL datasets.

Supplementary Figure 6. Effect sizes of colocalized eQTLs in the Indonesian and European datasets.

Supplementary Figure 7. Expression levels of colocalized and Indonesia-specific eGenes in the Indonesian data and GTEx whole blood data.

Supplementary Figure 8. Relationship between absolute differences in ALT allele frequencies and expression levels of the Indonesia-specific eQTLs between Indonesia and Europe.

Supplementary Note 2. Qualities of eQTLs driven by archaic ancestry.

Supplementary Figure 9. Minor allele frequencies (MAF) and absolute effect sizes of eQTLs driven by Denisovan or Neanderthal introgression and eQTLs not driven by archaic introgression ("other") before (a) and after (b, c) allele frequency matching. t-test p-values are indicated for each pairwise comparison.

Supplementary Figure 10. Minor allele frequencies (MAF) and absolute effect sizes of methylQTLs driven by Denisovan or Neanderthal introgression and methylQTLs not driven by archaic introgression ("other") before (a) and after (b, c) allele frequency matching. t-test p-values are indicated for each pairwise comparison.

Supplementary Figure 11. a: Power to detect QTLs as a function of MAF when N=115. b: Minimum detectable slope in simple linear regression as a function of MAF, with various power levels. In both models, the type I error rate was set to 0.01 and the SD of the linear model to 0.2.

Supplementary Note 3. Identifying ancestry-driven QTLs under positive selection.

Supplementary Figure 12. Modern ancestry and archaic introgression -driven eQTLs overlapping genomic windows that show evidence of recent positive selection in each of the three study populations. Variance in QTL genotype explained (R2) is shown on the x-axis of each plot. Variants with R2 > 0.7 were considered to be highly correlated with ancestry (vertical line). The proportion of positions within 50Kb windows that show an

nSL > 2 is shown on the y-axis. Genomic windows with this proportion >0.3 were considered to be showing evidence of positive selection (horizontal line). The target genes of eQTLs showing both a significant correlation with ancestry and evidence of selection are labeled.

Supplementary Figure 13. Modern ancestry and archaic introgression -driven methylQTLs overlapping genomic windows that show evidence of recent positive selection in each of the three study populations. Variance in QTL genotype explained (R2) is shown on the x-axis of each plot. Variants with R2 > 0.7 were considered to be highly correlated with ancestry (vertical line). The proportion of positions within 50Kb windows that show an nSL > 2 is shown on the y-axis. Genomic windows with this proportion >0.3 were considered to be showing evidence of positive selection (horizontal line). The target CpGs of methylQTLs showing both a significant correlation with ancestry and evidence of selection are labeled.

Supplementary Note 4. Qualities of the Denisovan driven methylQTLs that colocalize with platelet count GWAS.

Supplementary Tables 1–10

Supplementary Table 1: Sample information.

Supplementary Table 2: Sequencing batch information for RNAseq samples.

Supplementary Table 3: Sub-Saharan African samples used in archaic introgression inference.

Supplementary Table 4. Numbers of colocalized and non-colocalized genes in each pairwise comparison between Indonesia and the European cohorts. eGenes with an FDR-p<0.1 were included in testing. The analysis was carried out separately using the top genes from each dataset. Proportions of robustly colocalized genes out of all tested genes are indicated in percentage.

Supplementary Table 5. Descriptions of the target genes of Indonesian eQTLs that show no evidence of colocalization with any of the tested European datasets and harbor an Indonesia-specific eQTL according to the *mashr* analysis.

Supplementary Table 6: p-values for overrepresentation (Fisher's test) of modern and archaic LAI -driven QTLs among the genomic windows under positive selection in each population. QTLs with an FDR-p < 0.01 in the permutation-based analysis were included in testing. QTLs driven by Denisovan or Neanderthal ancestry with an R2>0.7 were considered LAI driven. Windows with a proportion of SNPs with an absolute nSL > 2 of 30% were considered to be under positive selection.

Supplementary Table 7: Ancestry-driven QTLs under positive selection. For methylQTLs, annotations based on the Illumina EPIC array manifest are provided.

Supplementary Table 8: Summary of Indonesian eQTLs and methylQTLs that colocalize with each of the 36 hematological GWAS traits reported by Astle et al. 2016.

Supplementary Table 9. Summary of European eQTLs that colocalize with each of the 36 hematological GWAS traits reported in the Astle et al. 2016.

Supplementary Table 10. Qualities of the Denisovan driven methylQTLs that colocalize with platelet count GWAS.

Supplementary Data Files 1–11

Data S1: Permutation-significant eQTLs. Columns: target, chromosome, target start, N of tested SNPs, top-SNP distance to the target, rsID, top-SNP position, slope, nominal *p*, FDR-*p*

Data S2: Permutation-significant methylQTLs. Columns: target, chromosome, target position, N of tested SNPs, top-SNPdistance to the target, rsID, top-SNP position, slope, nominal *p*, FDR-*p*

Data S3: Nominal eQTL statistics. Columns: target, target chromosome, target start, N of tested SNPs, SNP distance to the target, rsID, SNP position, nominal *p*, slope.

Data S4. eQTL-methylQTL colocalization results for robust colocalized pairs. Columns: Target CpG, Target gene, number of tested SNPs, Tag SNP 1 and Tag SNP 2 (testing between all independent signals), PP0, PP1, PP2, PP3, PP4, PP4/PP3, lower bound of prior probability for colocalization (p12) that passes the threshold.

Data S5: eQTL-LAI correlation results for Papuan ancestry. Columns: chr, pos, R^2, p, target

Data S6: eQTL-LAI correlation results for Denisovan introgression. Columns: chr, pos, R^2, p, target

Data S7: eQTL-LAI correlation results for Neanderthal introgression. Columns: chr, pos, R^2, p, target

Data S8: methylQTL-LAI correlation results for Papuan ancestry. Columns: chr, pos, R^2, p, target

Data S9: methylQTL-LAI correlation results for Denisovan introgression. Columns: chr, pos, R^2, p, target

Data S10: methylQTL-LAI correlation results for Neanderthal introgression. Columns: chr, pos, R^2, p, target

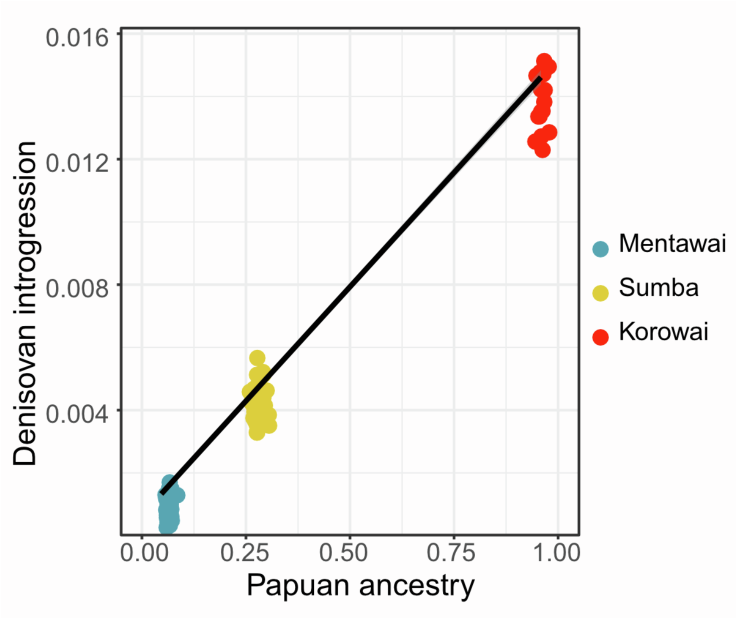

Supplementary Figure 1. Proportions of inferred Papuan ancestry and Denisovan introgression are highly correlated (Pearson's correlation coefficient 0.995).

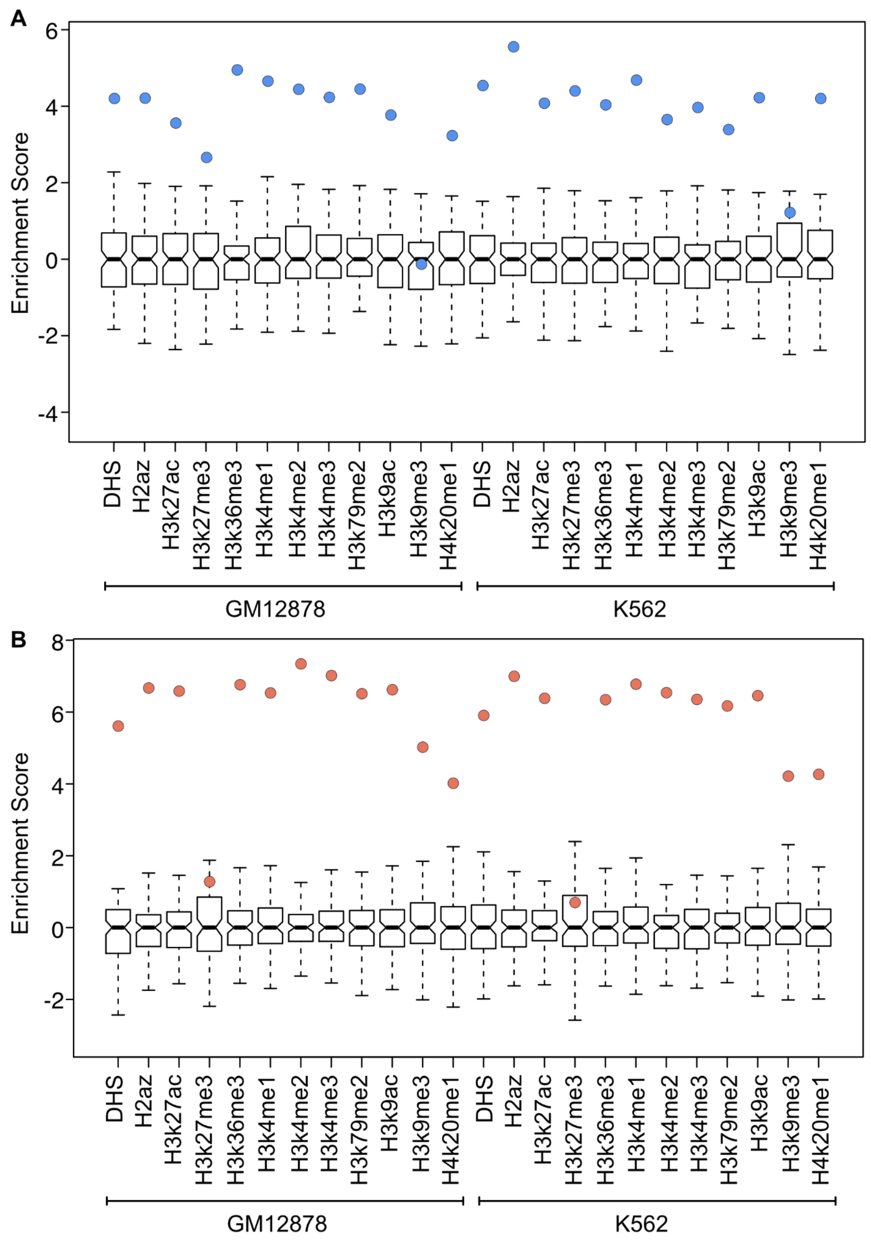

Supplementary Figure 2. Enrichment of methylQTLs (**A**) and eQTLs (**B**) among DNase hypersensitive sites (DHS) and histone marks in ENCODE GM12878 and K562 cell lines.

Supplementary Note 1. ***Colocalized cis-eQTLs and cis-methylQTLs indicate shared causal variants.*** We integrated the methylQTL and eQTL calls to gain insight into how genetic regulation of CpG methylation may contribute to the regulation of gene expression. 1,140 of the unique permutation significant eVariants were also nominally associated (nominal $p<1\times10^{-7}$) with the methylation of at least one CpG, and 2,015 of the unique permutation-significant methylVariants were also nominally associated with the expression of at least one gene,

suggesting that a substantial number of causal eVariants may also be causal methylVariants, and vice versa. This overlap corresponds to 4,639 CpG-gene combinations potentially harboring a common causal variant (CCV). We tested for colocalization between these pairs of CpGs and genes using a Bayesian method as implemented in *coloc* v4. Among the tested pairs, we detected 720 (15.5%) eQTL-methylQTL pairs that showed robust support for colocalization with a wide range of prior probabilities for a common causal variant (Supplementary Figure 3, Methods).

We explored the direction of the effects of top-SNPs associated with the 720 CpG and gene pairs that exhibit a high probability of a single shared causal variant. Concordant with previous studies[1,2], 56.1% of these eQTL-methylQTL pairs show an opposite effect direction. This proportion is 61.9% when only including pairs that had the same top-SNP based on QTL *p*-values, and 69.1% when further limiting to CpGs that are located on promoter regions. Pairs that show an opposite effect also show a high correlation between the absolute effect sizes (Pearson's correlation 0.49, $p < 2.2\times10^{-16}$), while pairs with the same effect directions don't (Pearson's correlation 0.03, p=0.64) (Supplementary Figure 4). Colocalized CpGs located on promoters are more likely to show an opposite direction in effect with the gene than CpGs located outside promoters or enhancers (Fisher's test p=3.835×10^{-6}), but the same is not observed for CpGs located on enhancers when compared to those located outside promoters or enhancers (Fisher's test p=0.6808).

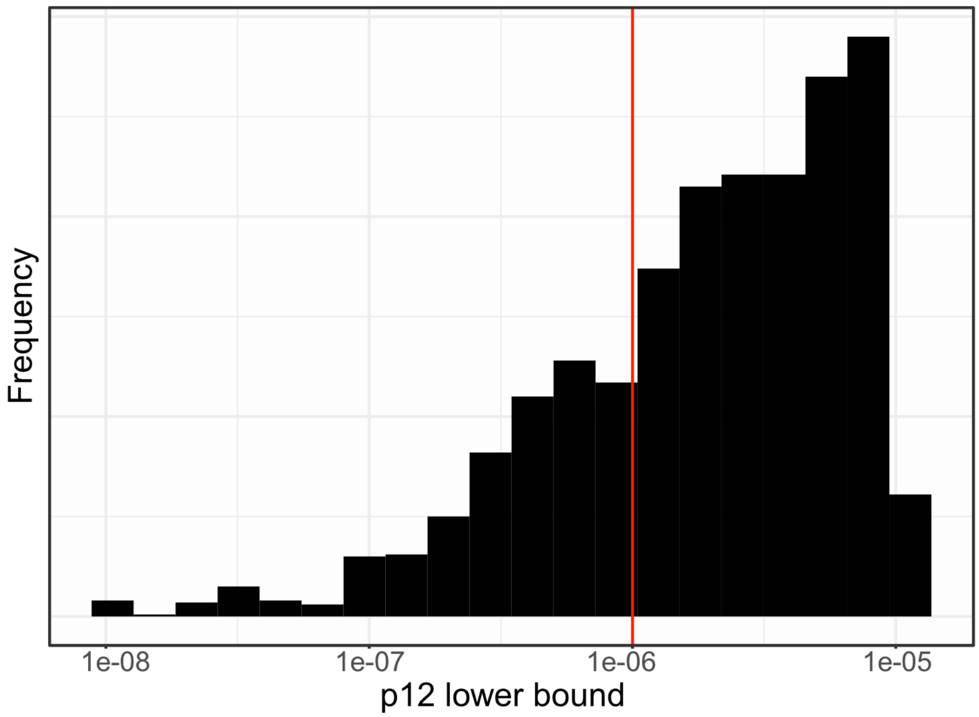

Supplementary Figure 3. Distribution of the lower bounds of the prior probabilities (p12) that suggest colocalization across 4,639 tested methylQTL-eQTL pairs. As the posterior probability for colocalization is dependent on the prior probability, a post-hoc sensitivity analysis was used to determine the range of prior probabilities for which colocalization is supported. Pairs passing the colocalization threshold with a range of p12 values from $<1.0\times10^{-6}$ to 1.0×10^{-4} (lower bound of p12 below 1.0×10^{-6}) were considered to show robust support for colocalization.

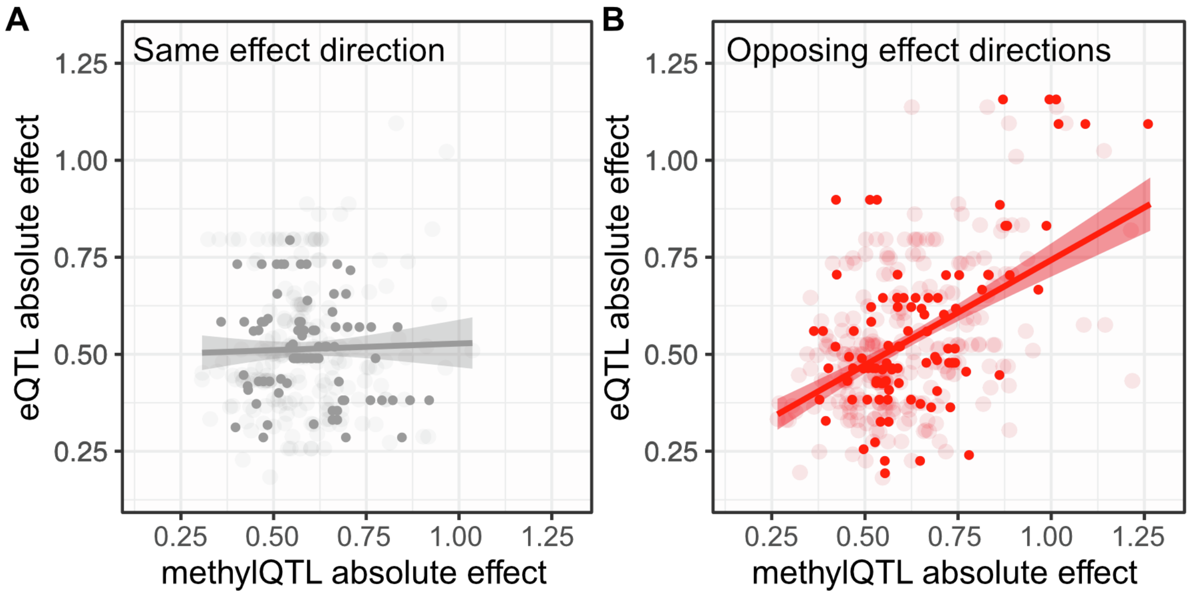

Supplementary Figure 4. Genetically regulated promoter methylation alters target gene expression levels. Relationship between the absolute effect sizes of colocalized methylQTLs and eQTLs that show the same direction of effect (**A**) and opposing direction of effect (**B**) on the target trait. Pairs that share the same top-SNP are plotted and variants located on promoter regions are highlighted. Smoothed means based on linear models in the form y ~ x and 95% confidence intervals are shown for each set.

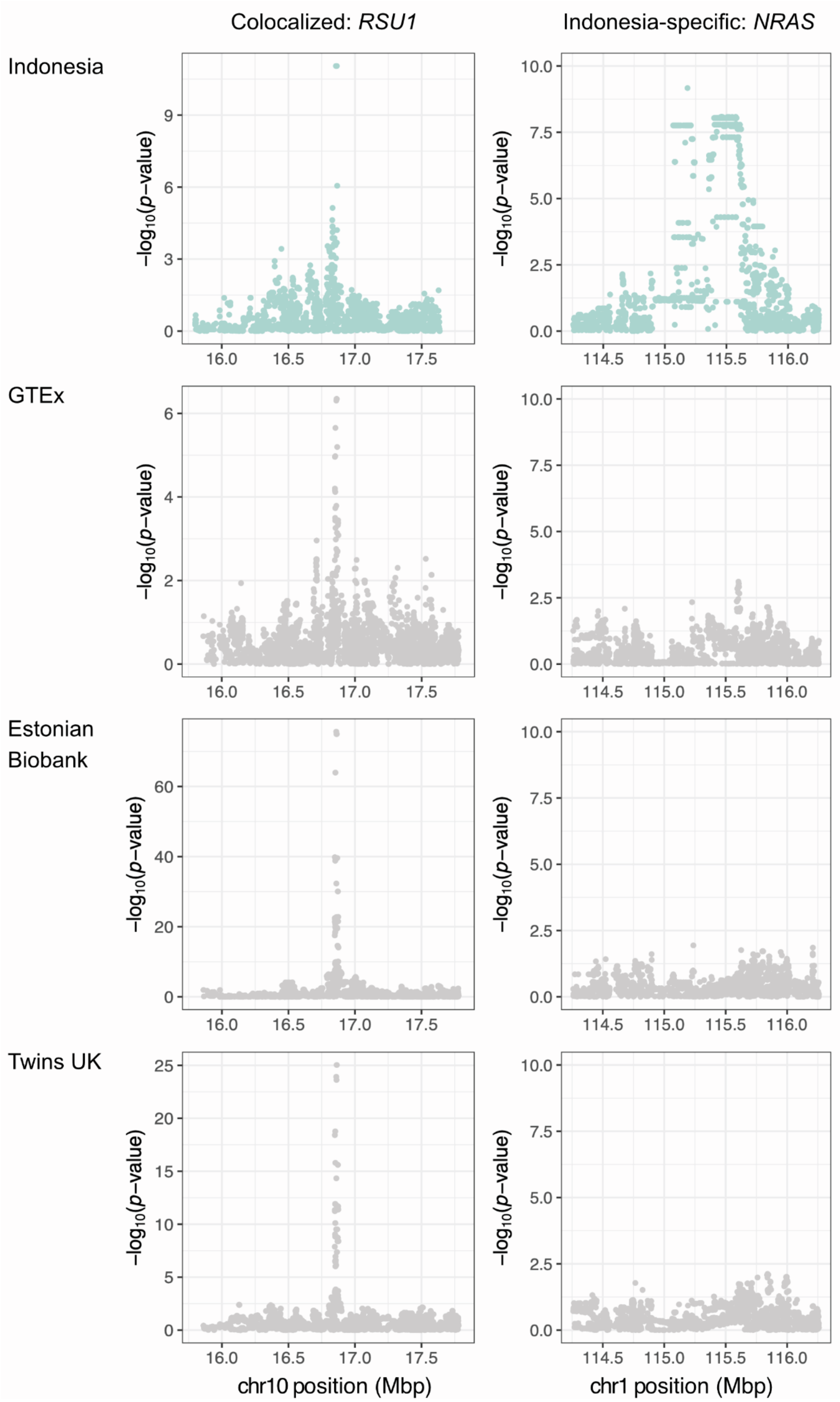

Supplementary Figure 5. Manhattan plots of the eQTL -log10(*p*-values) for a colocalized gene and an Indonesia-specific gene in the Indonesian data and three European eQTL datasets.

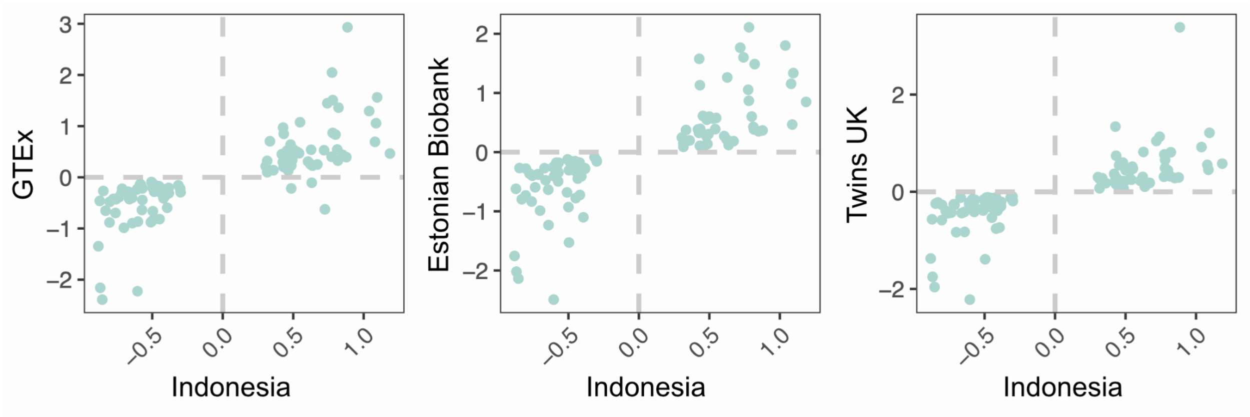

Supplementary Figure 6. Effect sizes of colocalized eQTLs in the Indonesian and European datasets.

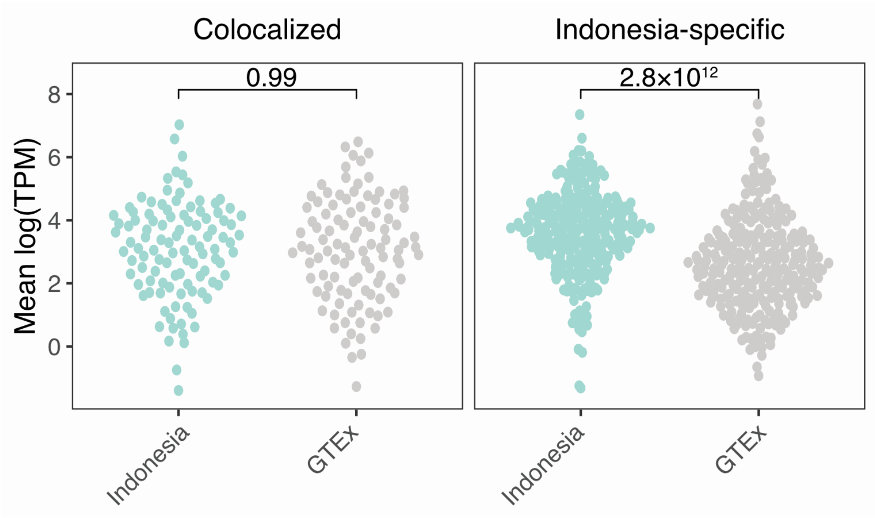

Supplementary Figure 7. Expression levels of colocalized and Indonesia-specific eGenes in the Indonesian data and GTEx whole blood data.

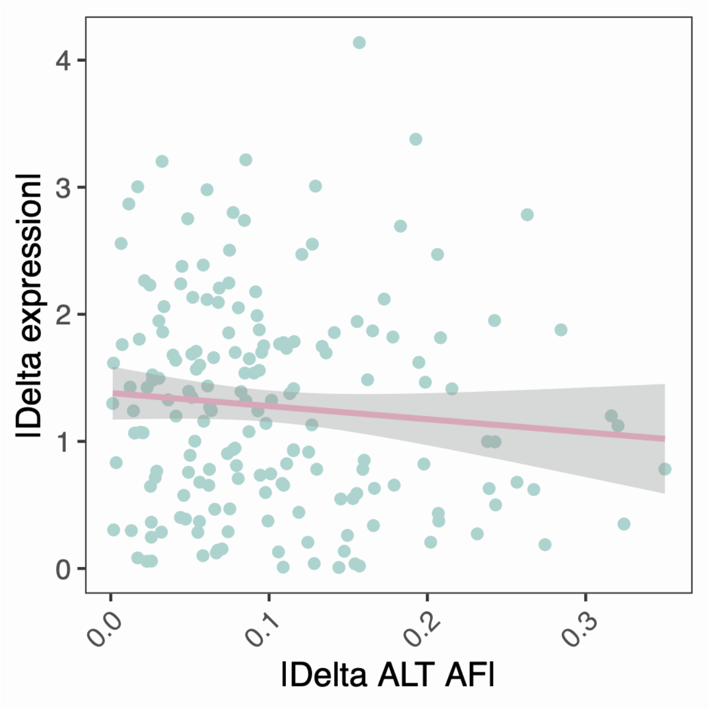

Supplementary Figure 8. Relationship between absolute differences in ALT allele frequencies and expression levels of the Indonesia-specific eQTLs between Indonesia and Europe.

Supplementary Note 2. *Qualities of eQTLs driven by archaic ancestry.* We compared the absolute effect sizes of the archaic ancestry-driven QTLs and the effect sizes of the significant QTLs not driven by archaic ancestry. Denisovan and Neanderthal ancestry-driven eQTLs (Supplementary Figure 9) and methylQTLs (Supplementary Figure 10) exhibit significantly larger absolute effect sizes than methylQTLs not driven by archaic ancestry. However, as the minor allele frequencies of the archaic driven QTLs are lower, we are less powered to detect small effect QTLs driven by archaic ancestry (Supplementary Figure 11). We performed allele frequency matching with the nearest neighbor matching method of the R package *MatchIt* v3.0.2[3]. There were no significant differences in the mean absolute effect sizes of the MAF matched sets and the archaic driven QTLs.

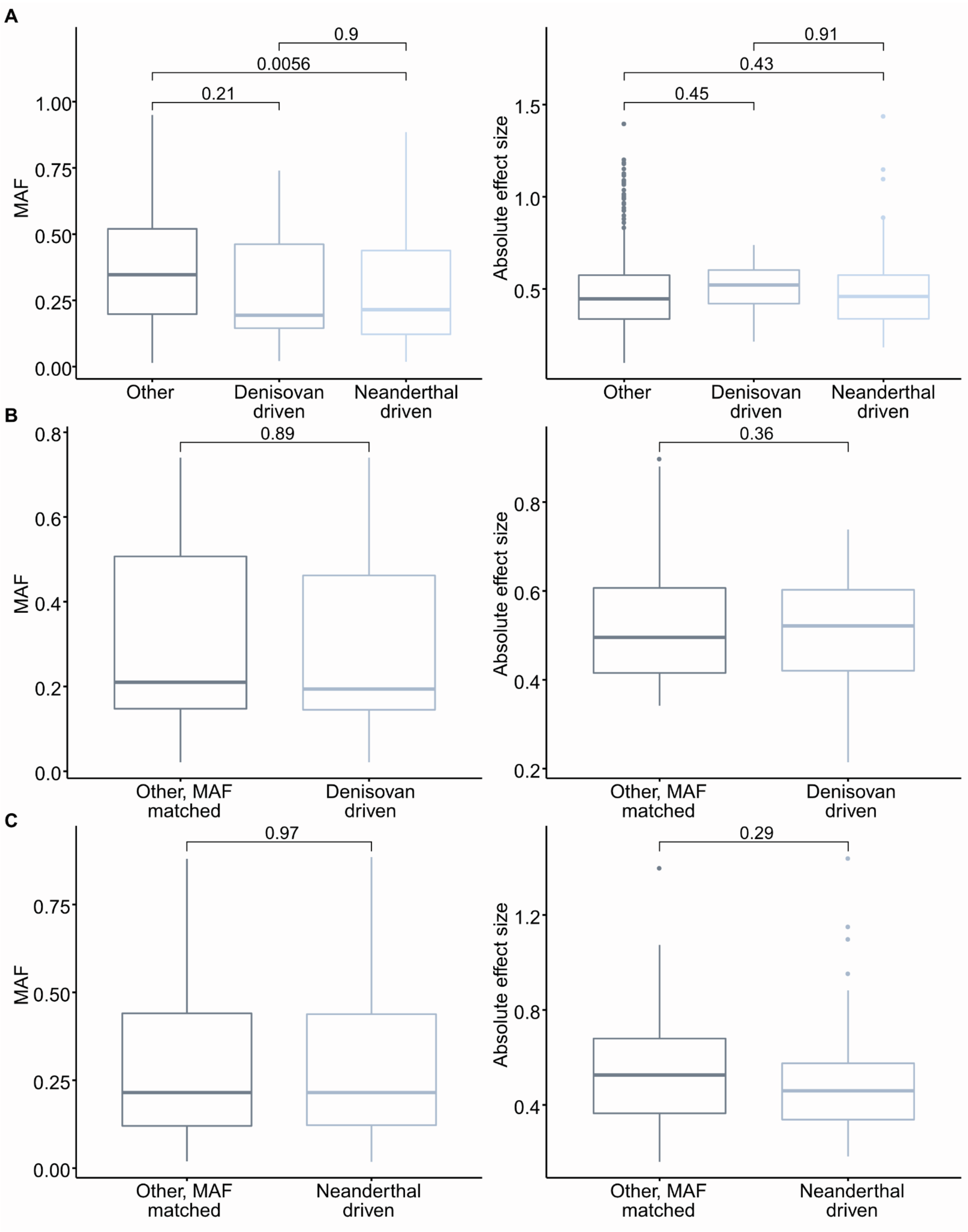

Supplementary Figure 9. Minor allele frequencies (MAF) and absolute effect sizes of eQTLs driven by Denisovan or Neanderthal introgression and eQTLs not driven by archaic

introgression ("other") before (**A**) and after (**B**, **C**) allele frequency matching. t-test *p*-values are indicated for each pairwise comparison.

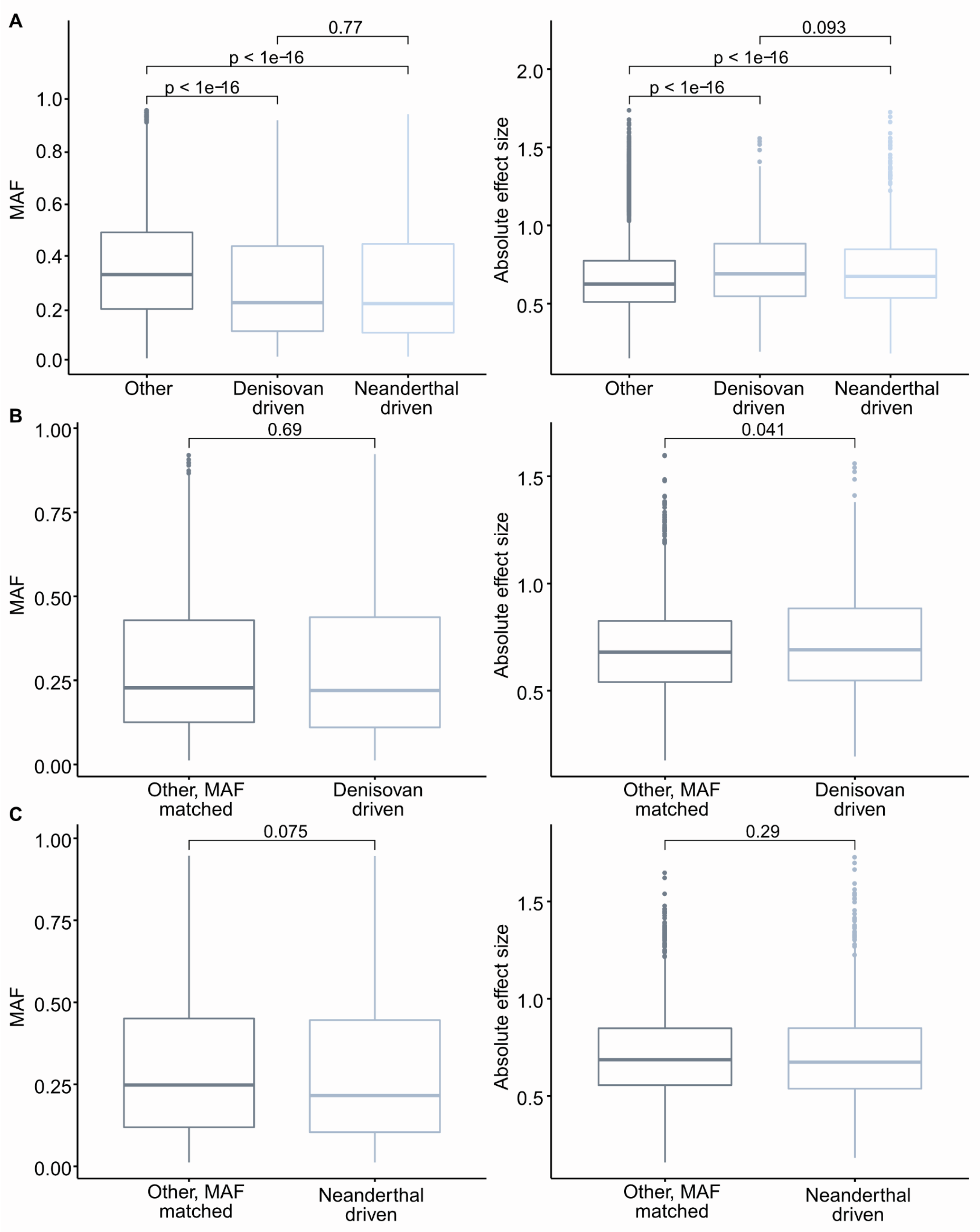

Supplementary Figure 10. Minor allele frequencies (MAF) and absolute effect sizes of methylQTLs driven by Denisovan or Neanderthal introgression and methylQTLs not driven by archaic introgression ("other") before (**A**) and after (**B**, **C**) allele frequency matching. t-test *p*-values are indicated for each pairwise comparison.

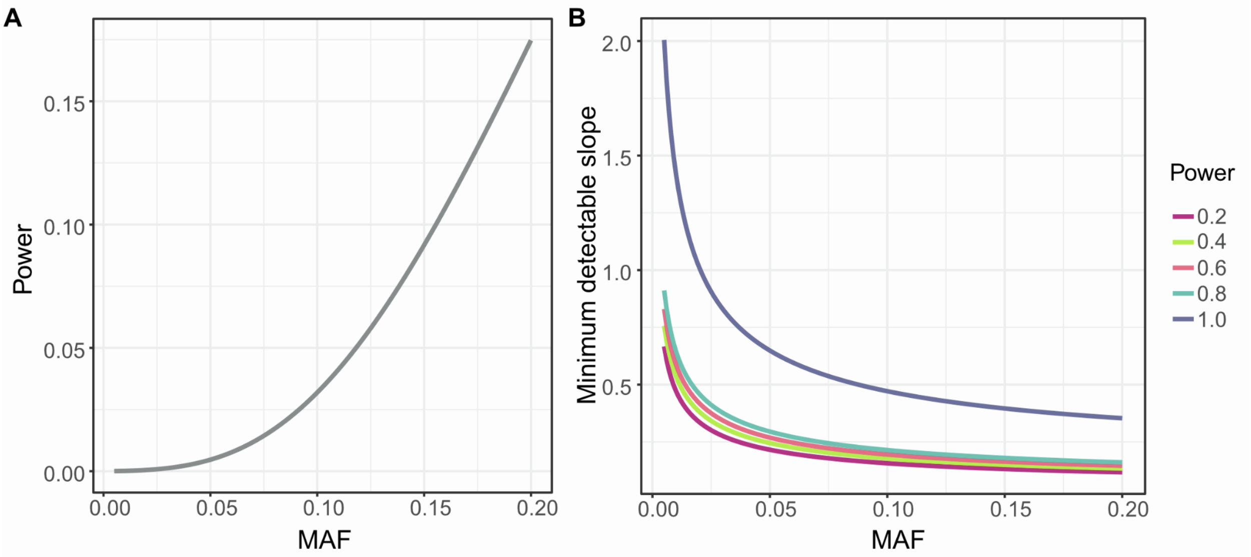

Supplementary Figure 11. A: Power to detect QTLs as a function of MAF when N=115. **B:** Minimum detectable slope in simple linear regression as a function of MAF, with various power levels. In both models, the type I error rate was set to 0.01 and the SD of the linear model to 0.2.

Supplementary Note 3. ***Identifying ancestry-driven QTLs under positive selection.*** We asked whether positive selection on ancestry informative regulatory variants may have contributed to the between-population variation in molecular phenotypes in the region. We used a haplotype-based nSL selection scan (Methods) to identify genomic regions that show signs of past selective sweeps and found 4.7%, 4.6%, and 5.0% of the genome to be under positive selection in Mentawai, Sumba, and Korowai, respectively. We used a colocalization-based method (Methods) to identify shared signals between the QTLs and nSL and detect no significant overlap between QTLs and selection.

Additionally, we overlapped the ancestry-driven QTLs with genomic regions with strong evidence of positive selection. We detect no clear overrepresentation of ancestry-driven QTLs among these regions (Supplementary Table 5). However, we find individual QTLs that overlap them (Supplementary Table 6, Supplementary Figures 10, 11), including one Papuan-driven

eQTL under selection in Mentawai, one Neanderthal-driven eQTL in Korowai, and one in Mentawai, as well as Papuan-driven methylQTLs under selection in Mentawai (2), Sumba (2), and Korowai (12).

Moreover, we detect one Denisovan-driven methylQTL under selection in Korowai, associated with a CpG located on the promoter of *ZNF426*. Genetic variation associated with *ZNF426* and other KRAB-ZNF genes has previously been identified on candidate regions for positive selection in multiple human populations[4,5]. Further, we identified 13, 6, and 3 Neanderthal-driven methylQTLs under selection in Mentawai, Sumba, and Korowai (Supplementary Table 6). For example, a Neanderthal-driven methylQTL under selection in Mentawai was also nominally associated (p = 2.596×10^{-7}) with *CATSPER3* (Cation Channel Sperm Associated 3) expression, which was differentially expressed between Mentawai and Korowai, as well as Sumba and Korowai[6]. Neanderthal variation in sodium channel genes was recently linked to increased pain sensitivity in modern humans[7].

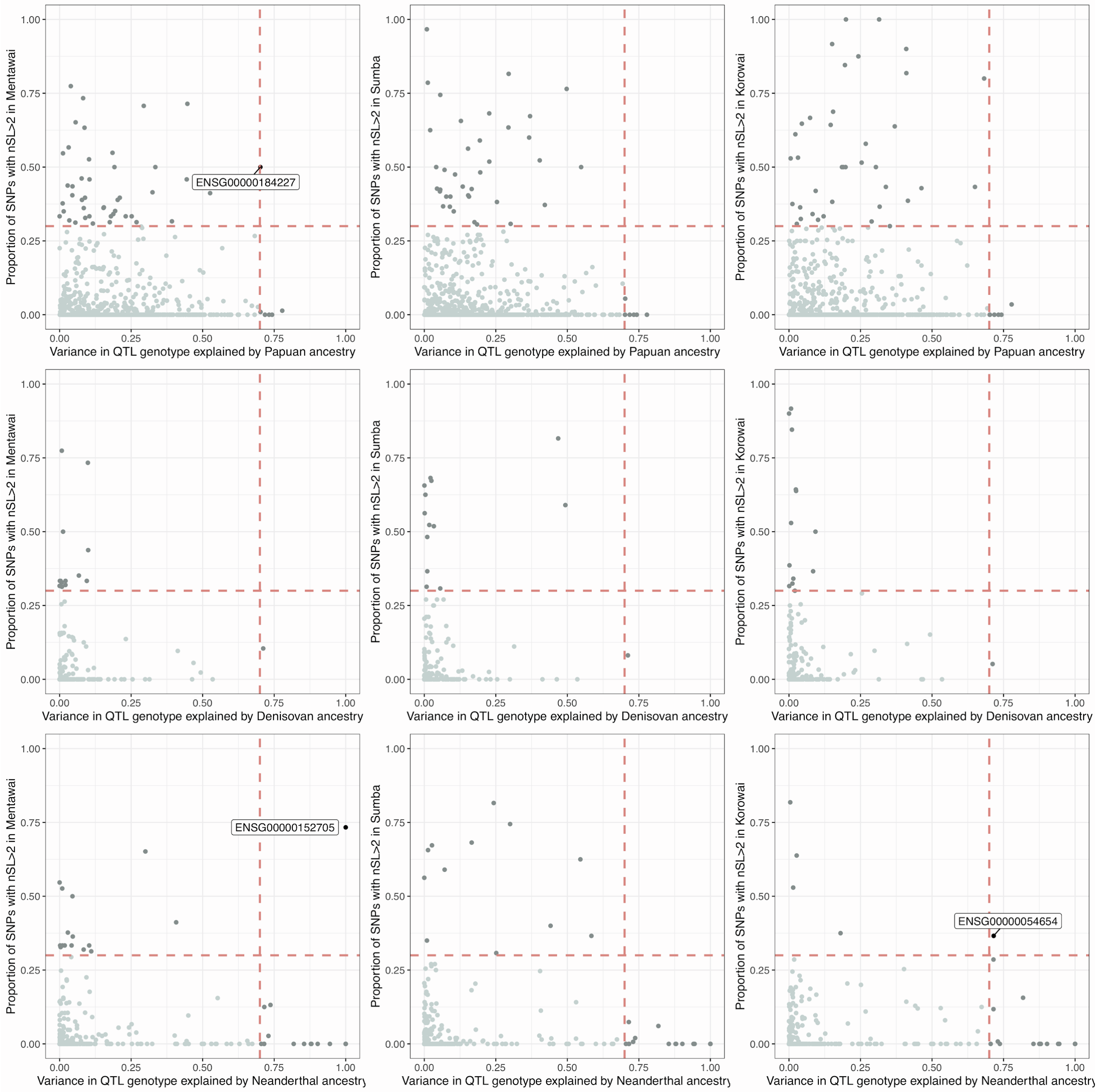

Supplementary Figure 12. Modern ancestry and archaic introgression -driven eQTLs overlapping genomic windows that show evidence of recent positive selection in each of the three study populations. Variance in QTL genotype explained (*R^2*) is shown on the x-axis of each plot. Variants with *R^2* > 0.7 were considered to be highly correlated with ancestry (vertical line). The proportion of positions within 50Kb windows that show an nSL > 2 is shown on the y-axis. Genomic windows with this proportion >0.3 were considered to be showing evidence of positive selection (horizontal line). The target genes of eQTLs showing both a significant correlation with ancestry and evidence of selection are labeled.

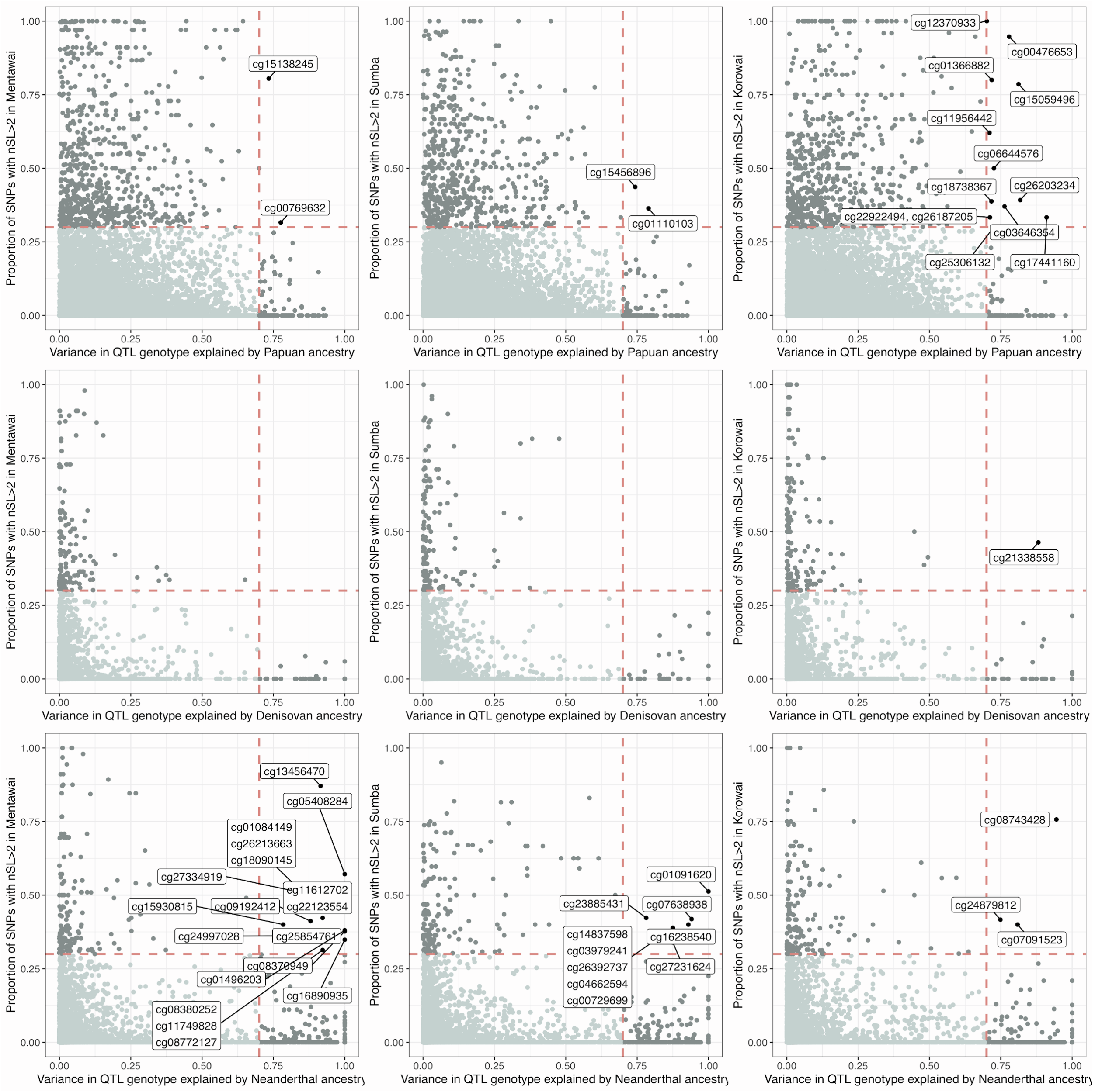

Supplementary Figure 13. Modern ancestry and archaic introgression driven methylQTLs overlapping genomic windows that show evidence of recent positive selection in each of the three study populations. Variance in QTL genotype explained (R^2) is shown on the x-axis of each plot. Variants with $R^2 > 0.7$ were considered to be highly correlated with ancestry (vertical line). Proportion of positions within 50Kb windows that show an nSL > 2 is shown on the y-axis. Genomic windows with this proportion >0.3 were considered to be showing evidence of positive selection (horizontal line). The target CpGs of methylQTLs showing both a significant correlation with ancestry and evidence of selection are labeled.

Supplementary Note 4. Qualities of the Denisovan-driven GWAS-eQTLs. We assessed the credibility of the four Denisovan-driven methylQTLs that colocalize with platelet count GWAS loci. First, we assessed our ability to correctly call genotypes on these positions, to correctly call methylQTLs, and to identify the correlation between the genotypes and the numbers of inferred Denisovan alleles. We used mappability scores generated with Umap[8] to assess mappability on regions overlapping these methylVariants. Umap calculates the single-read mappability of genome for a range of sequencing read lengths, the single-read mappability of a genomic region being defined as a fraction of that region that overlaps with at least one uniquely mappable kmer. For a given sequence, mappability of 1 means that the sequence is uniquely mappable on the forward strand. Uniquely mappable regions with various kmers were downloaded from the Hoffman Lab website (Web Resources). All four variants are located on regions that are uniquely mappable with kmer lengths of 24, 36, 50, and 100bp, apart from chr6:29,799,383 which is on a region that is only uniquely mappable with kmers 36, 50, and 100bp. All four variants were called with high read depth, ranging from 29,492 to 37,373. All four variants have adequate MAFs, ranging from 0.161 to 0.302. All four methylQTLs show large effect sizes, the absolute effect size ranging from 0.66 to 0.88. Furthermore, all methylVariants show a clear correlation with the number of inferred Denisovan alleles, R^2 ranging from 0.73 to 0.90. The methylVariants associated with cg03118604 and cg03861427 are located within 741bp of each other and are in LD.

Then, we assessed whether sequence similarity across the genome could lead to spurious signals in the CpG methylation measurements using the Illumina EPIC array. We used megablast of BLASTN[9] to map the forward sequences flanking the CpGs to the human reference genome. All four sequences map to the HLA locus with high confidence and do not map to other regions (Supplementary Table 10).

References

1. Pierce, B.L., Tong, L., Argos, M., Demanelis, K., Jasmine, F., Rakibuz-Zaman, M., Sarwar, G., Islam, M.T., Shahriar, H., Islam, T., et al. (2018). Co-occurring expression and methylation QTLs allow detection of common causal variants and shared biological mechanisms. Nat. Commun. *9*, 804.

2. Banovich, N.E., Lan, X., McVicker, G., van de Geijn, B., Degner, J.F., Blischak, J.D., Roux, J., Pritchard, J.K., and Gilad, Y. (2014). Methylation QTLs are associated with coordinated changes in transcription factor binding, histone modifications, and gene expression levels. PLoS Genet. *10*, e1004663.

3. Ho, D.E., Imai, K., King, G., and Stuart, E.A. (2011). MatchIt: Nonparametric Preprocessing for Parametric Causal Inference. Journal of Statistical Software *42*,.

4. Perdomo-Sabogal, Á., and Nowick, K. (2019). Genetic Variation in Human Gene Regulatory Factors Uncovers Regulatory Roles in Local Adaptation and Disease. Genome Biol. Evol. *11*, 2178–2193.

5. Ávila-Arcos, M.C., McManus, K.F., Sandoval, K., Rodríguez-Rodríguez, J.E., Villa-Islas, V., Martin, A.R., Luisi, P., Peñaloza-Espinosa, R.I., Eng, C., Huntsman, S., et al. (2020). Population History and Gene Divergence in Native Mexicans Inferred from 76 Human Exomes. Mol. Biol. Evol. *37*, 994–1006.

6. Natri, H.M., Bobowik, K.S., Kusuma, P., Crenna Darusallam, C., Jacobs, G.S., Hudjashov, G., Lansing, J.S., Sudoyo, H., Banovich, N.E., Cox, M.P., et al. (2020). Genome-wide DNA methylation and gene expression patterns reflect genetic ancestry and environmental differences across the Indonesian archipelago. PLoS Genet. *16*, e1008749.

7. Zeberg, H., Dannemann, M., Sahlholm, K., Tsuo, K., Maricic, T., Wiebe, V., Hevers, W., Robinson, H.P.C., Kelso, J., and Pääbo, S. (2020). A Neanderthal Sodium Channel Increases Pain Sensitivity in Present-Day Humans. Curr. Biol.

8. Karimzadeh, M., Ernst, C., Kundaje, A., and Hoffman, M.M. (2018). Umap and Bismap: quantifying genome and methylome mappability. Nucleic Acids Res. *46*, e120.

9. Zhang, Z., Schwartz, S., Wagner, L., and Miller, W. (2000). A greedy algorithm for aligning

DNA sequences. J. Comput. Biol. *7*, 203–214.
